# Novel Polycarbo-Substituted Imidazo[1,2-*c*]quinazolines: Synthesis and Cytotoxicity Study

**DOI:** 10.3390/molecules201219863

**Published:** 2015-12-15

**Authors:** Tebogo Ankie Khoza, Tshepiso Jan Makhafola, Malose Jack Mphahlele

**Affiliations:** 1 Department of Chemistry, College of Science, Engineering and Technology, University of South Africa, P. O. Box 392, Pretoria 0003, South Africa; khozata@unisa.ac.za; 2 Department of Life and Consumer Sciences, College of Agriculture and Environmental Sciences, University of South Africa, Private Bag X06, FL 1710, South Africa; makhat@unisa.ac.za

**Keywords:** dihalogenated 2*H*-imidazo[1,2-*c*]quinazolines, cross-coupling, imidazo[1,2-*c*]quinazolines, cytotoxicity

## Abstract

Amination of the 2-aryl-6-bromo-4-chloro-8-iodoquinazolines with 2-aminoethanol followed by acid-promoted cyclodehydration of the incipient 2-((6,8-dihalo-2-phenylquinazolin-4-yl)amino)ethanols afforded the corresponding novel 5-aryl-9-bromo-7-iodo-2,3-dihydro-2*H*-imidazo[1,2-*c*]quinazolines. The latter were, in turn, subjected to sequential (Sonogashira and Suzuki-Miyaura) and one-pot two-step (Sonogashira/Stille) cross-coupling reactions to afford diversely functionalized polycarbo-substituted 2*H*-imidazo[1,2-*c*]quinazolines. The imidazoquinazolines were screened for *in vitro* cytotoxicity against human breast adenocarcinoma (MCF-7) cells and human cervical cancer (HeLa) cells.

## 1. Introduction

**Figure 1:**
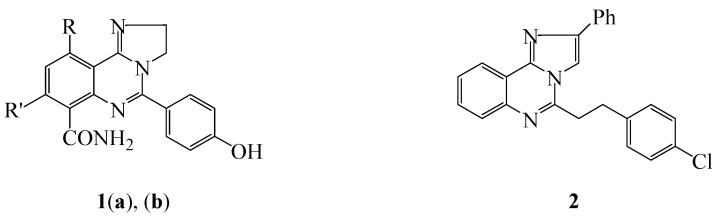
Examples of biologically-relevant imidazo[1,2-*c*]quinazolines. **1**: R = -C_6_H_5_, R′ = -CF_3_ (**a**); R = -CF_3_, R′ = -C_6_H_5_ (**b**) and **2**.

Imidazo[1,2-*c*]quinazoline-based compounds continue to attract attention in synthesis because of their application in pharmaceuticals and materials [1]. A series of 5-alkyl substituted imidazo[1,2-*c*]quinazolines, for example, were screened for *in vitro* and *in vivo* bronchodilatory activity and the trend in activity was found to increase with increasing alkyl chain (methyl < ethyl < propyl) [2]. The presence of halogen atom on the 7- and/or 9-position, on the other hand, was found to increase bronchodilatory activity over unsubstituted derivatives and revealed the following trend in activity: hydrogen < monobromo < dibromo < iodo [2]. Polycarbo-substituted 2,3-dihydro-2*H*-imidazo[1,2-*c*]quinazoline **1a** [3,4] (Figure 1) and its 10-phenyl-8-trifluoromethyl isomer **1b** [4] have been found to exhibit anti-inflammatory activity and to bind to cyclooxygenase isoenzyme (COX-1 and COX-2) of rat paw edema. The 5-(4-chlorostyryl)-2-phenylimidazo[1,2-*c*] quinazoline **2**, on the other hand, was found to exhibit significant anti-cancer activity against HEP-G2 liver cell line [5]. The iridium and platinum cyclometalated imidazo[1,2-*c*]quinazolines have also been patented as efficient dopants for organic electroluminescent layers in organic light emitting diodes (OLEDS) [6].

The two main synthetic approaches towards imidazo[1,2-*c*]quinazolines involve either annulation of quinazoline moiety onto an imidazole framework or a two-step assembly of the imidazole ring onto a quinazoline framework. Korshin *et al.* reacted 2-(2-aminophenyl)-4,5-dihydro-1*H*-imidazole with aldehydes to afford novel 5-substituted 2,3,5,6-tetrahydroimidazo[1,2-*c*]quinazolines, which were in turn, subjected to dehydrogenation with one or two equivalent of KMnO_4_-silica gel mixture in acetonitrile at 0 °C or r.t. to afford the 2,3-dihydroimidazo[1,2-*c*]quinazolines or their imidazo[1,2-*c*]quinazolines, respectively [1]. The most common and convenient approach for the synthesis of imidazo[1,2-*c*]quinazolines involves a two-step assembly of the imidazole ring onto a quinazoline framework based on 4-chloroquinazoline scaffold [7,8,9]. 4-Chloroquinazoline, for example, was reacted with aziridines followed by iodide-catalyzed rearrangement of the incipient 4-(aziridin-1-yl)quinazoline intermediates to afford the corresponding 2,3-dihydroimidazo[1,2-*c*]quinazolines [7,8]. These compounds were also prepared by amination of 4-chloroquinazolines with chloroethylamine [7,9]. Successive amination of 4-chloroquinazolines with aminoethanol and subsequent cyclodehydration of the incipient 2-[(quinazolin-4-yl)amino]alcohols using thionyl chloride or phosphoryl chloride also afforded novel 2,3-dihydro-2*H*-imidazo[1,2-*c*]quinazolines [2,7,9]. These literature precedents encouraged us to investigate the possibility to synthesize novel polycarbo-substituted 2,3-dihydro-2*H*-imidazo[1,2-*c*]quinazolines based on the 2-aryl-6-bromo-4-chloro-8-iodoquinazolines as precursors for palladium catalyzed C*sp*^2^–C*sp*^2^ and C*sp*^2^–C*sp* bond formation. Herein, we report the results of the reactivity of the 6-aryl-9-bromo-7-iodo-2,3-dihydro-2*H*-imidazo[1,2-*c*]quinazolinesin sequential (Sonogashira/Suzuki-Miyaura) and one-pot two-step (Sonogashira/Stille) cross-coupling reactions to afford diversely functionalized polycarbo-substituted 2*H*-imidazo[1,2-*c*]quinazolines. The compounds were evaluated for *in vitro* cytotoxicity against human breast adenocarcinoma (MCF-7) cells and human cervical cancer (HeLa) cells.

## 2. Results and Discussion

### 2.1. Chemistry

**Scheme 1:**
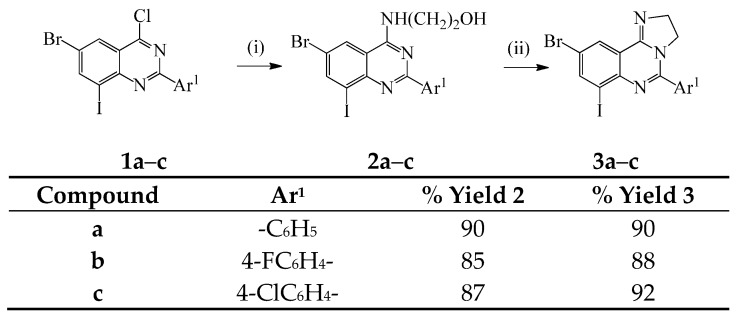
Synthesis of the 6-aryl-8,10-dihalo-3,4-dihydro-2*H*-imidazo[1,2-*c*]quinazolines **3a**–**c**. *Reagents and conditions*: (i) NH_2_CH_2_CH_2_OH, reflux, 2 h; (ii) conc. H_2_SO_4_, 120 °C, 2 h.

In the first part of this investigation, we subjected the known 2-aryl-6-bromo-4-chloro-8-iodoquinazolines **1a**–**c** [10] to dechloro-amination with 1-aminoethanol under reflux for 2 h (Scheme 1). We isolated the corresponding 2-((2-aryl-6,8-dihaloquinazolin-4-yl)amino)ethanols **2a**–**c**, which are easily distinguished from the corresponding precursors by the presence of additional signals in the aliphatic region of their ^1^H- and ^13^C-NMR spectra. Moreover, the molecular ion region of their mass spectra reveal the absence of the M and M+2 peaks in the ratio 3:1 typical for compounds containing the ^35^Cl and ^37^Cl isotopes. Attempted cyclodehydration of **2a**–**c** in excess phosphoryl chloride under reflux followed by cooling and aqueous workup led to the recovery of the starting material. Under the same reaction conditions, the analogous 2-alkyl substituted 4-(1-hydroxyethyl)aminoquinazolines previously afforded the corresponding 5-alkyl-2,3-dihydroimidazo[1,2-*c*]quinazolines in 50%–58% yield [2]. Hitherto, Stankovský and Filip subjected the analogous 2-amino substituted 4-(2-hydroxyethylamino)quinazolines to an excess of phosphoryl chloride under reflux and isolated the corresponding 4-(2-chloroethylamino)quinazolines and the unreacted starting material [11]. The recovered aminoalcohols were rationalized as a consequence of hydrolysis of the corresponding chlorides or the phosphoric acid esters during neutralization. The requisite imidazoquinazolines were, however, isolated as sole products when the 4-(2-hydroxyethylamino)quinazolines when concentrated hydrochloric acid was used as a dehydrating agent at 120 °C. We adapted these reaction conditions to compounds **2a**–**c** and recovered the starting materials unchanged after prolonged heating due to poor solubility of these compounds in concentrated hydrochloric acid. We then opted for the use of a stronger acid as a dehydrating agent and reacted compounds **2a**–**c** with concentrated sulfuric acid at 120 °C for 2 h. To our delight, we isolated the requisite 6-aryl-7,9-dihalo-2,3-dihydro-2*H*-imidazo[1,2-*c*]quinazolines **3a**–**c** in high yield (Scheme 1). The structures of compounds **3a**–**c** were characterized using a combination of NMR and IR spectroscopic techniques as well as mass spectrometry.

**Scheme 2:**
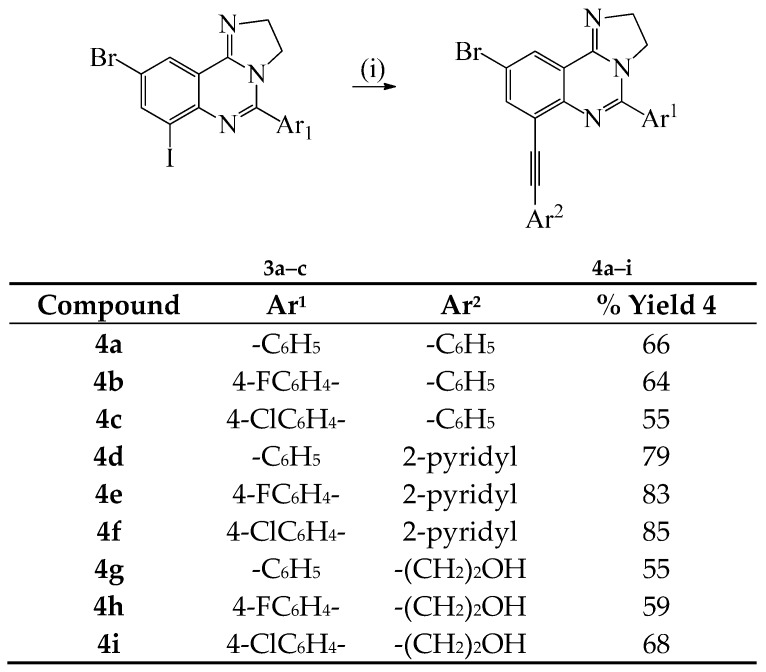
Sonogashira cross-coupling of **3a**–**c** with terminal alkynes to afford **4a**–**i**. *Reagents and conditions*: (i) Ar^2^C≡CH (1.1 equiv.), PdCl_2_(PPh_3_)_2_, CuI, K_2_CO_3_, DMF–EtOH, r.t., 18 h.

**Scheme 3:**
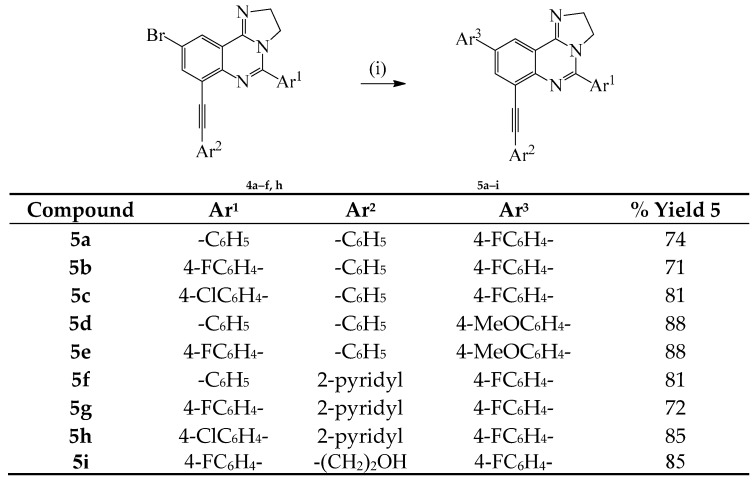
Suzuki cross-coupling of **4a**–**f**, **h** with arylboronic acids. *Reagents and conditions*: (i) Ar^3^B(OH)_2_ (1.2 equiv.), PdCl_2_(PPh_3_)_2_, K_2_CO_3_, 3:1 DMF–EtOH (*v*/*v*), 100 °C, 2 h.

Given the need for the development of efficient methods for the incorporation of carbon-based substituents on quinazoline moiety in pharmaceutical compounds or materials [12], we decided to investigate the reactivity of compounds **3a**–**c** in sequential palladium catalyzed cross-coupling reactions to increase the diversity of substitution on the heterocycle. Compounds **3a**–**c** were subjected to Sonogashira cross-coupling with terminal acetylenes under standard conditions involving the use of dichlorobis(triphenylphosphine)palladium(II)-CuI and K_2_CO_3_ in 3:1 DMF-ethanol (*v*/*v*) mixture at r.t. for 18 h (Scheme 2). We isolated a single mono-substituted quinazoline product characterized using a combination of NMR and IR spectroscopic techniques as **4a** and the reaction conditions were extended to other derivatives using phenylacetylene, 2-pyridylacetylene and 3-propyn-1-ol as coupling partners to afford products **4b**–**i**. The selectivity of cross-coupling through C*sp*^2^–I *versus* C*sp*^2^–Br bond is due to the intrinsic reactivity of the C–I bond in transition metal–mediated cross-coupling reactions, which relates to their relative bond dissociation energy (trend: C*sp*^2^–I < C*sp*^2^–Br) [10]. The monoalkynylated derivatives **4a**–**f**, **h** were, in turn, subjected to the Suzuki-Miyaura cross-coupling with 4-fluorophenylboronic or 4-methoxyphenylboronic acid using PdCl_2_(PPh_3_)_2_–PCy_3_ catalyst complex in the presence of K_2_CO_3_ in 3:1 DMF–ethanol (*v*/*v*) was heated at 100 °C to afford the corresponding unsymmetrical polycarbo-substituted imidazoquinolines **5a**–**i** (Scheme 3).

**Scheme 4:**
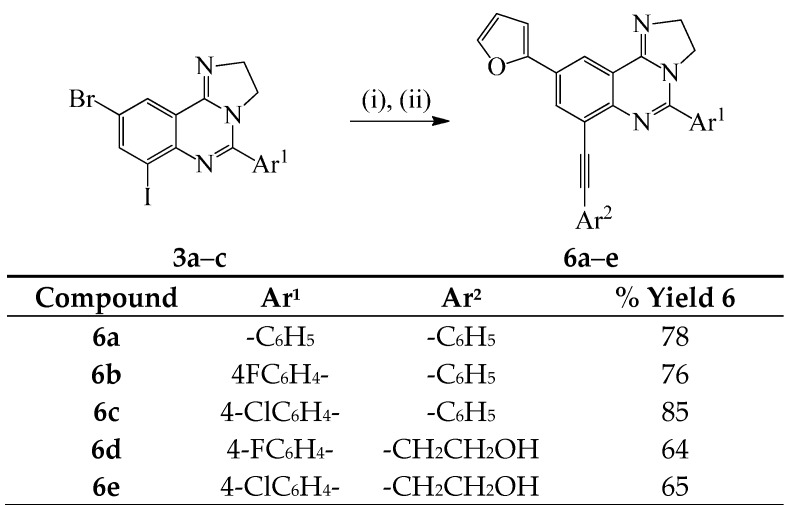
One-pot successive Sonogashira/Stille cross-coupling of **3a–c**. *Reagents and conditions*: (i) Ar^2^C≡CH, PdCl_2_(PPh_3_)_2_, CuI, K_2_CO_3_, 3:1 DMF–EtOH (*v*/*v*), r.t., 18 h; (ii) 2-(tributylstannyl)furan, DMF-ethanol, 100 °C, 2 h.

We also investigated the possibility to effect one-pot two step Sonogashira and Stille cross-coupling reactions on compounds **3a**–**c** as depicted in Scheme 4 below. Sonogashira cross-coupling of substrates **3a**–**c** was conducted with either phenyl acetylene or 3-butyn-1-ol (1.2 equiv.) at r.t. under the same conditions outlined in Scheme 2. After 18 h at r.t. (tlc monitoring), the reaction mixtures were each treated with 2-(tributylstannyl)furan (1.2 equiv.) in DMF-ethanol followed by heating at 100 °C. We isolated the corresponding unsymmetrically substituted polycarbo-substituted quinazolines **6a**–**e** in a single-pot operation.

### 2.2. In Vitro Cytotoxicity of Imidazo[1,2-c]quinazolines ***3***–***6***

Twenty two (22) of the imidazo[1,2-*c*]quinazolines were evaluated for their *in vitro* anticancer potential using the 3-(4,5-dimethylthiazole-2-yl)-2,5-diphenyltetrazoliumbromide based colorimetric cell viability (MTT) assay [13]. Their anticancer inhibitory activities were screened against the human breast adenocarcinoma (MCF-7) cells and human cervical cancer (HeLa) cell lines. The compounds were assayed at concentrations ranging from 0.1 to 100 μM with DMSO and doxorubicin hydrochloride as the negative and positive control, respectively. The LC_50_ values (lethal concentration at which 50% of the cells are killed) of compounds **3**–**6** (average from three independent experiments) against doxorubicin hydrochloride as a reference drug are represented in Table 1. (The percentage cell viability (±standard deviation) and linear regression plots (used to calculate LC_50_ values) for doxorubicin hydrochloride and compounds **3**–**6** are listed in the Supplementary Materials). Compounds **3b** and **3c** exhibit significant activity against the MCF-7 cells with LC_50_ values less than 1 µg/mL. Replacement of iodine with a phenylethynyl group resulted in significant cytotoxicity and selectivity against MCF-7 cells for the 5-phenyl-substituted derivative **4a**. The presence of the 7-phenylethynyl group, on the other hand, resulted in loss of activity for the 5-(4-halogenophenyl)-substituted derivatives **4b** and **4c**. The 7-(2-pyridylethynyl) derivative **4d** was found to exhibit activity against both MCF-7 and better than doxorubicin against HELa cell line. The presence of a substituent on the 5-aryl ring seems to lead to decreased cytotoxicity for both compounds **3** and **4**. Among the two series of polycarbo-substituted **5** and **6** imidazo[1,2-*c*]quinazolines only the 9-(4-fluorophenyl)–substituted derivatives **5b**, **5c** and **5h** and all the other derivatives were inactive against the two cell lines. Compounds **5b** and **5c** showed increased potency and selectivity against the human breast adenocarcinoma (MCF-7) cells as compared to doxorubicin hydrochloride. Compound **5h**, on the other hand, was found to be more cytotoxic to the HeLa cells compared to doxorubicin hydrochloride. The SAR based on these preliminary *in vitro* cytotoxicity results revealed that the 9-(4-fluorophenyl) moiety on the heterocyclic framework is important for biological property of these polycarbo-substituted imidazoquinazolines. The presence of a fluorine atom on the aromatic ring has been found to enhance the activity of the molecule due to its enhanced lipophilicity [14] due to compatible 2s and 2p orbital overlap of carbon and fluorine, which make the C*sp*^2^-F bond non-polarizable [15]. The C*sp*^2^–F bond is also known to exhibit strong polar interaction with the protein cavity [16].

**Table 1:** Cytotoxic effects of imidazo[1,2-*c*]quinazolines **3**–**6** against human breast adenocarcinoma (MCF-7) and human cervical cancer (HeLa) cell lines. 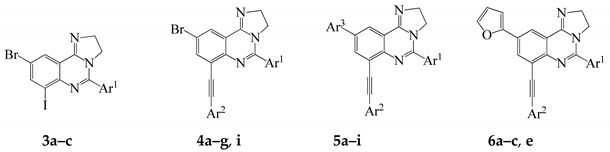

Compound	Ar^1^	Ar^2^	Ar^3^	LC_50_ (µM) ± SD	
				MCF-7	HeLa
**3a**	-C_6_H_5_	-	-	3.66 ± 0.12	13.7 ± 0.05
**3b**	4-FC_6_H_4_-	-	-	0.39 ± 0.05	9.73 ± 0.11
**3c**	4-ClC_6_H_4_-	-	-	0.78 ± 0.04	6.84 ± 0.08
**4a**	-C_6_H_5_	-C_6_H_5_	-	1.44 ± 0.13	18.1 ± 0.11
**4b**	4-FC_6_H_4_-	-C_6_H_5_	-	21.8 ± 0.12	7.96 ± 0.09
**4c**	4-ClC_6_H_4_-	-C_6_H_5_	-	13.8 ± 0.08	7.89 ± 0.11
**4d**	-C_6_H_5_	2-pyridyl	-	1.86 ± 0.09	0.23 ± 0.02
**4f**	4-FC_6_H_4_-	2-pyridyl	-	8.77 ± 0.04	2.29 ± 0.18
**4g**	4-ClC_6_H_4_-	2-pyridyl	-	3.21 ± 0.12	15.4 ± 0.26
**4i**	4-ClC_6_H_4_-	-(CH_2_)_2_OH	-	7.56 ± 0.08	20.7 ± 0.11
**5a**	-C_6_H_5_	-C_6_H_5_	4-FC_6_H_4_-	4.42 ± 0.07	1.75 ± 0.16
**5b**	4-FC_6_H_4_-	-C_6_H_5_	4-FC_6_H_4_-	˂0.1	0.75 ± 0.03
**5c**	4-ClC_6_H_4_-	-C_6_H_5_	4-FC_6_H_4_-	˂0.1	0.62 ± 0.09
**5e**	4-FC_6_H_4_-	-C_6_H_5_	4-MeOC_6_H_4_-	6.14 ± 0.15	0.69 ± 0.04
**5f**	-C_6_H_5_	2-pyridyl	4-FC_6_H_4_-	7.63 ± 0.06	1.99 ± 0.03
**5g**	4-FC_6_H_4_-	2-pyridyl	4-FC_6_H_4_-	12.4 ± 0.07	3.78 ± 0.12
**5h**	4-ClC_6_H_4_-	2-pyridyl	4-FC_6_H_4_-	0.34 ± 0.06	0.19 ± 0.01
**5i**	4-FC_6_H_4_-	-(CH_2_)_2_OH	4-FC_6_H_4_-	55.1 ± 0.03	23.6 ± 0.23
**6a**	-C_6_H_5_	-C_6_H_5_	-	4.23 ± 0.06	5.77 ± 0.16
**6b**	4-FC_6_H_4_-	-C_6_H_5_	-	73.6 ± 0.11	˃100
**6c**	4-ClC_6_H_4_-	-C_6_H_5_	-	˃100	0.42 ± 0.07
**6e**	4-ClC_6_H_4_-	-C_6_H_5_	-	8.63 ± 0.07	1.78 ± 0.06
**Doxorubicin hydrochloride**				0.32 ± 011	0.48 ± 0.01

## 3. Experimental Section

### 3.1. General Information

Melting points were recorded on a Thermocouple digital melting point apparatus and are uncorrected. IR spectra were recorded as powders using a Bruker VERTEX 70 FT-IR Spectrometer (Bruker Optics, Billerica, MA, USA) with a diamond ATR (attenuated total reflectance) accessory by using the thin-film method. For column chromatography, Merck Kieselgel 60 (0.063–0.200 mm) (Merck KGaA, Frankfurt, Germany) was used as stationary phase. NMR spectra were obtained as CDCl_3_ or DMSO-*d*_6_ solutions using an Agilent 500 MHz NMR spectrometer (Agilent Technologies, Oxford, UK) and the chemical shifts are quoted relative to the TMS peak. Low- and high-resolution mass spectra were recorded at an ionization potential of 70 eV using Synapt G2 Quadrupole Time-of-flight mass spectrometer (Waters Corp., Milford, MA, USA) at the University of Stellenbosch Mass Spectrometry Unit. The synthesis and characterization of compounds **1a**–**c** has been described before [10].

### 3.2. Typical Procedure for Preparation of the 2-(3-Aryl-6-bromo-8-iodoquinazolin-4-yl)ethanols ***2a***–***c***

A stirred mixture of **1a** (1.00 g, 2.24 mmol) and 3-aminoethanol (20 mL) was heated under reflux for 2 h. The mixture was allowed to cool to room temperature and then quenched slowly with an ice-cold water. The resulting precipitate was filtered and recrystallized from to afford **2**. The following products were prepared in this fashion: (The ^1^H- and ^13^C-NMR spectra of compounds **2**–**6** are listed in the Supplementary Materials).

*2-(6-Bromo-8-iodo-2-phenylquinazolin-4-yl)amino)ethanol* (**2a**). Solid (0.95 g, 90%), mp. 256–257 °C (toluene); ν_max_ (ATR) 743, 796, 846, 956, 1013, 1080, 1349, 1455, 1518, 1549, 1590, 3316 cm^−1^; ^1^H-NMR (500 MHz, DMSO-*d_6_*) δ_H_ 3.32 (1H, s, OH), 3.72 (2H, t, *J* = 5.5 Hz, CH_2_N-), 3.75 (2H, t, *J* = 5.5 Hz, -CH_2_O-), 4,82 (1H, t, *J* = 5.5 Hz, -NH), 7.52–7.54 (3H, m, 3′,4′,5′-H), 8.45 (1H, d, *J* = 1.5 Hz, 5-H), 8.53–8.58 (2H, m, 2′,6′-H), 8.61 (1H, d, *J* = 1.5 Hz, 8-H); ^13^C-NMR δ_C_ (125 MHz, DMSO-*d*_6_) 44.5, 59.4, 104.8, 115.5, 118.2, 126.3, 128.6, 128.8, 131.1, 138.3, 144.4, 148.7, 160.0, 160.8; MS *m*/*z* 470 (100, MH^+^); HRMS (ES): MH^+^, found 469.9356. C_16_H_14_N_3_O^79^BrI^+^ requires 469.9365.

*2-((6-Bromo-2-(4-fluorophenyl)-8-iodoquinazolin-4-yl)amino)ethanol* (**2b**). Solid (0.90 g, 85%), mp. 252–253 °C (toluene); ν_max_ (ATR) 706, 797, 859, 959, 1054, 1081, 1146, 1220, 1347, 1421, 1457, 1509, 1549, 1588, 3299 cm^−1^; δ_H_ (500 MHz, DMSO-*d*_6_) 3.31 (1H, s, -OH), 3.71 (2H, t, *J* = 5.5 Hz, -CH_2_N-), 3.74 (2H, t, *J* = 5.5 Hz, -CH_2_O-), 4,83 (1H, t, *J* = 5.5 Hz, -NH), 7.36 (2H, t, *J* = 9.0 Hz, 3′,5′-H), 8.45 (1H, d, *J* = 2.0 Hz, 5-H), 8.57 (2H, t, *J* = 9.0 Hz, 2′,6′-H), 8.60 (1H, d, *J* = 2.0 Hz, 7-H); ^13^C-NMR δ_C_ (125 MHz, DMSO-*d*_6_) 44.4, 59.4, 104.6, 115.4, 115.7 (d, ^1^*J*_CF_ = 21.8 Hz), 118.2, 126.3, 130.8 (d, ^3^*J*_CF_ = 8.6 Hz), 138.3 (d, ^4^*J*_CF_ = 2.9 Hz), 144.5, 148.7, 159.9, 160.0, 164.4 (d, ^2^*J*_CF_ 246.5 Hz); MS *m*/*z* 488 (100, MH^+^); HRMS (ES): MH^+^, found 488.9269. C_16_H_13_N_3_O^79^BrFI^+^ requires 488.9271.

*2-((6-Bromo-2-(4-chlorophenyl)-8-iodoquinazolin-4-yl)amino)ethanol* (**2c**). Solid (0.92 g, 87%), mp. 260–261 °C (toluene); ν_max_ (ATR) 797, 847, 859, 957, 1014, 1054, 1089, 1350, 1420, 1519, 1559, 1591, 3325 cm^−1^; δ_H_ (500 MHz, DMSO-*d*_6_) 3.31 (1H, s, -OH), 3,71 (2H, t, *J* = 5.5 Hz, -CH_2_N-), 3.71 (2H, t, *J* = 5.5 Hz, -CH_2_O-), 4,83 (1H, t, *J* = 5.5 Hz, -NH), 7.61 (2H, d, *J* = 8.5 Hz, 3′,5′-H), 8.46 (1H, d, *J* = 2.0 Hz, 5-H), 8.52 (2H, d, *J* = 8.5 Hz, 2′,6′-H), 8.62 (1H, d, *J* = 2.0 Hz, 7-H); ^13^C-NMR δ_C_ (125 MHz, DMSO-*d*_6_) 46.5, 59.4, 104.7, 115.5, 118.4, 126.3, 128.9, 130.2, 136.0, 137.2, 144.5, 148.6, 159.8, 160.0; MS *m*/*z* 504 (100, MH^+^); HRMS (ES): MH^+^, found 503.8979. C_16_H_13_N_3_O^35^Cl^79^BrI^+^ requires 503.8975.

### 3.3. Typical Procedure for Preparation of the 5-Aryl-9-bromo-7-iodo-2,3-dihydroimidazo[1,2-c]quinazolines ***3a***–***c***

*9-Bromo-7-iodo-5-phenyl-2,3-dihydroimidazo[1,2-c]quinazoline* (**3a**). A stirred mixture of **2a** (1.00 g, 2.13 mmol) and H_2_SO_4_ (30 mL) was heated at 120 °C for 2 h. The mixture was allowed to cool to room temperature and then added slowly to an ice-cold water (100 mL). The pH of the dilute acidic mixture was adjusted to 8–10 with 25% aqueous NaOH solution with stirring. The resultant precipitate was filtered and recrystallized to afford **3a** as a solid (0.87 g, 90%), mp. 241–242 °C (toluene); ν_max_ (ATR) 568, 692, 773, 879, 1004, 1312, 1441, 1581, 1659 cm^−1^; ^1^H-NMR δ_H_ (500 MHz, DMSO-*d*_6_) 3.40 (2H, t, *J* = 9.0 Hz, -CH_2_N-), 4,18 (2H, t, *J* = 9.0 Hz, -CH_2_N=), 7.53–7.61 (3H, m, 3′,4′,5′-H), 7.79 (2H, d, *J* = 8.5 Hz, 2′,6′-H), 8.05 (1H, d, *J* = 2.0 Hz, 5H), 8.35 (1H, d, *J* = 2.0 Hz, 7-H); ^13^C-NMR δ_C_ (125 MHz, DMSO-*d*_6_) 44.3, 47.7, 118.7, 123.0, 125.7, 126.8, 128.6, 128.9, 130.3, 135.3, 137.3, 141.5, 143.8, 157.5; MS *m*/*z* 452 (100, MH^+^); HRMS (ES): MH^+^, found 451.9359. C_16_H_12_N_3_^79^BrI^+^ requires 451.9359.

*9-Bromo-5-(4-fluorophenyl)-7-iodo-2,3-dihydroimidazo[1,2-c]quinazoline* (**3b**). Solid (0.85 g, 88%), mp. 253–254 °C (toluene); ν_max_ (ATR) 580, 705, 783, 860, 1228, 1356, 1405, 1563, 1649 cm^−1^; ^1^H-NMR δ_H_ (500 MHz, DMSO-*d*_6_) 4.02 (2H, t, *J* = 9.5 Hz, -CH_2_N-), 4.25 (2H, t, *J* = 9.5 Hz, CH_2_N=), 7.41 (2H, t, *J* = 8.5 Hz, 3′,5′-H), 7,88 (2H, t, *J* = 8.5 Hz, 2′,6′-H), 8.11 (1H, d, *J* = 2.0 Hz, 5-H), 8.40 (1H, d, *J* = 2.0 Hz, 7-H); ^13^C-NMR δ_C_ (125 MHz, DMSO-*d*_6_) 49.4, 52.2, 102.8, 116.1 (d, ^1^*J*_CF_ = 21.8 Hz), 117.9, 119.5, 127.8, 130.7 (d, ^4^*J*_CF_ = 2.6 Hz), 131.5 (d, ^3^*J*_CF_ = 8.6 Hz), 145.8, 146.4, 154.5, 162.9, 164.9 (d, ^1^*J*_CF_ = 247.5 Hz); MS *m*/*z* 470 (100, MH^+^); HRMS (ES): MH^+^, found 469.9161. C_16_H_11_N_3_^79^ BrIF^+^ requires 469.9165.

*9-Bromo-5-(4-chlorophenyl)-7-iodo-2,3-dihydroimidazo[1,2-c]quinazoline* (**3c**). Solid (0.88 g, 92%), mp. 224–225 °C (toluene); ν_max_ (ATR) 620, 782, 996, 1065, 1092, 1301, 1357, 1556, 1577, 1652 cm^−1^; ^1^H-NMR δ_H_ (500 MHz, DMSO-*d*_6_) 3.71 (2H, t, *J* = 9.5 Hz, CH_2_N-), 3.74 (2H, t, *J* = 9.5 Hz, CH_2_N=), 7.60 (2H, d, *J* = 8.5 Hz, 3′,5′-H), 8.46 (1H, d, *J* = 2.0 Hz, 5-H), 8.52 (2H, d, *J* = 8.5 Hz, 2′,6′-H), 8.62 (1H, d, *J* = 2.0 Hz, 7-H); ^13^C-NMR δ_C_ (125 MHz, DMSO-d6) 49.0, 54.4, 102.5, 118.9, 119.6, 127.6, 128.9, 130.6, 133.5, 135.9, 144.5, 146.2, 153.2, 154.3; MS *m*/*z* 485 (100, MH^+^); HRMS (ES): MH^+^, found 485.8881. C_16_H_11_N_3_^35^Cl^79^BrI^+^ requires 485.8670.

### 3.4. Typical Procedure for the Site-Selective Sonogashira Cross-Coupling of ***3a***–***c*** with Terminal Alkynes

*9-Bromo-5-phenyl-7-(phenylethynyl)-2,3-dihydroimidazo[1.2-c]quinazoline* (**4a**). A stirred mixture of **3a** (0.50 g, 1.106 mmol), PdCl_2_ (PPh_3_)_2_ (0.04 g, 0.06 mmol), CuI (0.02 g; 0.11 mmol) and K_2_CO_3_ (0.23 g, 1.66 mmol) in 3:1 DMF–EtOH (*v*/*v*, 15 mL) was purged with argon gas for 30 min. Phenylacetylene (0.12 g, 1.22 mmol) was added to the mixture using a syringe. The reaction mixture was stirred at room temperature for 18 h and then quenched with ice-cold water. The product was extracted into chloroform and the combined organic layers were washed with water, dried over Na_2_SO_4_, filtered and evaporated under reduced pressure. The residue was purified by column chromatography on silica gel to afford **4a** as a yellow solid (0.31 g, 66%), R_f_ (ethyl acetate) 0.47, mp. 212‒213 °C; ν_max_ (ATR) cm^−1^ 691, 753, 1015, 1090, 1283, 1455, 1538, 1642, 2870, 2923 cm^−1^; ^1^H-NMR δ_H_ (500 MHz, CDCl_3_) 4.01–4.18 (4H, m, -CH_2_CH_2_-), 7.31–7.34 (3H, m, 3′,4′,5′-H), 7.49–7.54 (5H, m, Ph), 7.76–7.78 (2H, m, 2′,6′-H), 7.88 (1H, d, *J* = 2.0 Hz, 8-H), 8.14 (1H, d, *J* = 2.0 Hz, 10-H); ^13^C-NMR δ_C_ (125 MHz, CDCl_3_) 49.2, 53.8, 85.6, 96.5, 118.9, 119.5, 123.0, 123.4, 127.8, 128.3, 128.8, 129.8, 131.8, 133.0, 136.9, 139.2, 146.3, 153.0, 154.4; 426; MS *m*/*z* (100, MH^+^); HRMS (ES): MH^+^ found 426.0604. C_24_H_17_N_3_^79^Br^+^ requires 426.0606.

*9-Bromo-5-(4-fluorophenyl)-7-(phenylethynyl)-2,3-dihydroimidazo[1.2-c]quinazoline* (**4b**). Solid (0.30 g, 64%), R_f_ (ethyl acetate) 0.5, mp. 194–195 °C; ν_max_ (ATR) 508, 559, 693, 713, 756, 836, 1158, 1237, 1377, 1423, 1510, 1604, 1642, 2870, 2922 cm^−1^; ^1^H-NMR δ_H_ (500 MHz, CDCl_3_) 4.10–4.16 (4H, m, -CH_2_CH_2_-), 7.19 (2H, t, *J* = 8.7 Hz, 3′,5′-H), 7.33–7.35 (3H, m, Ph), 7.52–7.54 (2H, m, Ph), 7.78 (2H, t, *J* = 8.7 Hz, 2′,6′-H), 7.88 (1H, d, *J* = 2.5 Hz, 8-H), 8.15 (1H, d, *J* = 2.5 Hz, 10-H); ^13^C-NMR δ_C_ (125 MHz, CDCl_3_) 49.3, 53.7, 85.6, 96.3, 115.6 (d, ^2^*J*_CF_ = 21.8 Hz), 118.8, 119.5, 123.0, 123.2, 127.8, 128.3, 128.6, 130.5 (d, ^3^*J*_CF_ = 8.5 Hz), 130.8 (d, ^4^*J*_CF_ = 3.0 Hz), 131.8, 139.2, 146.3, 153.0, 154.5, 164.0 (d, ^1^*J*_CF_ = 250 Hz); MS *m*/*z* 444 (100, MH^+^); HRMS (ES): MH^+^, found 444.0504. C_24_H_16_N_3_^79^BrF^+^ requires 444.0512.

*9-Bromo-5-(4-chlorophenyl)-7-(phenylethynyl)-2,3-dihydroimidazo[1,2-c]quinazoline* (**4c**). Solid (0.35 g, 77%), R_f_ (ethyl acetate) 0.47, mp. 170–171 °C; ν_max_ (ATR) 692, 753, 1015, 1072, 1377, 1538, 1574, 1642, 2869 cm^−1^; ^1^H-NMR δ_H_ (500 MHz, CDCl_3_) 4.14 (4H, s, -CH_2_CH_2_-), 7.33–7.35 (3H, m, Ph), 7.46 (2H, d, *J* = 8.5 Hz, 3′,5′-H), 7.52–7.54 (2H, m, Ph), 7.72 (2H, d, *J* = 8.5 Hz, 2′,6′-H), 7.88 (1H, d, *J* = 2.5 Hz, 8-H), 8.15 (1H, d, *J* = 2.5 Hz, 10-H); ^13^C-NMR δ_C_ (125 MHz, CDCl_3_) 49.2, 53.8, 85.5, 96.5, 118.9, 119.5, 123.0, 123.3, 127.8, 128.3, 128.7, 128.8, 129.8, 131.8, 133.0, 136.9, 139.2, 146.2, 152.9, 154.4; MS *m/z* 460 (100, MH^+^); HRMS (ES): MH^+^, found 460.0217. C_24_H_16_N_3_^35^Cl^79^Br^+^ requires 460.0217.

*9-Bromo-5-phenyl-7-(pyridin-2-ylethynyl)-2,3-dihydroimidazo[1,2-c]quinazoline* (**4d**). Solid (0.37 g, 79%), R_f_ (ethyl acetate) 0.23, mp. 163–164 °C; ν_max_ (ATR) 445, 525, 699, 774, 879, 1258, 1377, 1422, 1494, 1581, 1641, 1709, 2212, 2869 cm^−1^; ^1^H-NMR δ_H_ (500 MHz, CDCl_3_) 4.09 (4H, m, -CH_2_CH_2_-), 7.20 (1H, ddd, *J* = 1.0, 5.0 and 8.0 Hz, 4-H), 7.42–7.48 (4H, m, Ph), 7.62 (1H, dt, *J* = Hz, 5′′-H), 7.77 (2H, m, Ph), 7.96 (1H, d, *J* = 2.5 Hz, 8-H), 8.10 (1H, d, *J* = 2.5 Hz, 10-H), 8.61 (1H, d, *J* = 3.0 Hz, 3-H); ^13^C-NMR δ_C_ (125 MHz, CDCl_3_) 49.3, 53.3, 85.6, 99.9, 118.7, 119.5, 122.3, 123.0, 127.6, 128.3, 128.5, 128.7, 130.6, 134.5, 136.1, 140.0, 143.2, 146.9, 149.9, 154.4, 154.5; MS *m/z* 395 (100, MH^+^); HRMS (ES): MH^+^, found 394.0551. C_20_H_16_N_3_O^79^Br^+^ requires 394.0555.

*9-Bromo-5-(4-fluorophenyl)-7-(pyridin-2-ylethynyl)-2,3-dihydroimidazo[1,2-c]quinazoline* (**4e**). Solid (0.39 g, 83%), R_f_ (ethyl acetate) 0.21, mp. 201–202 °C; ν_max_ (ATR) 509, 549, 694, 777, 835, 1219, 1380, 1426, 1465, 1581, 1640, 1738, 2209, 2852 cm^−1^; ^1^H-NMR δ_H_ (500 MHz, CDCl_3_) 4.12 (4H, s, -CH_2_CH_2_-), 7.17 (2H, t, *J* = 8.7 Hz, 3,5-H), 7.22 (1H, ddd, *J* = 1.0, 5.0 and 8.0 Hz, 4-H), 7.50 (1H, d, *J* = 8.0 Hz, 6-H), 7.65 (1H, dt, *J* = 2.0 and 8.0 Hz, 5-H), 7.76 (2H, t, *J* = 8.7 Hz, 2,6-H), 7.95 (1H, d, *J* = 2.5 Hz, 8-H), 8.15 (1H, d, *J* = 2.5 Hz, 10-H), 8.61 (1H, d, *J* = 3.0 Hz, 3-H); ^13^C-NMR δ_C_ (125 MHz, CDCl_3_) 49.3, 53.3, 85.3, 95.0, 115.6 (d, ^2^*J*_CF_ = 21.9 Hz), 118.6, 119.6, 122.2, 122.9, 127.4, 128.5, 130.5 (d, ^2^*J*_CF_ = 8.5 Hz), 130.7 (d, ^4^*J*_CF_ = 3.1 Hz), 135.9, 143.2, 146.6, 150.0, 153.2, 154.2, 156.2, 163.9 (d, ^1^*J*_CF_ = 251.4 Hz); MS *m/z* 445 (100, MH^+^); HRMS (ES): MH^+^ found 445.0463. C_23_H_15_N_4_^79^BrF^+^ requires 445.0464. 

*9-Bromo-5-(4-chlorophenyl)-7-(pyridin-2-ylethynyl)-2,3-dihydroimidazo[1,2-c]quinazoline* (**4f**)*.* Solid (0.41 g, 85%), R_f_ (ethyl acetate) 0.26, mp. 214–215 °C; ν_max_ (ATR) 526, 560, 780, 1510, 1093, 1286, 1378, 1421, 1493, 1532, 1580, 1642, 2217, 2870 cm^−1^; ^1^H-NMR δ_H_ (500 MHz, CDCl_3_) 4.12 (4H, s, -CH_2_CH_2_-) 7.24 (1H, ddd, *J* = 1.0, 5.0 and 8.0 Hz, 4-H), 7.46 (2H, d, *J* = 7.8 Hz, 3,5-H), 7.52 (1H, d, *J* = 8.0 Hz, 6H), 7.66 (1H, dt, *J* = 2.0 and 8.0 Hz, 5H), 7.73 (2H, d, *J* = 7.8 Hz, 2,6-H), 7.95 (1H, d, *J* = 2.5, 8-H), 8.16 (1H, d, *J* = 2.5 Hz, 10-H), 8.63 (1H, d, *J* = 30 Hz, 3-H); ^13^C-NMR δ_C_ (125 MHz, CDCl_3_) 49.2, 53.8, 85.2, 95.0, 118.7, 119.6, 122.2, 122.9, 127.4, 128.5, 128.7, 129.7, 132.9, 136.0, 136.8, 139.7, 143.2, 146.6, 150.0, 153.2, 154.1; MS *m/z* 461 (100, MH^+^); HRMS (ES): MH^+^, found 461.0179. C_23_H_15_N_4_^35^Cl^79^Br^+^ requires 461.0169.

*4-(9-Bromo-5-phenyl-2,3-dihydroimidazo[1,2-c]quinazolin-7-yl)but-3-yn-1-ol* (**4g**). Solid (0.22 g, 55%), R_f_ (ethyl acetate) 0.21, mp. 155–157 °C; ν_max_ (ATR) 696, 716, 1065, 1282, 1318, 1378, 1552, 1638, 2872, 2920, 3163 cm^−1^; ^1^H-NMR δ_H_ (500 MHz, CDCl_3_) 1.70 (1H, br s, OH), 2.66 (2H, t, *J* = 5.5 Hz, -CH_2_C≡), 3.70 (2H, t, *J* = 5.5 Hz, -CH_2_O-), 4.03–4.09 (4H, m, -CH_2_CH_2_-), 7.48–7.53 (3H, m, 3′,4′,5′-H), 7.62–7.65 (2H, m, 2′,6′-H), 7.71 (1H, d, *J* = 2.5 Hz, 8-H), 8.10 (1H, d, *J* = 2.5 Hz, 10-H); ^13^C-NMR δ_C_ (125 MHz, CDCl_3_) 24.4, 49.2, 53.6, 60.6, 79.8, 95.4, 118.7, 119.5, 123.2, 127.5, 127.9, 128.4, 128.6, 130.7, 134.3, 138.0, 146.9, 154.6; MS *m/z* 395 (100, MH^+^); HRMS (ES): MH^+^, found 394.0551. C_20_H_17_N_3_O^79^Br^+^ requires 394.0555.

*4-(9-Bromo-5-(4-fluorophenyl)-2,3-dihydroimidazo[1,2-c]quinazolin-7-yl)but-3-yn-1-ol* (**4h**). Solid (0.26 g, 59%), R_f_ (ethyl acetate) 0.22, mp. 165–167 °C; ν_max_ (ATR) 541, 703, 783, 838, 890, 1065, 1162, 1234, 1378, 1423, 1511, 1608, 1639, 2872, 2922, 3166 cm^−1^; ^1^H-NMR δ_H_ (500 MHz, CDCl_3_) 1.50 (1H, s, -OH), 2.68 (2H, t, *J* = 5.5 Hz, -CH_2_C≡), 3.72 (2H, t, *J* = 5.5 Hz, -CH_2_-O), 4.04–4.13 (4H, m, -CH_2_CH_2_-), 7.19 (2H, t, *J* = 8.5 Hz, 3′,5′-H), 7.66 (2H, t, *J* = 8.5 Hz, 2′,6′-H), 7.70 (1H, d, *J* = 2.5 Hz, 8-H), 8.14 (1H, d, *J* = 2.5 Hz, 10-H); ^13^C-NMR δ_C_ (125 MHz, CDCl_3_) 24.5, 49.2, 53.7, 60.5, 92.2, 115.8 (d, ^2^*J*_CF_ = 21.9 Hz), 118.8, 123.1, 127.5, 128.5, 130.2 (d, ^3^*J*_CF_ = 8.6 Hz), 131.9, 132.0 (d, ^4^*J*_CF_ = 2.5 Hz), 132.1, 138.1, 146.8, 153.8, 162.9 (d, ^1^*J*_CF_ = 246.7 Hz); MS *m*/*z* 412 (100, MH^+^); HRMS (ES): MH^+^, found 412.0451. C_20_H_16_N_3_O^79^BrF^+^ requires 412.0461.

*4-(9-Bromo-5-(4-chlorophenyl)-2,3-dihydroimidazo[1,2-c]quinazolin-7-yl)but-3-yn-1-ol* (**4i**). Solid (0.30 g, 68%), R_f_ (ethyl acetate) 0.22, mp. 176–178 °C; ν_max_ (ATR) 690, 783, 824, 1066, 1092, 1283, 1457, 1640, 2872, 2921, 3173 cm^−1^; ^1^H-NMR δ_H_ (500 MHz, CDCl_3_) 2.67 (2H, t, *J* = 5.5 Hz, -CH_2_C≡), 3.10 (1H, br s, -OH), 3.70 (2H, t, *J* = 5.5 Hz, -CH_2_O-), 4.01–4.12 (4H, m, -CH_2_CH_2_-), 7.46 (2H, d, *J* = 8.7 Hz, 3′,4′,5′-H), 7.59 (2H, d, *J* = 8.7 Hz, 2′,6′-H), 7.71 (1H, d, *J* = 2.5 Hz, 8-H), 8.09 (1H, d, *J* = 2.5 Hz, 10-H); ^13^C-NMR δ_C_ (125 MHz, CDCl_3_) 24.3, 49.2, 53.6, 60.5, 79.7, 95.3, 119.0, 119.4, 123.1, 127.4, 128.9, 129.4, 132.6, 137.0, 138.0, 146.6, 153.6, 154.0; MS *m*/*z* 428 (100, MH^+^); HRMS (ES): MH^+^, found 428.0157. C_20_H_16_N_3_O^35^Cl^79^Br^+^ requires 428.0166.

### 3.5. Typical Procedure for the Suzuki-Miyaura Cross-Coupling of ***4a***–***h***

*9-(4-Fluorophenyl)-5-phenyl-7-(phenylethynyl)*-*2,3-dihydroimidazo[1,2-c]quinazoline* (**5a**). A stirred mixture of **4a** (0.30 g, 0.70 mmol), PdCl_2_(PPh_3_)_2_ (0.025 g, 0.035 mmol), PCy_3_ (0.02 g, 0.07 mmol) and K_2_CO_3_ (0.15 g, 1.06 mmol) in 3:1 DMF–EtOH (*v*/*v*, 15 mL) was purged with argon gas for 30 min. 4-Fluorophenylboronic acid (0.12 g, 0.84 mmol) was added to the mixture using a syringe. The reaction mixture was heated at 100 °C for 2 h and then quenched with an ice-cold water. The product was extracted into chloroform and the combined organic layers were washed with water, dried over Na_2_SO_4_, filtered and evaporated under reduced pressure. The residue was purified by column chromatography on silica gel to afford **5a** as a yellow solid (0.23 g, 74%), R_f_ (ethyl acetate) 0.16, mp. 306–307 °C; ν_max_ (ATR) 693, 759, 834, 1225, 1353, 1512, 1538, 1637, 2872, 2964 cm^−1^; ^1^H-NMR δ_H_ (500 MHz, CDCl_3_) 4.12–4.19 (4H, m, -CH_2_CH_2_-), 7.15 (2H, t, *J* = 8.5 Hz, 3′,5′-H), 7.32–7.34 (3H, m, Ph), 7.50–7.56 (3H, m, Ph), 7.56–7.58 (2H, m, Ph), 7.67 (2H, t, *J* = 8.5 Hz, 2′,6′-H), 7.79–7.82 (2H, m, Ph), 8.01 (1H, d, *J* = 2.0 Hz, 8-H), 8.20 (1H, d, *J* = 2.0 Hz, 10-H); ^13^C-NMR δ_C_ (125 MHz, CDCl_3_) 49.3, 53.7, 87.0, 95.2, 115.8 (d, ^2^*J*_CF_ 21.8 Hz), 118.7, 121.9, 123.1, 123.5, 128.2, 128.3, 128.4 (3xC), 128.6 (d, ^3^*J*_CF_ 8.5 Hz), 130.4, 131.8, 134.9 (d, ^4^*J*_CF_ 2.8 Hz), 135.3, 137.7, 146.7, 153.7, 155.7, 162.8 (d, ^1^*J*_CF_ 245.6 Hz); MS *m*/*z* 442; HRMS (ES): MH^+^, found 442.1711. C_30_H_21_N_3_F^+^ requires 442.1720.

*5,9-Bis(4-fluorophenyl)-7-(phenylethynyl)*-*2,3-dihydroimidazo[1,2-c]quinazoline* (**5b**). Solid (0.22 g, 71%), R_f_ (ethyl acetate) 0.17, mp. 298–299 °C; ν_max_ (ATR) 576, 697, 760, 851, 1156, 1237, 1509, 1604, 1636, 2872, 2962 cm^−1^; ^1^H-NMR δ_H_ (500 MHz, CDCl_3_) 4.16 (4H, s, -CH_2_CH_2_-), 7.14 (2H, t, *J* 8.7 Hz, 3′,5′-H), 7.19 (2H, t, *J* 8.7 Hz, 3′′,5′′-H), 7.33–7.35 (3H, m, Ph), 7.55–7.57 (2H, m, Ph), 7.65 (2H, t, *J* = 8.7 Hz, 2′,6′-H), 8.82 (2H, t, *J* = 8.7 Hz, 2′′,6′′-H), 8.00 (1H, d, *J* = 2.0 Hz, 8-H), 8.17 (1H, d, *J* = 2.0 Hz, 10-H); ^13^C-NMR δ_C_ (125 MHz, CDCl_3_) 49.3, 53.8, 86.9, 95.3, 115.6 (d, ^2^*J*_CF_ = 21.7 Hz), 115.8 (d, ^2^*J*_CF_ = 21.0 Hz), 118.6, 121.9, 123.1, 123.4, 128.3, 128.4, 128.6 (d, ^3^*J*_CF_ = 8.6 Hz), 130.7 (d, ^3^*J*_CF_ = 8.5 Hz), 131.1 (d, ^4^*J*_CF_ = 2.8 Hz), 131.8, 135.2 (d, ^4^*J*_CF_ = 3.0 Hz), 135.3, 137.8, 146.5, 152.8, 155.6, 162.8 (d, ^1^*J*_CF_ = 247.6 Hz), 163.8 (d, ^1^*J*_CF_ = 247.6 Hz); MS *m*/*z* 460; HRMS (ES): MH^+^, found 460.1622. C_30_H_20_N_3_F_2_^+^ requires 460.1625.

*5-(4-Chlorophenyl)-9-(4-fluorophenyl)-7-(phenylethynyl)*-*2,3-dihydroimidazo[1,2-c]quinazoline* (**5c**). Solid (0.25 g, 81%), R_f_ (ethyl acetate) 0.19, mp. 285–286 °C; ν_max_ (ATR) 520, 543, 691, 780, 970, 1090, 1377, 1492, 1537, 1641, 2869, 2927 cm^−1^; ^1^H-NMR δ_H_ (500 MHz, CDCl_3_) 4.15 (4H, s, -CH_2_CH_2_), 7.13 (2H, t, *J* = 8.5 Hz, 3′,5′-H), 7.30–7.40 (3H, m, Ph), 7.47 (2H, d, *J* = 8.5 Hz, 3′′,5′′-H), 7.55–7.571 (2H, m, Ph), 7.64 (2H, t, *J* = 8.5 Hz, 2′,6′-H), 7.76 (2H, d, *J* = 8.5 Hz, 2′′,6′′-H), 8.00 (1H, d, *J* = 2.0 Hz, 8-H), 8.16 (1H, d, *J* = 2.0 Hz, 10-H); ^13^C-NMR δ_C_ (125 MHz, CDCl_3_) 49.3, 53.8, 86.9, 95.3, 115.8 (d, ^2^*J*_CF_ = 21.9 Hz), 118.6, 121.9, 123.1, 123.3, 128.3, 128.4, 128.6 (d, ^3^*J*_CF_ = 7.6 Hz), 128.7, 131.8, 133.3, 135.1 (d, ^4^*J*_CF_ = 3.9 Hz), 135.3, 136.7, 137.9, 152.6, 155.5, 162.8 (d, ^1^*J*_CF_ = 246.5 Hz); MS *m*/*z* 476; HRMS (ES): MH^+^, found 476.1332. C_30_H_20_N_3_F^35^Cl^+^ requires 476.1330.

*2,3-Dihydro-9-(4-methoxyphenyl)-5-phenyl-7-(2-phenylethynyl)imidazo[1,2-c]quinazoline* (**5d**). Solid (0.28 g, 88%), R_f_ (ethyl acetate) 0.20, mp. 275–276 °C; ν_max_ (ATR) 530, 692, 759, 828, 1012, 1180, 1243, 1355, 1432, 1492, 1515, 1540, 1634; ^1^H-NMR δ_H_ (500 MHz, CDCl_3_) 3.87 (3H, s, -OCH_3_), 4.13–4.20 (4H, m, -CH_2_CH_2_-), 7.00 (2H, d, *J* = 8.7 Hz, 3′′,5′′-H), 7.30–7.34 (3H, m, ArH), 7.57–7.59 (2H, m, ArH), 7.50–7.52 (3H, m, ArH), 7.65 (2H, d, *J* = 8.7 Hz, 2′′,6′′-H), 7.80–7.82 (2H, m, ArH), 8.03 (1H, d, *J* = 1.0 Hz, 8-H), 8.22 (1H, d, *J* = 1.0 Hz, 10-H); ^13^C-NMR δ_C_ (125 MHz, CDCl_3_) 49.3, 53.7, 55.4, 87.3, 95.0, 114.3, 118.6, 121.7, 122.6, 123.6, 128.0, 128.2, 128.3, 128.4, 128.5, 130.4, 131.5, 131.8, 135.1, 138.4, 146.2, 153.4, 159.6; MS *m*/*z* 454 (100, MH^+^); HRMS (ES): MH^+^, found 454.1918. C_31_H_24_N_3_O^+^ requires 454.1919.

*4-(5-Fluorophenyl)-2,3-dihydro-9-(4-methoxyphenyl)-7-(2-phenylethynyl)imidazo[1,2-c]quinazoline* (**5e**). Solid (0.26 g, 81%), R_f_ (ethyl acetate) 0.18, mp. 292–293 °C; ν_max_ (ATR) 524, 697, 758, 831, 1014, 1113, 1178, 1242, 1285, 1355, 1461, 1513, 1538, 1604, 1635; ^1^H-NMR δ_H_ (500 MHz, CDCl_3_) 3.86 (3H, s, -OCH_3_), 4.16 (4H, s, -CH_2_CH_2_-), 6.59 (2H, d, *J* = 8.7 Hz, 3′′,5′′-H), 7.18 (2H, t, *J* = 8.7 Hz, 3′,5′-H), 7.32–7.34 (3H, m, Ph), 7.54–7.56 (2H, m, Ph), 7.63 (2H, d, *J* = 8.7 Hz, 2′′,6′′-H), 7.81 (2H, t, *J* = 8.7 Hz, 2′,6′-H), 8.02 (1H, d, *J* = 1.0 Hz, 8-H), 8.21 (1H, d, *J* = 1.0 Hz, 10-H); ^13^C-NMR δ_C_ (125 MHz, CDCl3) 49.4, 53.7, 55.4, 87.1, 95.0, 114.3, 115.6 (d, ^2^*J*_CF_ = 22.8 Hz), 121.7, 122.7, 123.5, 128.0, 128.2, 128.3, 129.7, 130.7 (d, ^3^*J*_CF_ = 8.6 Hz), 131.5 (d, ^4^*J*_CF_ = 3.0 Hz), 131.8, 135.1, 138.5, 146.1, 152.4, 159.6, 164.9 (d, ^1^*J*_CF_ = 250.2 Hz); MS *m*/*z* 472 (100, MH^+^); HRMS (ES): MH^+^, found 472.1819. C_31_H_23_N_3_OF^+^ requires 472.1825.

*9-(4-Fluorophenyl)-2,3-dihydro-5-phenyl-7-(2-pyridin-2-yl)ethynylimidazo[1,2-c]quinazoline* (**5f**). Solid (0.19 g, 72%), R_f_ (ethyl acetate) 0.25, mp. 254–255 °C; ν_max_ (ATR) 550, 695, 778, 837, 1161, 1234, 1353, 1426, 1466, 1533, 1581, 1640, 1739, 2208, 2869 cm^−1^; ^1^H-NMR δ_H_ (500 MHz, CDCl_3_) 4.12–4.16 (4H, m, -CH_2_CH_2_-), 7.14 (2H, t, *J* = 8.7 Hz, 3′,5′-H), 7.22 (1H, ddd, *J* = 1.0, 5.0 and 8.0 Hz, 4-H), 7.49–7.52 (3H, m, ArH), 7.55 (1H, d, *J* = 8.0 Hz, 6-H), 7.63–7.65 (2H, m, ArH), 7.66 (1H, dt, *J* = 2.0 and 8.0 Hz, 5-H), 8.87 (2H, t, *J* = 8.5 Hz, 2′,6′-H), 8.09 (1H, d, *J* = 2.0 Hz, 8-H), 8.21 (1H, d, *J* = 2.0 Hz, 10-H), 8.63 (1H, d, *J* = 5.0, 3-H); ^13^C-NMR δ_C_ (125 MHz, CDCl_3_) 49.3, 53.7, 86.9, 94.1, 115.8 (d, ^2^*J*_CF_ = 21.9 Hz), 118.7, 120.9, 122.7, 123.8, 127.5, 128.4, 128.5, 128.6 (d, ^3^*J*_CF_ = 8.5 Hz), 130.5, 134.8, 135.0 (d, ^4^*J*_CF_ = 3.9 Hz), 135.9, 136.0, 137.7, 143.6, 147.0, 150.0, 154.0, 155.5, 162.8 (d, ^1^*J*_CF_ = 245.6 Hz); MS *m*/*z* 443 (100, MH^+^); HRMS (ES): MH^+^, found 443,1670. C_29_H_20_N_4_F^+^ requires 443.1672.

*5,9-Bis(4-fluorophenyl)-2,3-dihydro-7-(2-pyridin-2-yl)ethynylimidazo[1,2-c]quinazoline* (**5g**). Solid (0.19 g, 72%), R_f_ (ethyl acetate) 0.20, mp. 263–264 °C; ν_max_ (ATR) 777, 836, 1178, 1195, 1384, 1467, 1510, 1606, 1639, 1739, 2209, 2869 cm^−1^; ^1^H-NMR δ_H_ (500 MHz, CDCl_3_) 4.15 (4H, s, -CH_2_CH_2_-), 7.13 (2H, t, *J* = 8.5 Hz, 3,5-H), 7.17 (2H, t, *J* = 8.5 Hz, 3′,5′-H), 7.24 (1H, ddd, *J* = 1.0, 5.0 and 8.0 Hz, 4-H), 7.54 (1H, d, *J* = 8.0 Hz, 6-H), 7.63 (2H, t, *J* = 8.5 Hz, 2,6-H), 7.66 (1H, dt, *J* = 2.0 and 8.0 Hz, 5-H), 7.81 (2H, t, *J* = 8.5 Hz, 2′,6′-H), 8.09 (1H, d, *J* = 2.5 Hz, 8-H), 8.21 (1H, d, *J* = 2.5 Hz, 10-H), 8.63 (1H, d, *J* = 5.0 Hz, 3-H); ^13^C-NMR δ_C_ (125 MHz, CDCl_3_) 49.3, 53.8, 86.8, 94.1, 115.5 (d, ^2^*J*_CF_ = 21.9 Hz), 115.8 (d, ^2^*J*_CF_ = 21.9 Hz), 118.6, 120.9, 122.8, 123.8, 127.5, 128.5, 128.6 (d, ^3^*J*_CF_ = 8.5 Hz), 130.7 (d, ^3^*J*_CF_ = 8.5 Hz), 131.0 (d, ^4^*J*_CF_ = 3.9 Hz), 135.7 (d, ^4^*J*_CF_ = 3.9 Hz), 135.9, 136.0, 137.7, 143.6, 146.9, 150.1, 153.0, 162.8 (d, ^1^*J*_CF_ = 245.6 Hz), 163.9 (d, ^1^*J*_CF_ = 250.2 Hz); MS *m*/*z* 461(100, MH^+^); HRMS (ES): MH^+^, found 461.1572. C_29_H_19_N_4_F_2_^+^ requires 461.1578.

*5-(4-Chlorophenyl)-9-(4-fluorophenyl)-2,3-dihydro-7-(2-pyridin-2-yl)ethynylimidazo[1,2-c]quinazoline* (**5h**). Solid (0.22 g, 85%), R_f_ (ethyl acetate) 0.30, mp. 251–252 °C; ν_max_ (ATR) 510, 777, 835, 1015, 1091, 1156, 1221, 1514, 1640, 1738, 2213, 2876 cm^−1^; ^1^H-NMR δ_H_ (500 MHz, CDCl_3_) 4.14 (4H, s, -CH_2_CH_2_-), 7.13 (2H, t, *J* = 8.5 Hz, 3,5-H), 7.23 (1H, dt, *J* = 1.0 and 5.0 Hz, 4-H), 7.46 (2H, d, *J* = 8.5 Hz, 3',5'-H), 7.54 (1H, d, *J* = 8.0 Hz, 6-H), 7.60 (2H, t, *J* = 8.5 Hz, 2,6-H), 7.67 (1H, dt, *J* = 2.0 and 7.5 Hz, 5-H), 7.75 (2H, d, *J* = 8.5 Hz, 2,6-H), 8.08 (1H, d, *J* = 2.0 Hz, 8-H), 8.19 (1H, d, *J* = 2.0 Hz, 10-H), 8.63 (1H, d, *J* = 4.5 Hz, 3-H); ^13^C-NMR δ_C_ (125 MHz, CDCl_3_) 49.2, 53.7, 86.8, 94.1, 115.8 (d, ^2^*J*_CF_ = 21.8 Hz), 120.9, 122.8, 123.9, 127.4, 128.4, 128.5 (d, ^3^*J*_CF_ = 8.6 Hz), 128.6, 128.7, 128.8, 129.7, 129.8, 129.9, 134.9 (d, ^4^*J*_CF_ = 3.7 Hz), 136.0, 136.1, 137.8, 143.5, 146.8, 150.1, 162. (d, ^1^*J*_CF_ = 246.6 Hz); MS *m*/*z* 488 (100, MH^+^); HRMS (ES): MH^+^, found 488.1535. C_29_H_19_N_4_F^35^Cl^+^ requires 488.1530.

*4-(5-(4-Chlorophenyl)-9-(4-fluorophenyl)-2,3-dihydroimidazo[1,2-c]quinazolin-7-yl)but-3-yn-1-ol* (**5i**). Solid (0.22 g, 85%), R_f_ (ethyl acetate) 0.30, mp. 251–252 °C; ν_max_ (ATR) 537, 777, 835, 1067, 1233, 1355, 1516, 1639, 1738, 2209, 2875 cm^−1^; ^1^H-NMR δ_H_ (500 MHz, CDCl_3_) 2.70 (2H, t, *J* = 6.0 Hz, ≡CCH_2_-), 3.18 (1H, br s, OH), 3.34 (2H, t, *J* = 6.0 Hz, -CH_2_OH), 4.04–4.06 (2H, m, =NCH_2_-), 4.11–4.15 (2H, m, -CH_2_N) 7.13 (2H, t, *J* = 8.5 Hz, 3,5-H), 7.49 (2H, d, *J* = 8.7 Hz, 3′,5′-H), 7.61 (2H, d, *J* = 8.5 Hz, 2,6-H), 7.62 (2H, d, *J* = 8.7 Hz, 2′,6′-H), 7.84 (1H, d, *J* = 2.5 Hz, 8-H), 8.16 (1H, d, *J* = 2.5 Hz, 10-H); ^13^C-NMR δ_C_ (125 MHz, CDCl_3_) 24.4, 49.2, 53.7, 60.7, 81.1, 94.1, 115.8 (d, ^2^*J*_CF_ = 21.9 Hz), 118,5, 121.8, 122.8, 128.6 (d, ^3^*J*_CF_ = 8.5 Hz), 128.9, 129.5, 132.8, 133.9, 135.1 (d, ^4^*J*_CF_ = 3.9 Hz), 136.8, 138.0, 146.9, 153.3, 162.8 (d, ^1^*J*_CF_ = 245.6 Hz); MS *m*/*z* 444 (100, MH^+^); HRMS (ES): MH^+^, found 444.1272. C_26_H_20_N_3_O^35^ClF^+^ requires 444.1279.

### 3.6. Typical Procedure for the One-Pot Sonogashira and Stille Cross-Coupling of ***3a***–***c***

*9-(Furan-2-yl)-2,3-dihydro-5-phenyl-7-(2-phenylethynyl)imidazo*[1.2-c]*quinazoline* (**6a**). A stirred mixture of **3a** (0.5 g, 1.11 mmol), PdCl_2_(PPh_3_)_2_ (0.04 g, 0.06 mmol), CuI (0.02 g; 0.11 mmol) and K_2_CO_3_ (0.23 g, 1.66 mmol) in 3:1 DMF–EtOH (*v*/*v*, 15 mL) was purged with argon gas for 30 min. Phenyl acetylene (0.12 g, 1.22 mmol) was added to the mixture using a syringe. The reaction mixture was stirred at room temperature for 18 h and then a solution of 2-(tributylstannyl)furan (0.59 g, 1.6 mmol) in 3:1 DMF–EtOH (5 mL) was added via a syringe. The mixture was heated at 100 °C for 2 h and then quenched with an ice-cold water. The product was extracted into chloroform and the combined organic layers were washed with water, dried over Na_2_SO_4_, filtered and evaporated under reduced pressure. The residue was purified by column chromatography on silica gel to afford **6a** as a yellow solid (0.36 g, 78%), R_f_ (ethyl acetate) 0.30, mp. 206‒207 °C; ν_max_ (ATR) 695, 756, 781, 1013, 1089, 1211, 1250, 1351, 1403, 1491, 1532, 1638, 2869 cm^−1^; ^1^H-NMR δ_H_ (500 MHz, CDCl_3_) 4.11–4.30 (4H, m, -CH_2_CH_2_-), 6.50 (1H. dd, *J* = 1.5 and 3.0 Hz, 4-H), 6.78 (1H, d, *J* = 3.0 Hz, 5-H), 7.32–7.34 (3H, m, ArH), 7.74–7.51 (4H, m, 3-H and ArH), 7.55–7.58 (2H, m, ArH), 7.78–7.80 (2H, m, ArH), 8.13 (1H, d, *J* = 2.0 Hz, 8-H), 8.28 (1H, d, *J* = 10-H); ^13^C-NMR δ_C_ (125 MHz, CDCl_3_) 49.3, 53.6, 86.9, 95.2, 106.2, 112.0, 118.6, 119.9, 121.8, 123.5, 128.2, 128.3, 128.4, 128.6, 130.4, 131.8, 132.1, 134.9, 142.6, 146.5, 152.4, 153.4, 155.6; MS *m*/*z* 416 (100, MH^+^); HRMS (ES): MH^+^, found 416.1748. C_28_H_20_N_3_O^+^ requires 416.1763.

*5-(4-Fluorophenyl)-9-(furan-2-yl)-2,3-dihydro-7-(2-phenylethynyl)imidazo[1,2-c]quinazoline* (**6b**). Solid (0.35 g, 76%), R_f_ (ethyl acetate) 0.27, mp. 225‒227 °C; ν_max_ (ATR) 693, 716, 734, 755, 1012, 1252, 1385, 1511, 1604, 1641, 2868 cm^−1^; ^1^H-NMR δ_H_ (500 MHz, CDCl_3_) 4.15 (4H, s, -CH_2_CH_2_-), 6.50 (1H, dd, *J* = 2.0 and 3.5 Hz, 4-H), 6.77 (1H, d, *J* = 3.5 Hz, 5-H), 7.18 (2H, t, *J* = 8.7 Hz, 3′,5′-H), 7.33–7.36 (3H, m, ArH), 7.49 (1H, d, *J* =2.0 Hz, 3-H), 7.55–7.57 (2H, m, ArH), 7.81 (2H, t, *J* = 8.7 Hz, 2′,6′-H), 8.12 (1H, d, *J* = 2.0 Hz, 8-H), 8.26 (1H, d, *J* = 2.0 Hz, 10-H); ^13^C-NMR δ_C_ (125 MHz, CDCl_3_) 49.3, 53.6, 86.8, 95.2, 106.2, 111.9, 115.5 (d, ^2^*J*_CF_ = 21.8 Hz), 118.4, 119.9, 121.7, 123.4, 128.2, 128.3, 128.7, 130.5 (d, ^3^*J*_CF_ = 8.5 Hz), 130.1 (d, ^4^*J*_CF_ = 3.0 Hz), 131.7, 132.0, 142.6, 146.2, 152.2, 152.4, 155.5, 163.9 (d, ^1^*J*_CF_ = 250.2 Hz); MS *m*/*z* 432 (100, MH^+^); HRMS (ES): MH^+^, found 432.1516. C_28_H_19_N_3_OF^+^ requires 432.1512.

*5-(4-Chlorophenyl)-9-(furan-2-yl)-2,3-dihydro-7-(2-phenylethynyl)imidazo[1,2-c]quinazoline* (**6c**). Solid (0.39 g, 85%), R_f_ (ethyl acetate) 0.28, mp. 222‒223 °C; ν_max_ (ATR) 691, 755, 792, 884, 1011, 1386, 1539, 1639, 2866 cm^−1^; ^1^H-NMR δ_H_ (500 MHz, CDCl_3_) 4.15 (4H, s, -CH_2_CH_2_-), 6.50 (1H, dd, *J* = 2.0 and 3.5 Hz, 4-H), 6.78 (1H, d, *J* = 3.5 Hz, 5-H), 7.33–7.35 (3H, m, ArH), 7.47 (2H, d, *J* = 8.5 Hz, 3′,5′-H), 7.49 (1H, d, *J* = 2.0 Hz, 3-H), 7.55–7.57 (2H, m, ArH), 7.74 (2H, t, *J* = 8.5 Hz, 2′,6′-H), 8.11 (1H, d, *J* = 2.0 Hz, 8-H), 8.26 (1H, d, *J* = 2.0 Hz, 10-H); ^13^C-NMR δ_C_ (125 MHz, CDCl_3_) 49.2, 53.7, 86.8, 95.3, 106.4, 112.0, 118.6, 119.9, 121.8, 123.5, 128.2, 128.3, 128.7, 128.8, 129.8, 131.8, 132.1, 132.3, 136.7, 142.7, 146.2, 152.3, 152.4, 155.5; MS *m*/*z* 448 (100, MH^+^); HRMS (ES): MH^+^, found 448.1223. C_28_H_19_N_3_O^35^Cl^+^ requires 448.1217.

*4-(5-(4-Fluorophenyl)-9-furan-2-yl)-2,3-dihydroimidazo[1,2-c]quinazolin-7-yl)but-3-yn-1-ol* (**6d**). Solid (0.27 g, 64%), R_f_ (ethyl acetate) 0.30, mp. 173–174 °C; ν_max_ (ATR) 592, 727, 839, 855, 886, 1015, 1065, 1227, 1514, 1608, 1640, 2875 cm^−1^; ^1^H-NMR δ_H_ (500 MHz, CDCl_3_) 2.69 (2H, t, *J* = 6.0 Hz, -CH_2_C≡), 3.18 (1H, br s, OH), 3.73 (2H, t, *J* = 6.0 Hz, -CH_2_O), 4.04–4.09 (2H, m, -CH_2_N), 4.11–4.15 (2H, m, -CH_2_N=), 7.13 (2H, t, *J* = 8.7 Hz, 3,5-H), 7.48 (2H, d, *J* = 8.5 Hz, 3′,5′-H), 7.61 (2H, t, *J* = 8.5 Hz, 2,6-H), 7.62 (2H, d, *J* = 8.7 Hz, 2′,6′-H), 7.84 (1H, d, *J* = 2.5 Hz, 8-H), 8.16 (1H, d, *J* = 2.5 Hz, 10-H); ^13^C-NMR δ_C_ (125 MHz, CDCl_3_) 24.4, 49.2, 53.7, 60.7, 81.1, 94.1, 115.8 (d, ^2^*J*_CF_ = 21.9 Hz), 118.5, 121.7, 122.7, 128.6 (d, ^3^*J*_CF_ 8.5 Hz), 128.9, 132.8, 133.9, 135.9 (d, ^4^*J*_CF_ 3.8 Hz), 136.8, 138.0, 153.3, 162.8 (d, ^1^*J*_CF_ 246.6 Hz); MS *m*/*z* 432 (100, MH^+^); HRMS (ES): MH^+^, found 432.1516. C_20_H_19_N_3_OF^+^ requires 432.1512.

*4-(5-(4-Chlorophenyl)-9-furan-2-yl)-2,3-dihydroimidazo[1,2-c]quinazolin-7-yl)but-3-yn-1-ol* (**6e**). Solid (0.28 g, 65%), R_f_ (ethyl acetate) 0.31, mp. 151–153 °C; ν_max_ (ATR) 729, 784, 885, 1014, 1063, 1091, 1267, 1494, 1545, 1600, 2875 cm^−1^; ^1^H-NMR δ_H_ (500 MHz, CDCl_3_) 2.69 (2H, t, *J* = 6.0 Hz, CH_2_C≡), 3.18 (1H, br s, OH), 3.73 (2H, t, *J* = 6.0 Hz, -CH_2_O), 4.03–4.30 (4H, m, -CH_2_CH_2_-), 6.49 (1H, dd, *J* = 2.0 and 3.0 Hz, 4-H), 6.75 (1H, d, *J* = 3.5 Hz, 5-H), 7.48 (2H, d, *J* = 8.5 Hz, 3′,5′-H), 7.47 (1H, d, *J* = 2.0 Hz, 3-H), 7.62 (2H, d, *J* = 8.5 Hz, 2′,6′-H), 7.95 (1H, d, *J* = 2.0 Hz, 8-H), 8.23 (1H, d, *J* = 2.0 Hz, 10-H); ^13^C-NMR δ_C_ (125 MHz, CDCl_3_) 24.4, 49.2, 53.6, 60.7, 80.9, 94.0, 106.3, 112.0, 112.1, 113.2, 118.5, 119.5, 128.8, 128.9, 129.5, 129.6, 132.9, 136.7, 142.7, 146.5, 152.2, 155.1; MS *m*/*z* 416 (100, MH^+^); HRMS (ES): MH^+^, found 416.1157. C_24_H_19_N_3_O_2_^35^Cl^+^ requires 416.1166.

### 3.7. Materials and Methods for in Vitro Cytotoxicity Assays

Human breast adenocarcinoma (MCF-7) cells and human cervical cancer (HeLa) cells used in this experiment were obtained from Cellonex (Johannesburg, South Africa). The cells were maintained in Dulbecco’s Modified Eagle’s (DMEM, HyClone, Thermo Scientific, Aalst, Belgium) supplemented with 0.4 mM l-glutamine and sodium pyruvate and 10% foetal bovine serum (FBS, HyClone, Thermo Scientific). The cells of a sub-confluent culture were harvested using trypsin-EDTA (HyClone, Thermo Scientific) and centrifuged at 200× *g* (where g is the relative centrifugal force) for 5 min. and re-suspended in growth medium to 5 × 10^4^ cells/mL. A total of 200 µL of the cell suspension was pipetted into each well of columns 2 to 11 of a 96 well culture plate. The same amount of the growth medium was added to wells of column 1 and 12 to maintain humidity and minimize the edge effect. The plates were incubated at 37 °C in a 5% CO_2_ incubator overnight until the cells were in the exponential phase of growth. After incubation, the DMEM was aspirated from the cells and replaced with 200 µL of different concentrations of the test samples (0.1–100 µg/mL). Each dilution of the test sample was tested in quadruplicate in each experiment and the experiments were repeated three times. The plates were again incubated for 2 days at 37 °C in a 5% incubator. A negative control (untreated cells) and positive control (cells treated with different concentrations of doxorubicin hydrochloride, Sigma, GmBH, Germany) were included. After incubation, 30 µL of 5 mg/mL MTT, (Sigma) in phosphate buffered saline PBS was added to each well and the plates were incubated for a further 4 h at 37 °C. The medium in each well was then removed and the formazan crystals formed were dissolved by adding 50 µL of DMSO to each well of the plates. The plates were gently shaken until the crystals were dissolved. The amount of MTT reduction was measured immediately by detecting the absorbance using a microplate reader at a wavelength of 570 nm (VersaMax, Molecular Devices, Sunnyvale, CA, USA). The wells in column 1 and 12, containing medium and MTT but no cells was used to blank the microplate reader. The percentage of cell viability was calculated using the formula below:

$$\%\text{Cell viability}=\frac{\text{Mean Absorbance of sample}}{\text{Mean Aborbance of control}}\times 100$$

The LC_50_ values (lethal concentration at which 50% of the cells are killed) were calculated as the concentration of the test sample that resulted in 50% reduction of absorbance compared to untreated cells. The intensity of the MTT formazan produced by living metabolically active cells is directly proportional to the number of live cells present [13].

## 4. Conclusions

In summary, we have demonstrated that the 5-aryl-9-bromo-7-iodo-2,3-dihydro-2*H*-imidazo[1,2-*c*]quinazoline scaffold undergoes palladium catalyzed sequential (Sonogashira/Suzuki-Miyaura) and one-pot successive (Sonogashira/Stille) cross-coupling reactions to afford novel unsymmetrical polycarbo-substituted derivatives. The one-pot two step Sonogashira/Stille cross-coupling reaction was accomplished with the use of a single catalyst complex by just varying the reaction time and temperature for the subsequent step. The imidazoquinazolines evaluated for anticancer activity were found to exhibit varying degrees of toxicity towards MCF-7 and HeLa cells. The SAR based on these preliminary *in vitro* cytotoxicity results reveal that the 4-fluorophenyl moiety at position 9 of the imidazoquinazoline framework is important for biological property. These preliminary *in vitro* cytotoxicity results and SAR, form a basis for the design and synthesis of more potent 9-(4-fluorophenyl)-substituted imidazoquinazolines.

## Supplementary Materials

The percentage cell viability (±standard deviation) and linear regression plots (used to calculate LC_50_ values) for doxorubicin hydrochloride and compounds **3**–**6** as well as the ^1^H- and ^13^C-NMR spectra of compounds **2**–**6** are listed in the supplementary materials. Supplementary materials can be accessed at: http://www.mdpi.com/1420-3049/20/12/19863/s1.
