# Supplementary material for: Novel Polycarbo-Substituted Imidazo[1,2-c]quinazolines: Synthesis and Cytotoxicity Study
Source: Molecules. 2015 Dec 15;20(12):22520–33. doi: 10.3390/molecules201219863 (PMC6331898; doi:10.3390/molecules201219863)
Supplement: Supplementary file 1 [file molecules-20-19863-s001.pdf]

Supplementary Materials: Novel Polycarbo-Substituted Imidazo[1,2-*c*]quinazolines: Synthesis and Cytotoxicity Study

Tebogo Ankie Khoza, Tshepiso Jan Makhafola and Malose Jack Mphahlele

S1: Percentage cell viability (± standard deviation) and linear regression plots (used to calculate LC_{50} values) of doxorubicin hydrochloride and compounds **3–6**.

S2: ^{1}H-NMR and ^{13}C-NMR spectra of compounds **3–6**.

Table S1. Percentage cell viability (± standard deviation) of MCF-7 and HeLa cells exposed to different concentrations of doxorubicin hydrochloride.

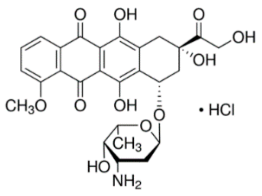

Doxorubicin hydrochloride.

Conc. (µg/mL)	Log conc.	%Viability MCF-7	SD	%Viability HeLa	SD
20	1.30103	22.59	0.87	4.78	0.75
10	1.0	25.98	0.68	4.96	0.91
5	0.69897	29.35	0.41	6.04	0.20
2	0.30103	33.58	0.39	19.92	0.92
1	0.0	34.97	0.94	47.38	0.62
0.5	−0.30103	54.97	0.45	50.73	0.59
0.1	−1.0	65.96	0.90	67.60	0.27

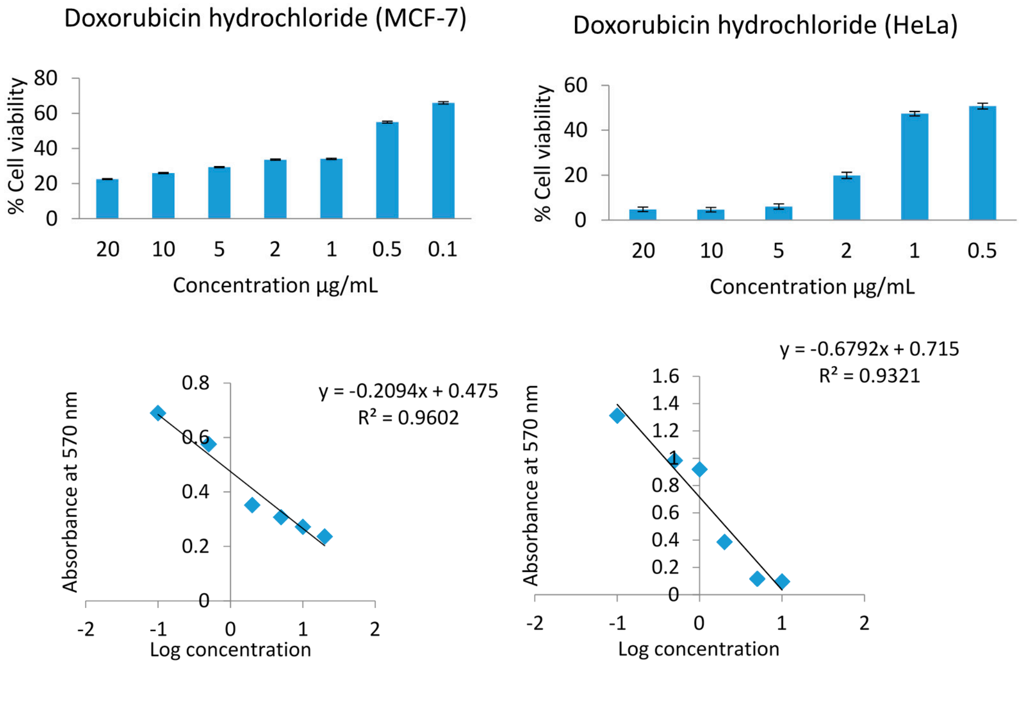

MCF-7 **HeLa**

Figure S1. Linear regression plots used to calculate LC_{50} values of doxorubicin hydrochloride.

Table S2. Percentage cell viability (± standard deviation) of MCF-7 and HeLa cells exposed to different concentrations of compound **3a.**

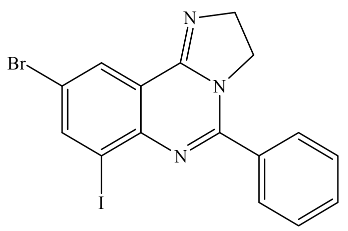

3a

Conc. (µg/mL)	Log conc.	%Viability MCF-7	SD	%Viability HeLa	SD
**100**	**2.0**	**20.07**	**0.21**	**18.77** **59.87**	**1.10**
10	1.0	40.06	0.40	59.87	0.72
1	0.0	57.41	0.90	92.32	0.95
0.1	-1.0	88.15	1.36	97.92	1.05

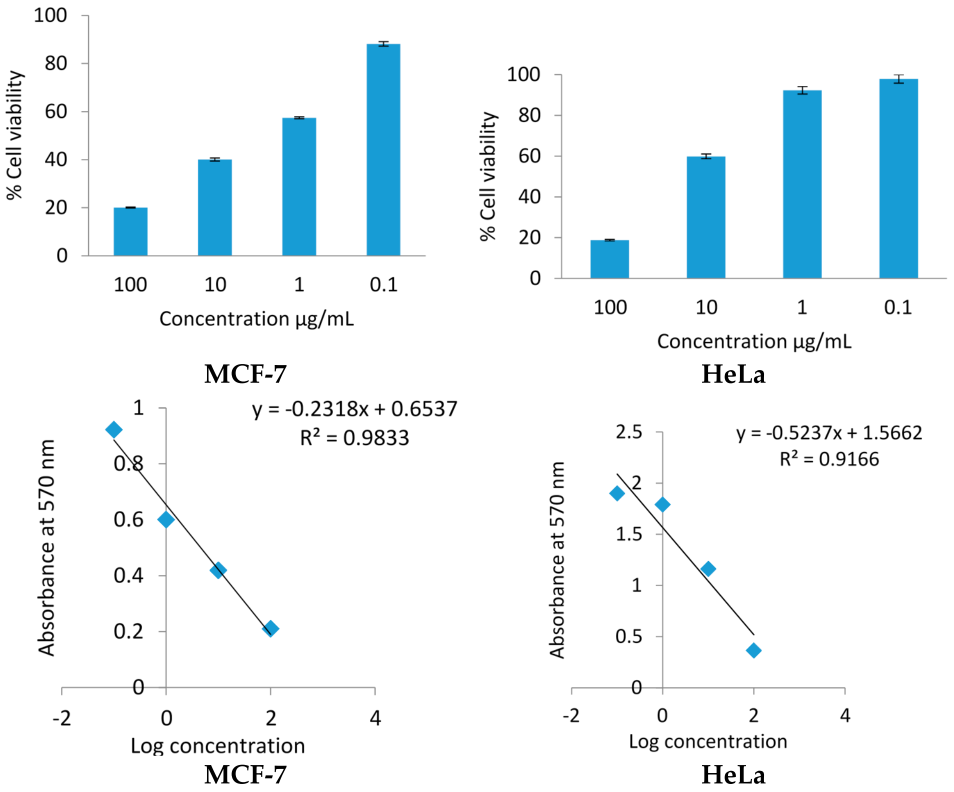

Figure S2. Linear regression plots used to calculate LC_{50} values of compound **3a**.

Table S3. Percentage cell viability (± standard deviation) of MCF-7 and HeLa cells exposed to different concentrations of compound **3b.**

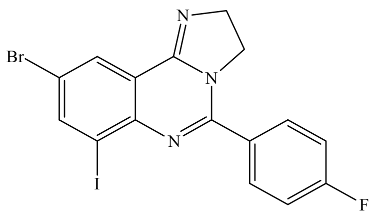

3b

Conc. (μg/mL)	Log conc.	%Viability MCF-7	SD	%Viability HeLa	SD
**100**	**2.0**	**23.99**	**0.21**	**37.21**	**0.62**
10	1.0	29.68	0.60	46.14	0.98
1	0.0	47.08	0.41	63.67	1.14
0.1	-1.0	57.05	0.92	83.62	0.24

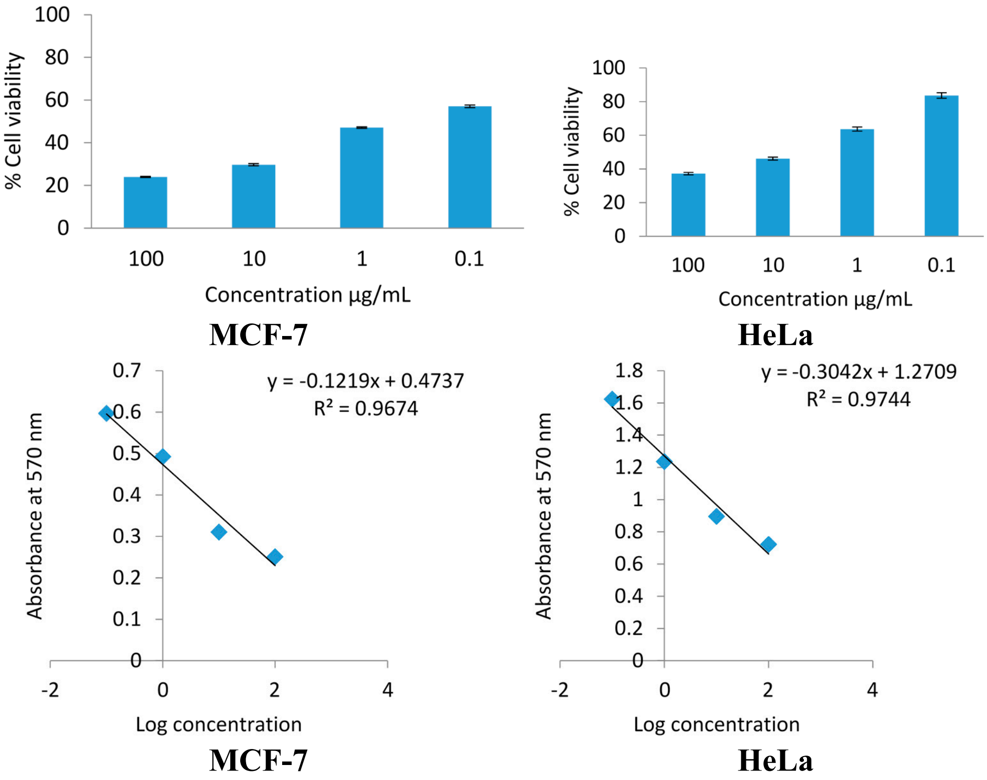

Figure S3. Linear regression plots used to calculate LC_{50} values of compound **3b**.

Table S4. Percentage cell viability (± standard deviation) of MCF-7 and HeLa cells exposed to different concentrations of compound **3c.**

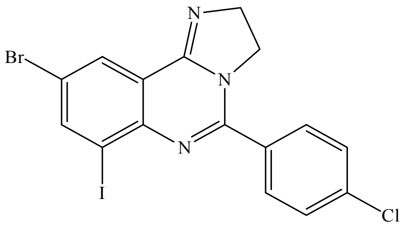

3c

Conc. (μg/mL)	Log conc.	%Viability MCF-7	SD	%Viability HeLa	SD
**100**	**2.0**	**26.15**	**0.42**	**29.35**	**0.18**
10	1.0	32.15	0.54	46.25	0.99
1	0.0	44.50	0.40	56.83	0.95
0.1	-1.0	65.442	0.80	95.64	1.20

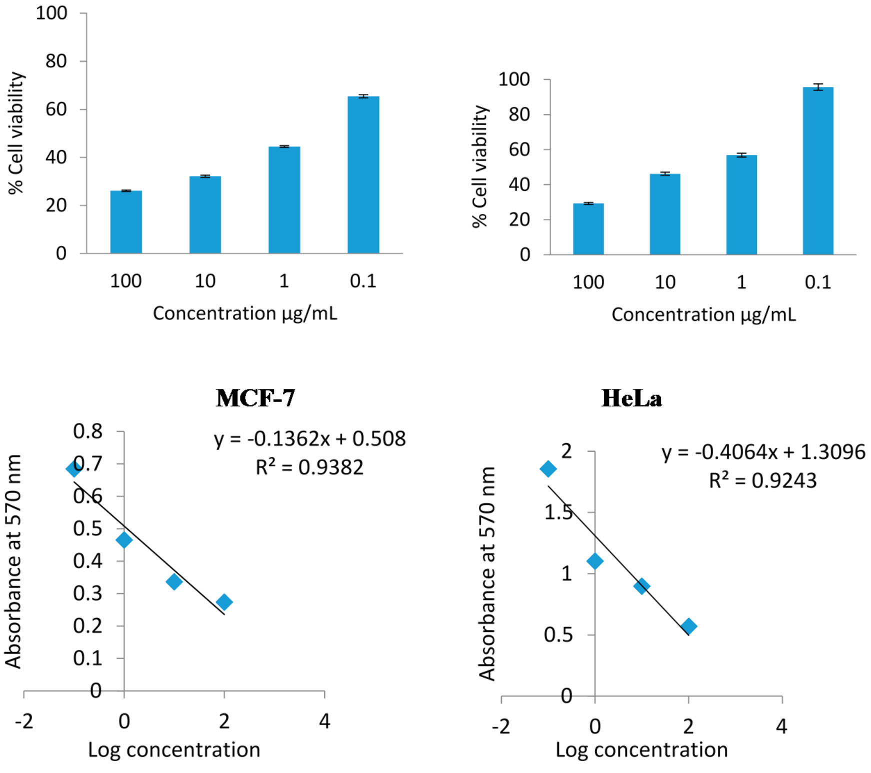

MCF-7 **HeLa**

Figure S4. Linear regression plots used to calculate LC50 values of compound **3c**.

Table S5. Percentage cell viability (± standard deviation) of MCF-7 and HeLa cells exposed to different concentrations of compound **4a**.

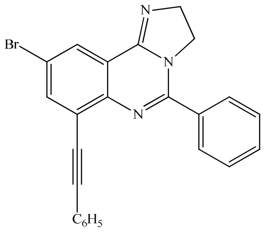

4a

Conc. (µg/mL)	Log conc.	%Viability MCF-7	SD	%Viability HeLa	SD
100	2.0	39.37	0.23	29.32	0.48
10	1.0	41.20	0.43	51.54	0.13
1	0.0	50.57	0.62	84.57	0.49
0.1	-1.0	59.37	0.35	98.12	0.90

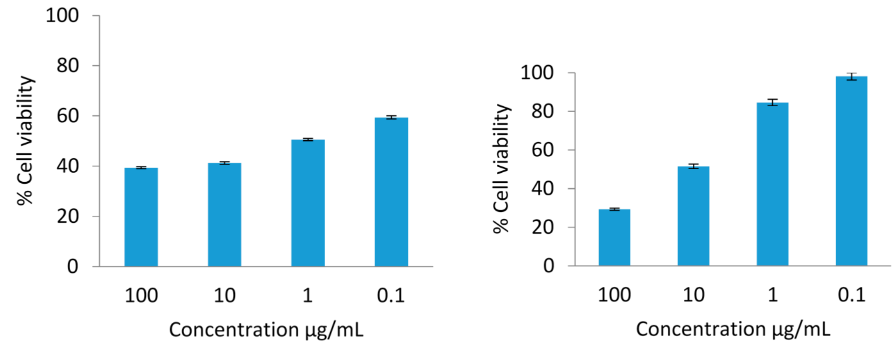

MCF-7 **HeLa**

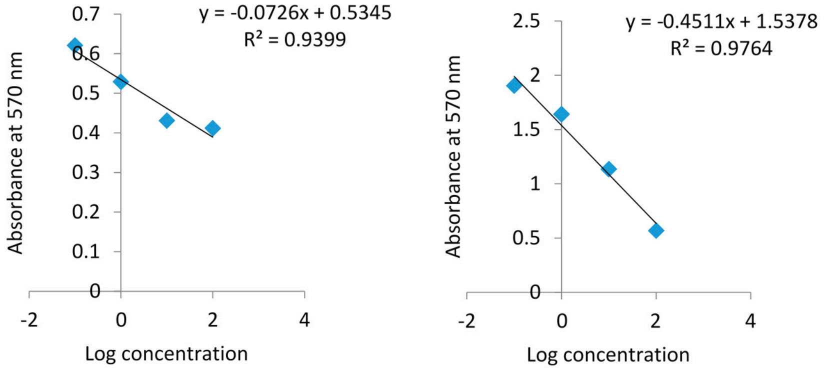

MCF-7 **HeLa**

Figure S5. Linear regression plots used to calculate LC_{50} values of compound **4a**.

Table S6. Percentage cell viability (± standard deviation) of MCF-7 and HeLa cells exposed to different concentrations of compound **4b**.

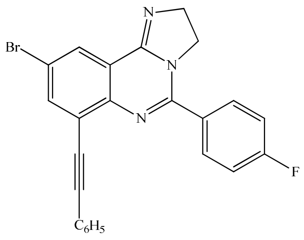

4b

Conc. (µg/mL)	Log conc.	%Viability MCF-7	SD	%Viability HeLa	SD
**100**	**2.0**	**40.95**	**0.18**	**32.26**	**0.20**
10	1.0	52.10	0.75	36.99	0.61
1	0.0	73.56	0.32	73.61	0.98
0.1	−1.0	100	0.75	91.52	1.06

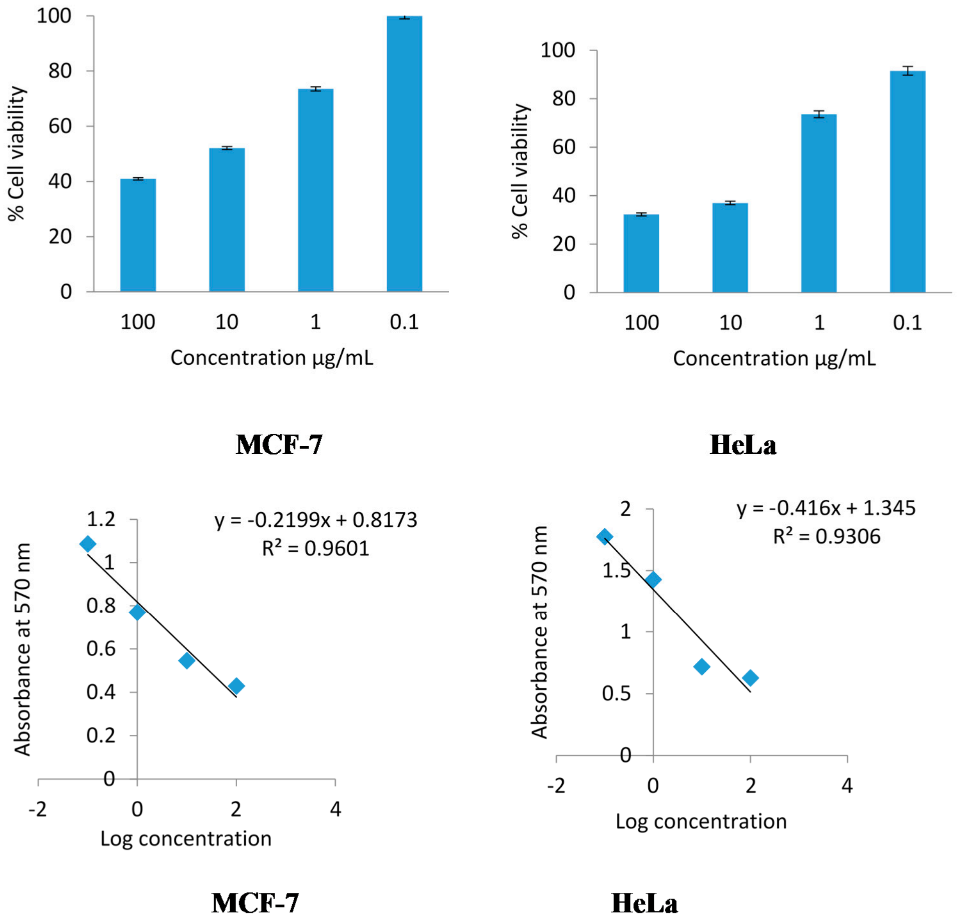

Figure S6. Linear regression plots used to calculate LC_{50} values of compound **4b**.

Table S7. Percentage cell viability (± standard deviation) of MCF-7 and HeLa cells exposed to different concentrations of compound **4c**.

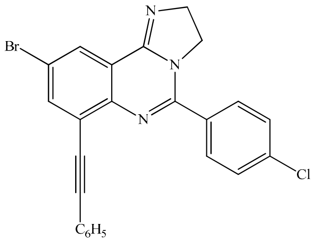

4c

Conc. (µg/mL)	Log conc.	%Viability MCF-7	SD	%Viability HeLa	SD
**100**	**2.0**	**36.99**	**0.17**	**25.77**	**0.96**
10	1.0	56.48	0.06	42.36	1.65
1	0.0	58.87	0.25	78.51	1.81
0.1	−1.0	85.32	0.20	89.51	0.86

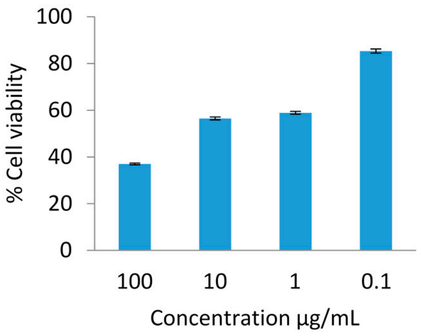

MCF-7

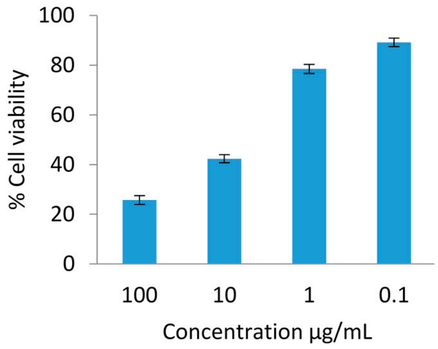

HeLa

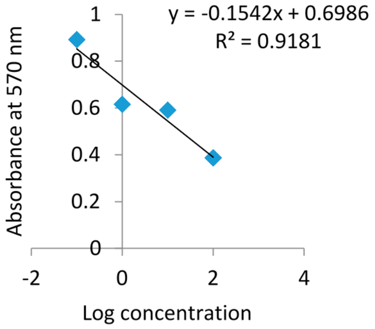

MCF-7

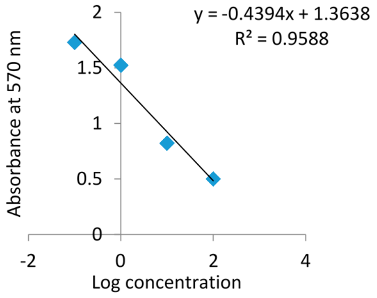

HeLa

Figure S7. Linear regression plots used to calculate LC_{50} values of compound **4c**.

Table S8. Percentage cell viability (± standard deviation) of MCF-7 and HeLa cells exposed to different concentrations of compound **4d**.

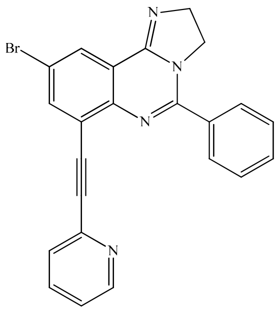

4d

Conc. (µg/mL)	Log conc.	%Viability MCF-7	SD	%Viability HeLa	SD
**100**	**2.0**	**19.39**	**0.38**	**17.87**	**0.65**
10	1.0	39.29	0.52	33.58	1.4
1	0.0	48.83	0.65	47.23	0.1
0.1	−1.0	75.91	1.4	50.52	0.43

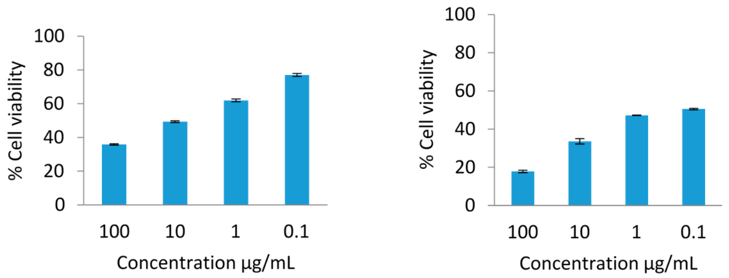

MCF-7 **HeLa**

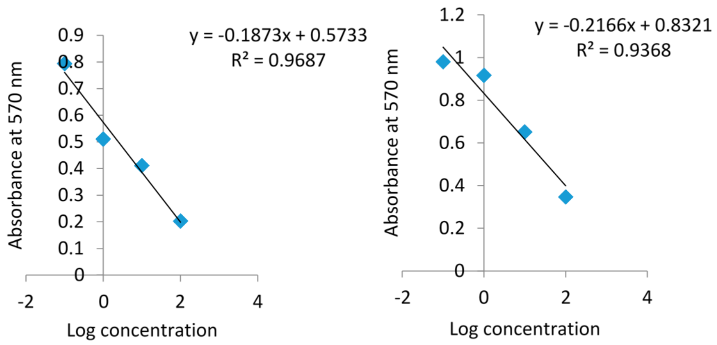

MCF-7 **HeLa**

Figure S8. Linear regression plots used to calculate LC_{50} values of compound **4d**.

Table S9. Percentage cell viability (± standard deviation) of MCF-7 and HeLa cells exposed to different concentrations of compound **4f**.

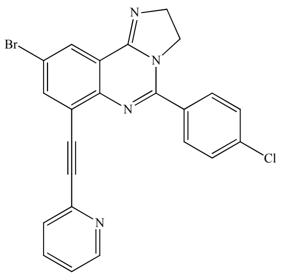

4f

Conc. (µg/mL)	Log conc.	%Viability MCF-7	SD	%Viability HeLa	SD
**100**	**2.0**	**35.83**	**0.05**	**21.71**	**0.03**
10	1.0	49.36	0.70	25.74	0.14
1	0.0	61.95	0.13	51.58	0.10
0.1	−1.0	77.01	0.10	8831	0.43

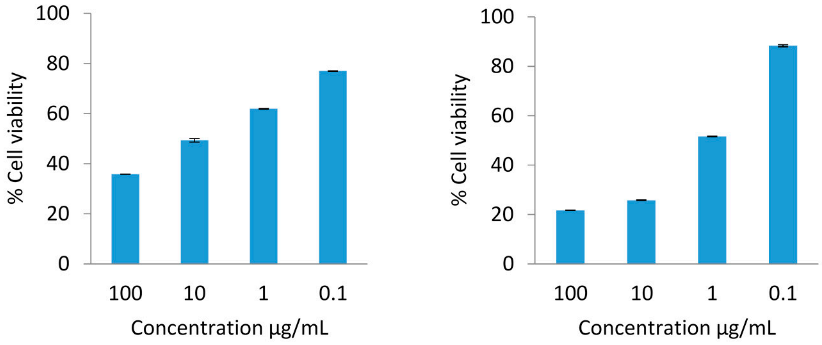

MCF-7 **HeLa**

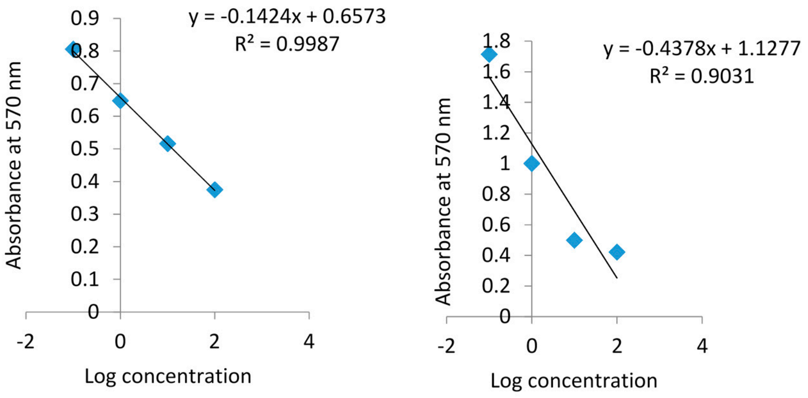

MCF-7 **HeLa**

Figure S9. Linear regression plots used to calculate LC_{50} values of compound **4f**.

Table S10. Percentage cell viability (± standard deviation) of MCF-7 and HeLa cells exposed to different concentrations of compound **4g**.

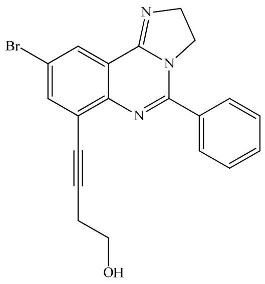

4g

Conc. (µg/mL)	Log conc.	%Viability MCF-7	SD	%Viability HeLa	SD
**100**	**2.0**	**21.49**	**0.23**	**29.60**	**0.77**
10	1.0	53.00	0.09	53.74	0.29
1	0.0	60.69	0.27	85.67	0.30
0.1	−1.0	65.20	0.15	87.25	0.40

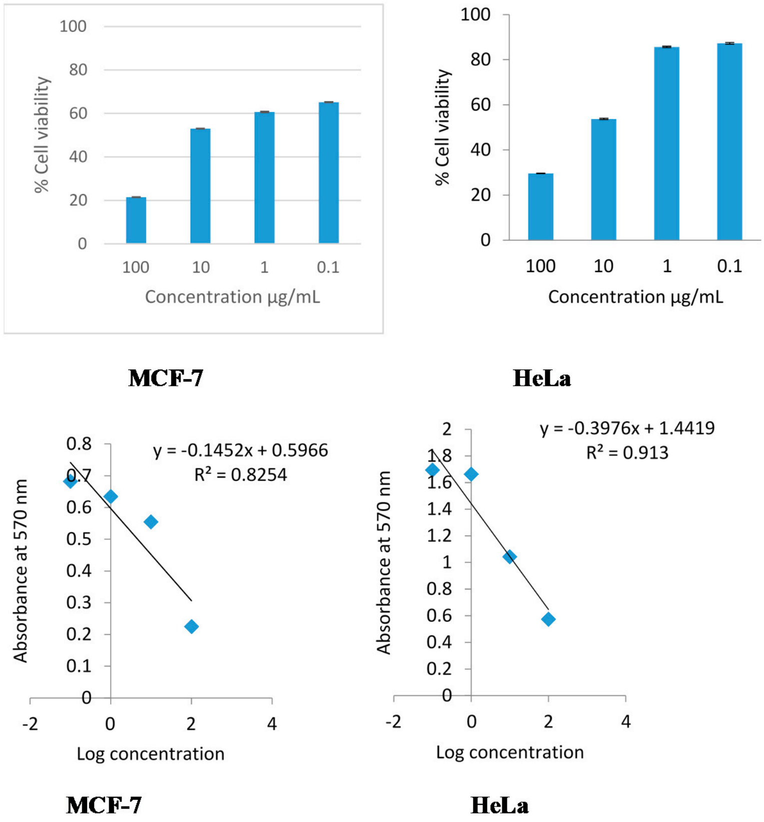

Figure S10. Linear regression plots used to calculate LC_{50} values of compound **4g**.

Table S11. Percentage cell viability (± standard deviation) of MCF-7 and HeLa cells exposed to different concentrations of compound **4i**.

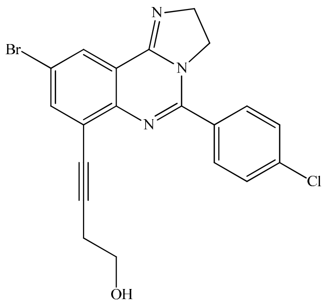

4i

Conc. (µg/mL)	Log conc.	%Viability MCF-7	SD	%Viability HeLa	SD
**100**	**2.0**	**33.84**	**0.71**	**33.47**	**0.11**
10	1.0	49.90	0.11	52.13	0.36
1	0.0	62.70	0.30	92.79	0.34
0.1	−1.0	73.50	0.15	100.00	0.24

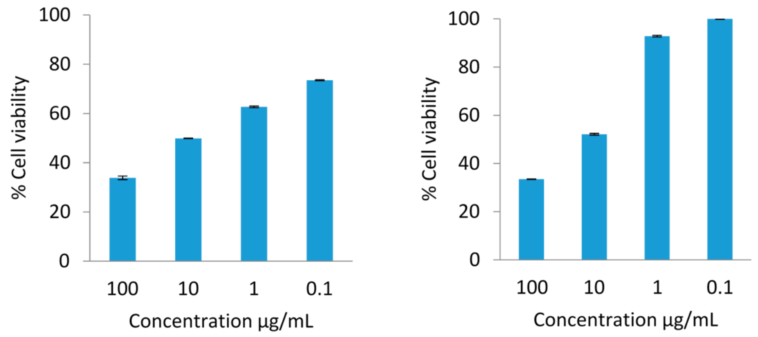

MCF-7 **HeLa**

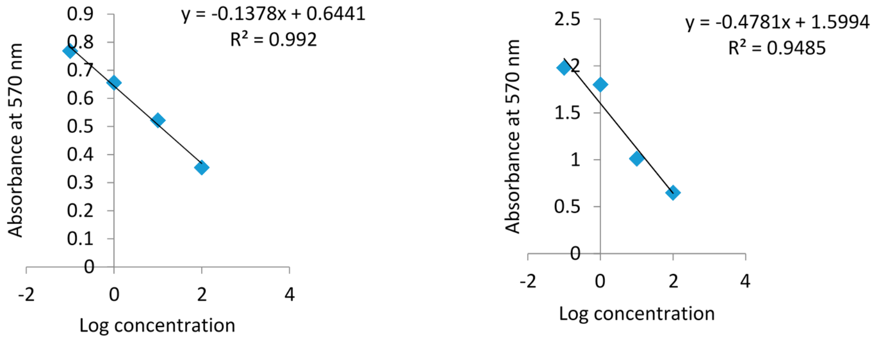

MCF-7 **HeLa**

Figure S11. Linear regression plots used to calculate LC_{50} values of compound **4i**.

Table S12. Percentage cell viability (± standard deviation) of MCF-7 and HeLa cells exposed to different concentrations of compound **5a.**

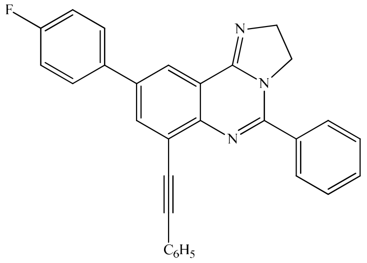

5a

Conc. (µg/mL)	Log conc.	%Viability MCF-7	SD	%Viability HeLa	SD
**100**	**2.0**	**38.36**	**0.04**	**24.55**	**0.01**
10	1.0	48.59	0.08	27.66	0.11
1	0.0	53.66	0.05	52.20	0.09
0.1	−1.0	64.15	0.20	77.01	0.42

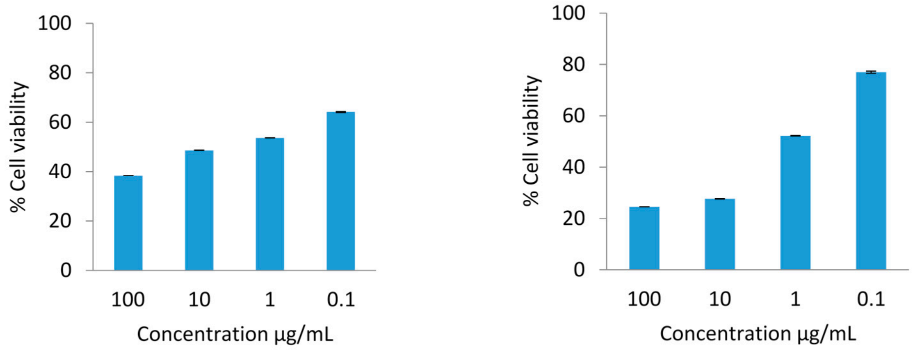

MCF-7 **HeLa**

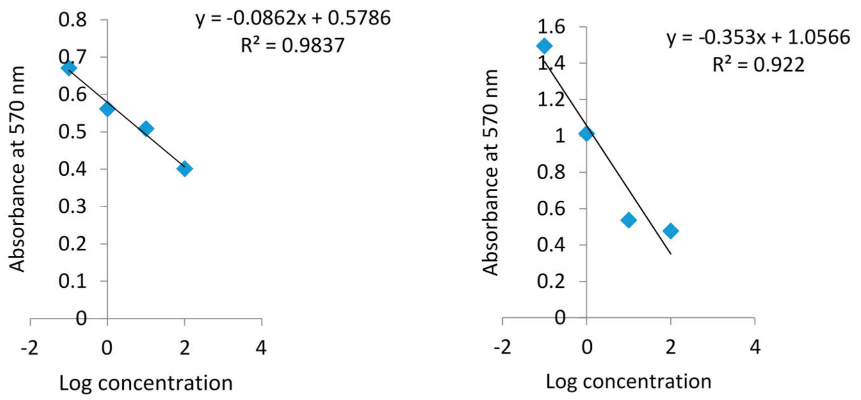

MCF-7 **HeLa**

Figure S12. Linear regression plots used to calculate LC_{50} values of compound **5a**.

Table S13. Percentage cell viability (± standard deviation) of MCF-7 and HeLa cells exposed to different concentrations of compound **5b**.

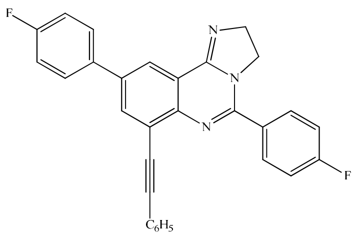

5b

Conc. (µg/mL)	Log conc.	%Viability MCF-7	SD	%Viability HeLa	SD
**100**	**2.0**	**9.68**	**0.30**	**21.43**	**0.04**
10	1.0	17.54	0.41	22.26	0.13
1	0.0	20.12	0.56	45.47	0.19
0.1	−1.0	37.76	0.76	64.06	0.28

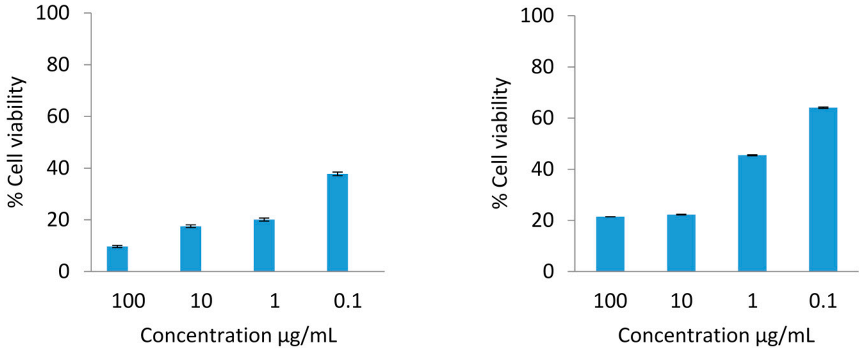

MCF-7 **HeLa**

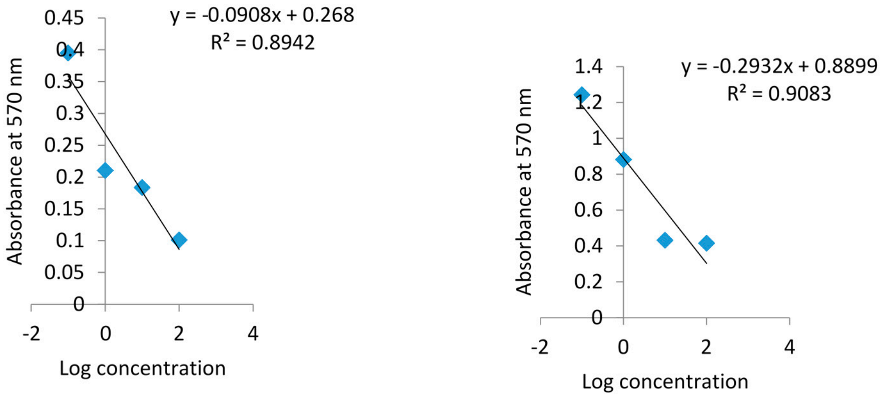

MCF-7 **HeLa**

Figure S13. Linear regression plots used to calculate LC_{50} values of compound **5b**.

Table S14. Percentage cell viability (± standard deviation) of MCF-7 and HeLa cells exposed to different concentrations of compound **5c**.

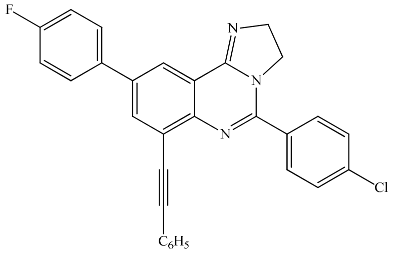

5c

Conc. (µg/mL)	Log conc.	%Viability MCF-7	SD	%Viability HeLa	SD
**100**	**2.0**	**35.52**	**0.08**	**38.23**	**0.09**
10	1.0	41.87	0.07	38.85	0.13
1	0.0	42.69	0.06	47.98	0.16
0.1	-1.0	43.07	0.09	56.31	0.30

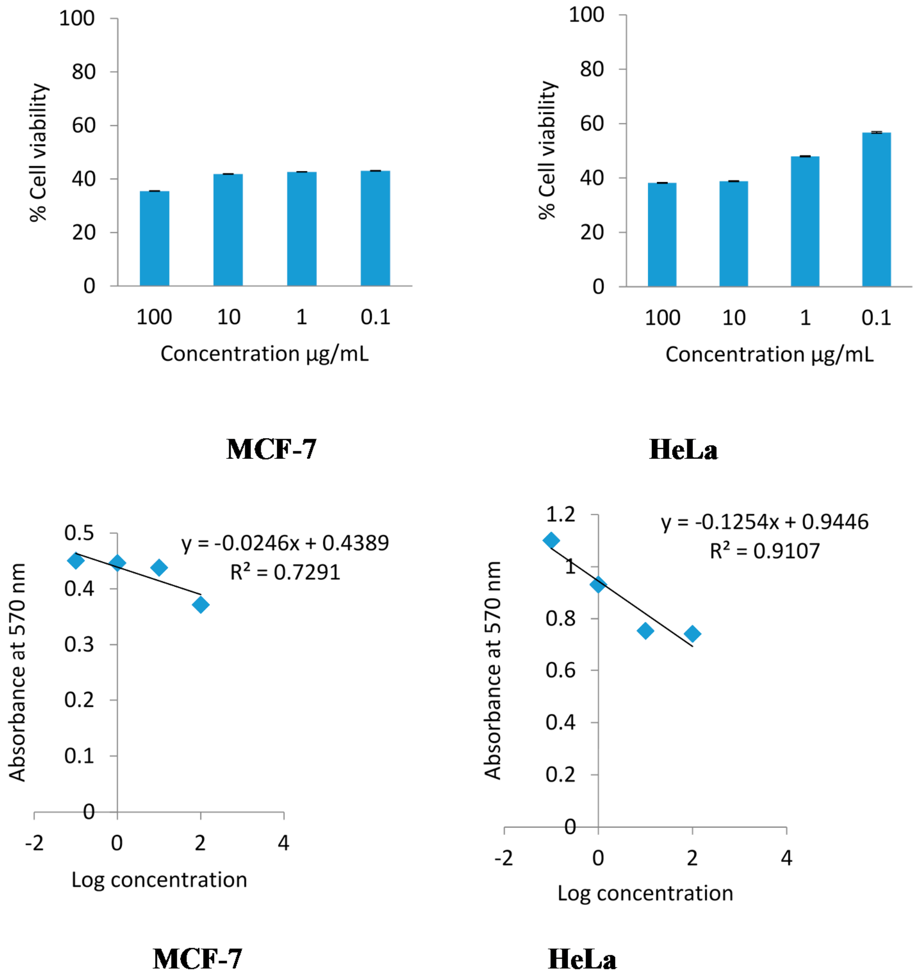

Figure S14. Linear regression plots used to calculate LC_{50} values of compound **5c**.

Table S15. Percentage cell viability (± standard deviation) of MCF-7 and HeLa cells exposed to different concentrations of compound **5e**.

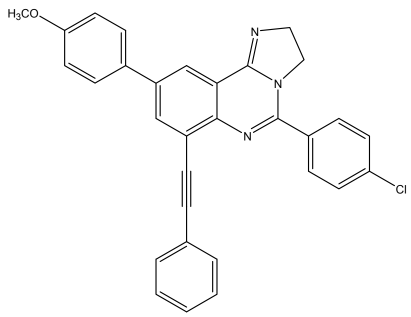

5e

Conc. (µg/mL)	Log conc.	%Viability MCF-7	SD	%Viability HeLa	SD
**100**	**2.0**	**30.55**	**0.14**	**22.19**	**0.05**
10	1.0	51.15	0.09	37.03	0.08
1	0.0	55.00	0.26	47.19	0.13
0.1	−1.0	79.92	0.15	60.65	0.07

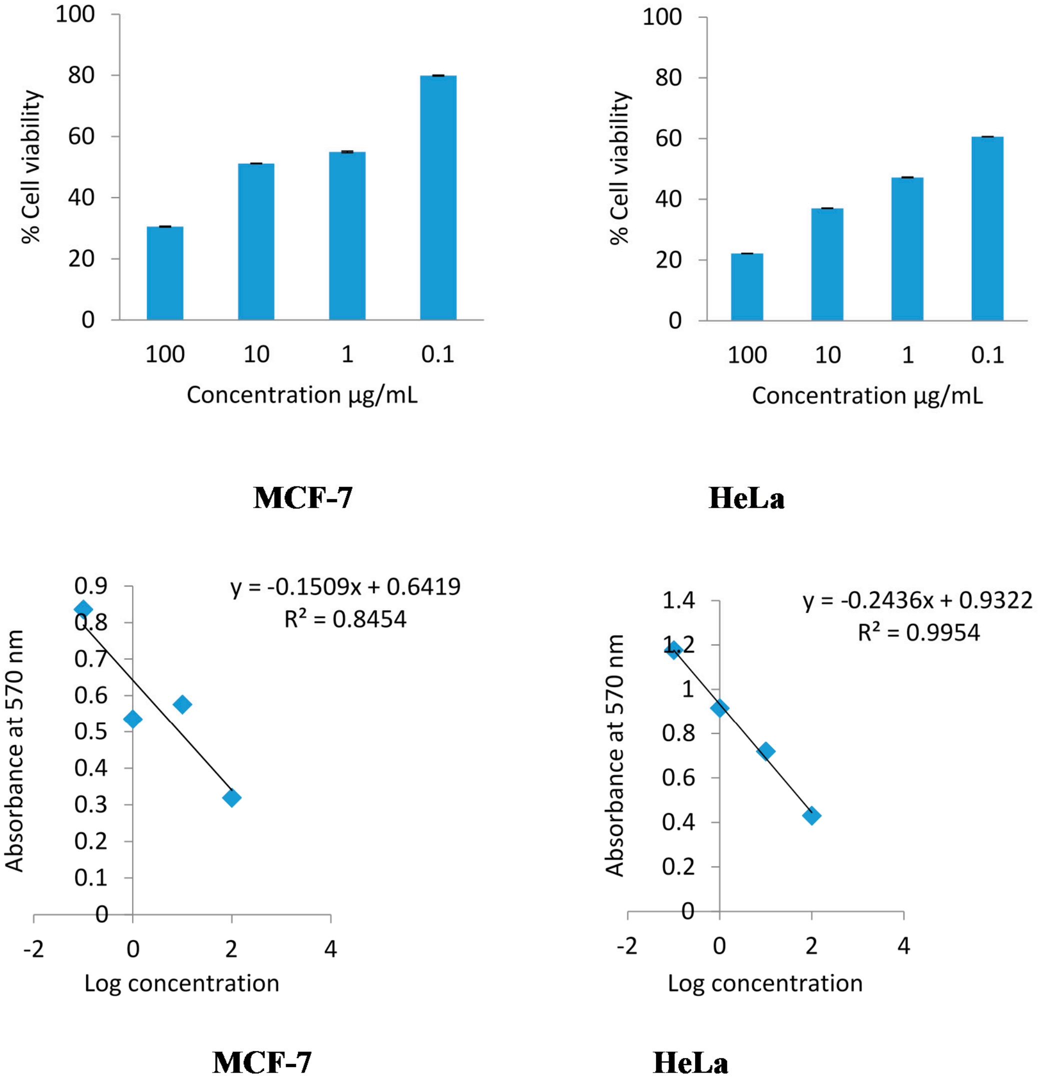

Figure S15. Linear regression plots used to calculate LC_{50} values of compound **5e**

Table S16. Percentage cell viability (± standard deviation) of MCF-7 and HeLa cells exposed to different concentrations of compound **5f.**

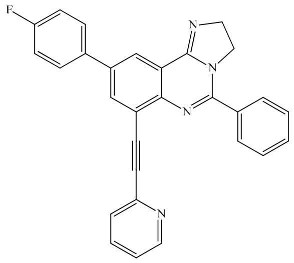

5f

Conc. (µg/mL)	Log conc.	%Viability MCF-7	SD	%Viability HeLa	SD
**100**	**2.0**	**24.76**	**0.02**	**17.07**	**0.03**
10	1.0	51.85	0.04	35.69	0.13
1	0.0	69.31	0.08	44.70	0.14
0.1	−1.0	83.94	0.13	85.41	0.31

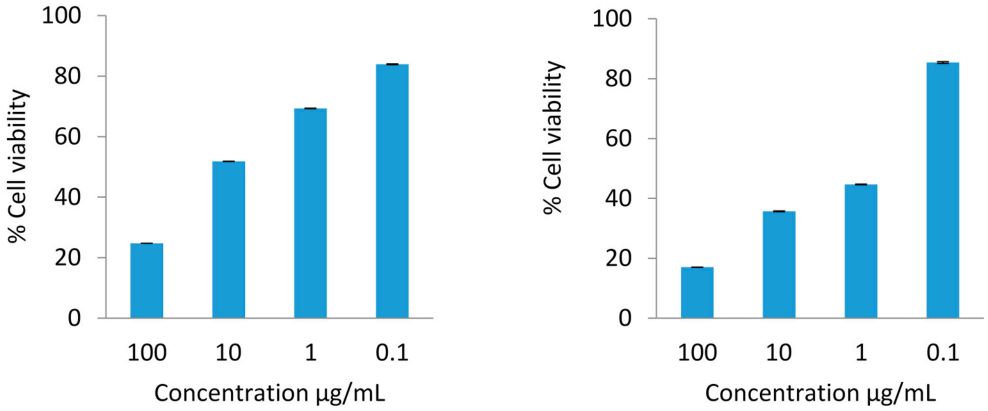

MCF-7 **HeLa**

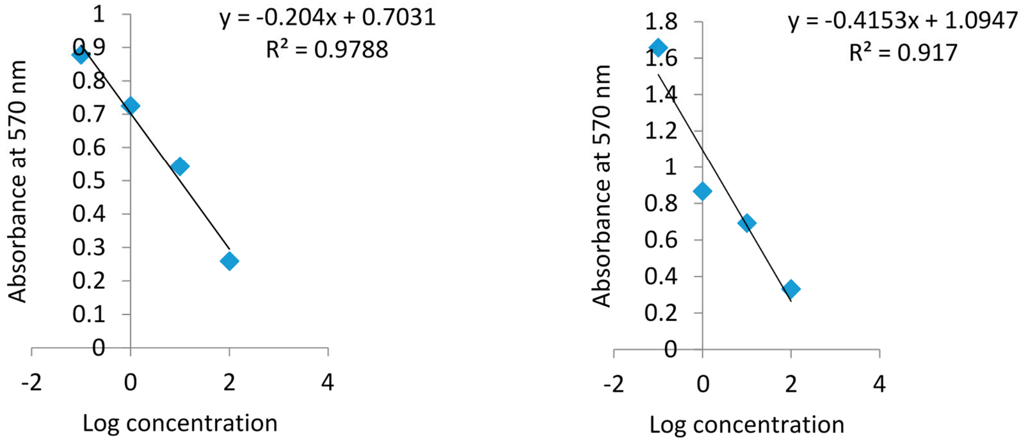

MCF-7 **HeLa**

Figure S16. Linear regression plots used to calculate LC_{50} values of compound **5f**

Table S17. Percentage cell viability (± standard deviation) of MCF-7 and HeLa cells exposed to different concentrations of compound **5g.**

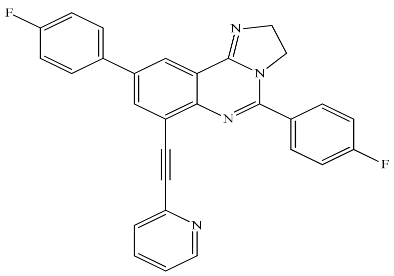

5g

Conc. (µg/mL)	Log conc.	%Viability MCF-7	SD	%Viability HeLa	SD
**100**	**2.0**	**36.71**	**0.02**	**25.74**	**0.05**
10	1.0	52.28	0.05	38.88	0.08
1	0.0	63.89	0.03	63.53	0.21
0.1	−1.0	82.07	0.17	77.47	0.16

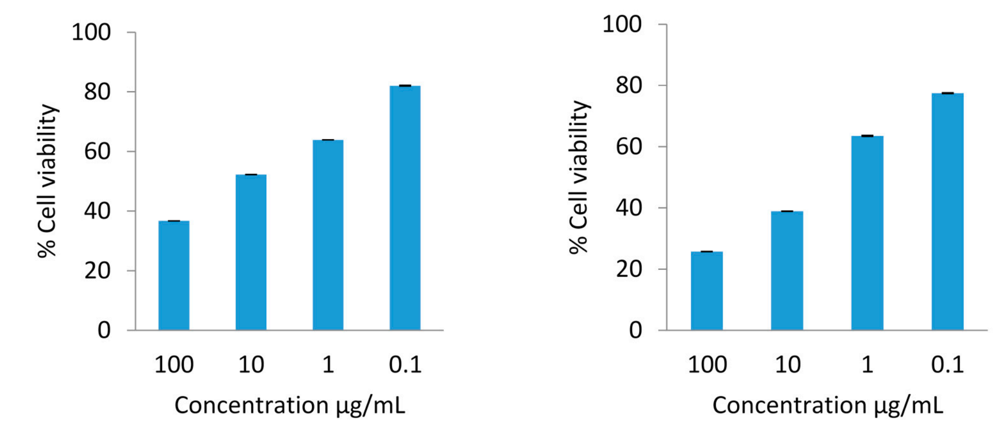

MCF-7 **HeLa**

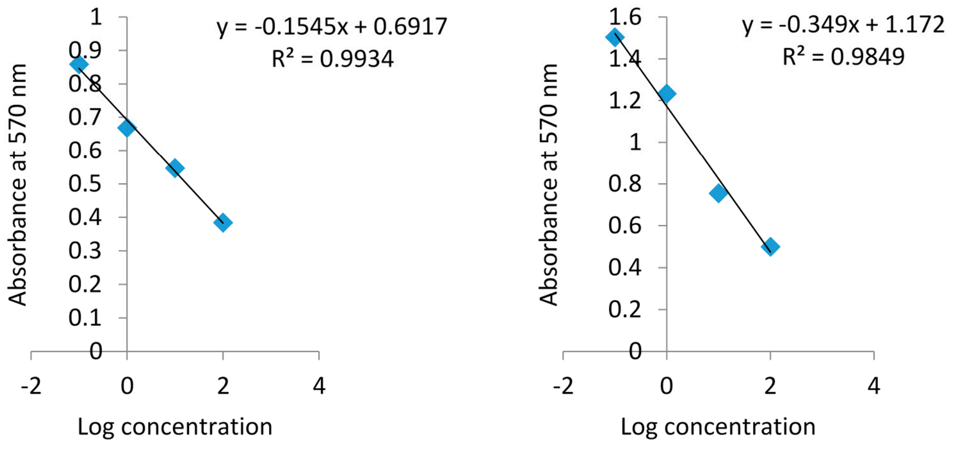

MCF-7 **HeLa**

Figure S17. Linear regression plots used to calculate LC_{50} values of compound **5g**

Table S18. Percentage cell viability (± standard deviation) of MCF-7 and HeLa cells exposed to different concentrations of compound **5h.**

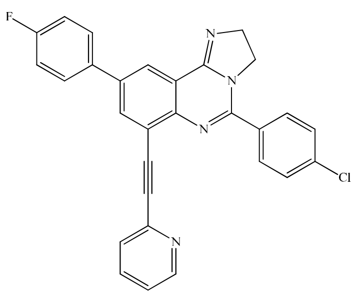

5h

Conc. (µg/mL)	Log conc.	%Viability MCF-7	SD	%Viability HeLa	SD
**100**	**2.0**	**21.75**	**0.13**	**17.78**	**0.01**
**10**	**1.0**	**32.03**	0.04	31.90	0.04
1	0.0	40.01	0.2	33.37	0.2
0.1	−1.0	59.54	0.10	58.45	0.09

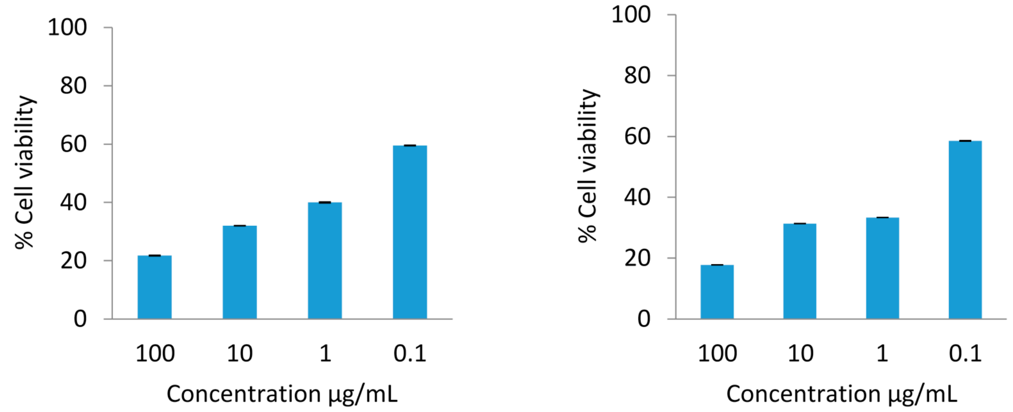

MCF-7 **HeLa**

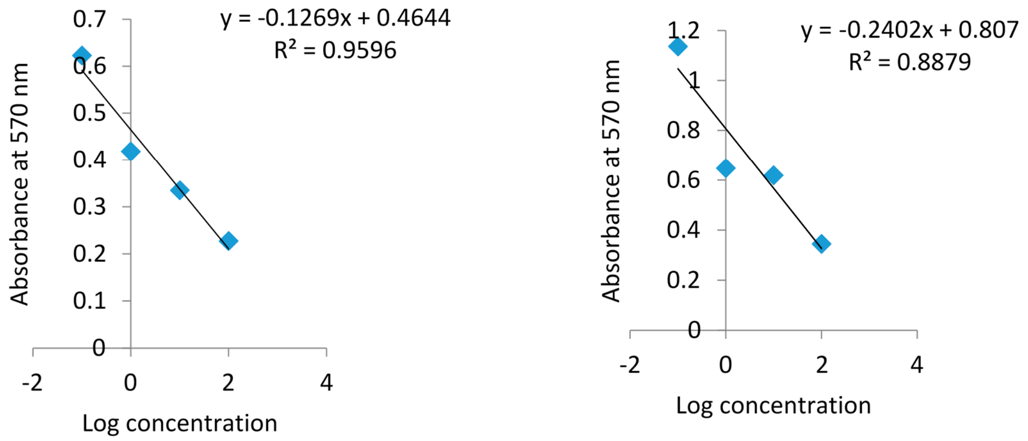

MCF-7 **HeLa**

Figure S18. Linear regression plots used to calculate LC_{50} values of compound **5h.**

Table S19. Percentage cell viability (± standard deviation) of MCF-7 and HeLa cells exposed to different concentrations of compound **5i.**

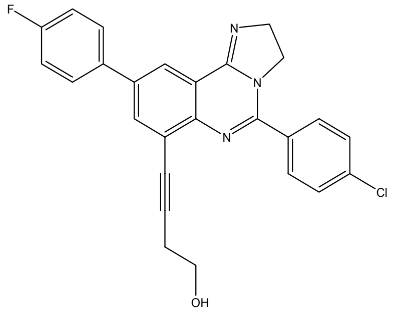

5i

Conc. (µg/mL)	Log conc.	%Viability MCF-7	SD	%Viability HeLa	SD
**100**	**2.0**	**47.59**	**0.10**	**32.33**	**0.08**
10	1.0	54.45	0.09	62.68	0.26
1	0.0	58.06	0.11	82.43	0.19
0.1	−1.0	59.68	0.04	91.70	0.39

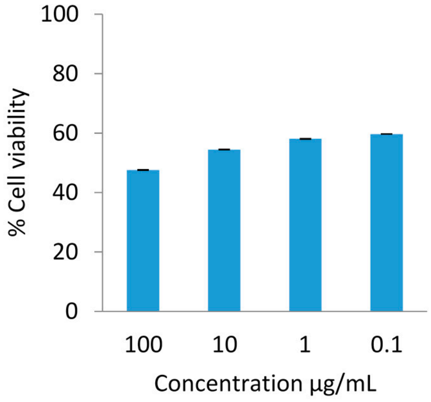

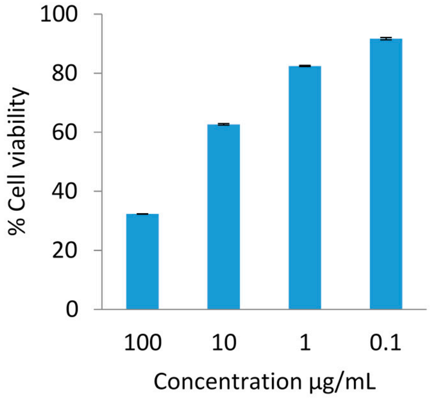

MCF-7 **HeLa**

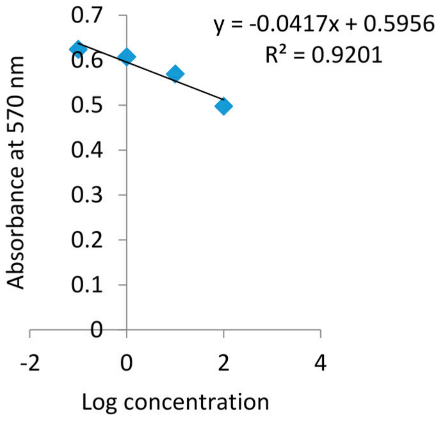

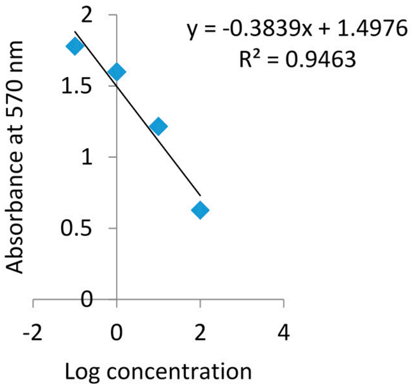

MCF-7 **HeLa**

Figure S19. Linear regression plots used to calculate LC_{50} values of compound **5i.**

Table S20. Percentage cell viability (± standard deviation) of MCF-7 and HeLa cells exposed to different concentrations of compound **6a.**

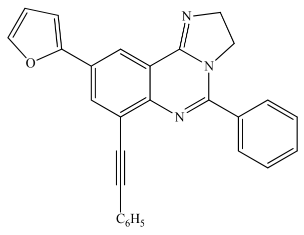

6a

Conc. (µg/mL)	Log conc.	%Viability MCF-7	SD	%Viability HeLa	SD
**100**	**2.0**	**37.48**	**0.03**	**67.67**	**0.29**
10	1.0	39.85	0.18	74.62	0.38
1	0.0	55.40	0.09	93.20	0.70
0.1	−1.0	73.55	0.12	97.92	0.28

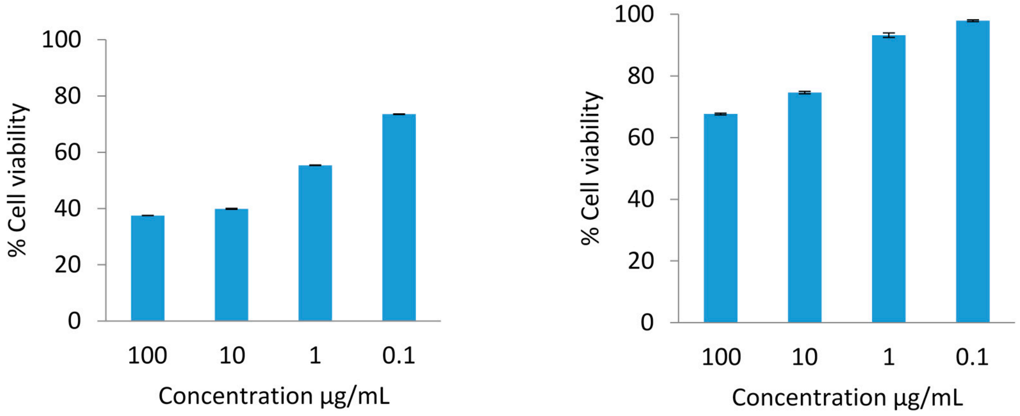

MCF-7 **HeLa**

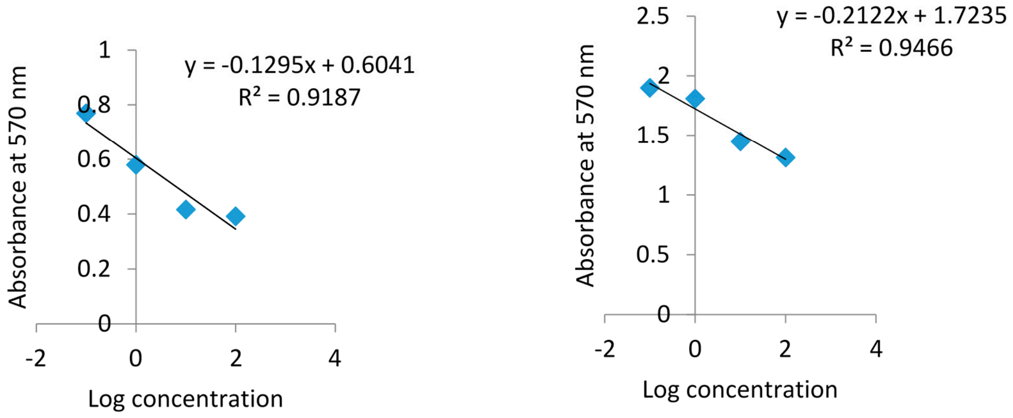

MCF-7 **HeLa**

Figure S20. Linear regression plots used to calculate LC_{50} values of compound **6a**.

Table S21. Percentage cell viability (± standard deviation) of MCF-7 and HeLa cells exposed to different concentrations of compound **6b.**

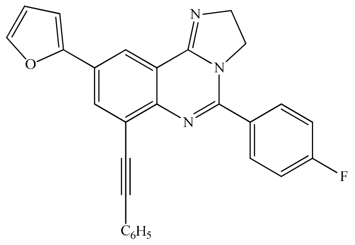

6b

Conc. (µg/mL)	Log conc.	%Viability MCF-7	SD	%Viability HeLa	SD
100	2.0	45.55	0.08	24.79	0.02
10	1.0	86.04	0.10	34.53	0.19
1	0.0	87.81	0.05	39.53	0.42
0.1	−1.0	93.31	0.13	97.92	0.24

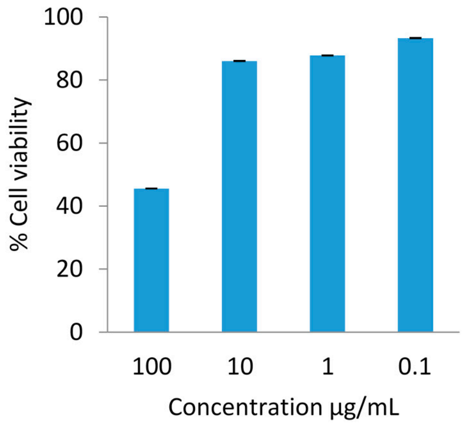

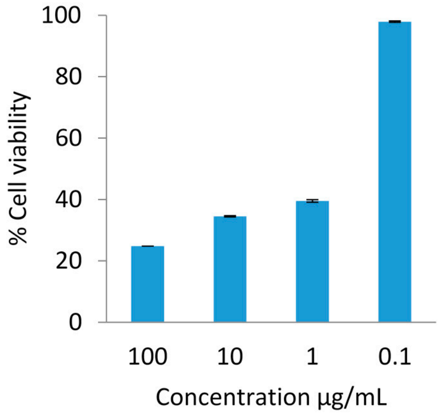

MCF-7 **HeLa**

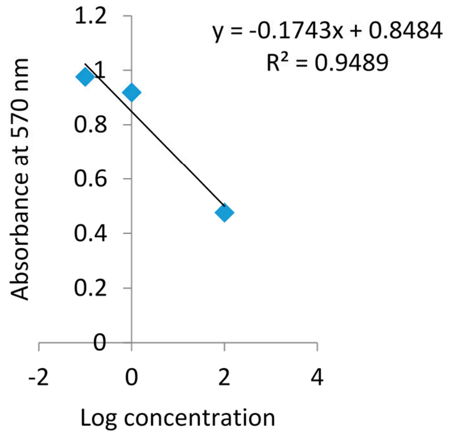

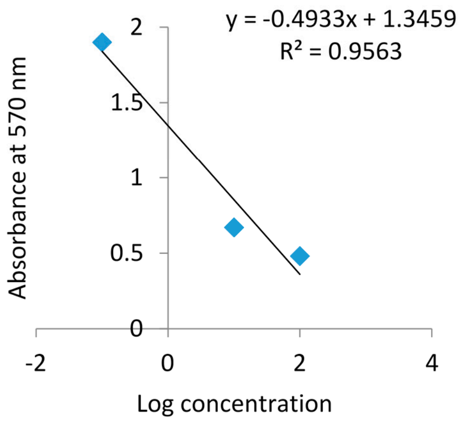

MCF-7 **HeLa**

Figure S21. Linear regression plots used to calculate LC_{50} values of compound **6b**

Table S22. Percentage cell viability (± standard deviation) of MCF-7 and HeLa cells exposed to different concentrations of compound **6c.**

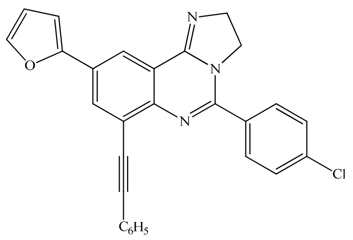

6c

Conc. (µg/mL)	Log conc.	%Viability MCF-7	SD	%Viability HeLa	SD
**100**	**2.0**	**55.43**	**0.06**	**27.06**	**0.07**
10	1.0	60.13	0.08	29.36	0.13
1	0.0	64.45	0.07	3.27	0.17
0.1	−1.0	69.07	0.14	58.80	0.46

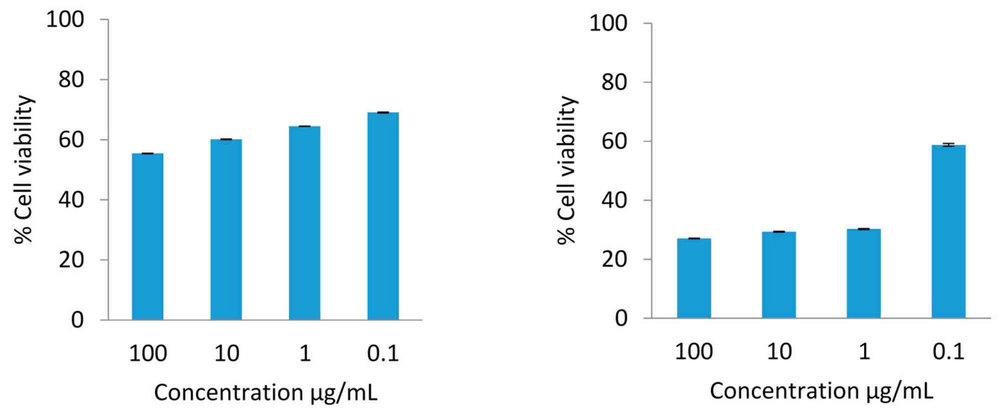

MCF-7 **HeLa**

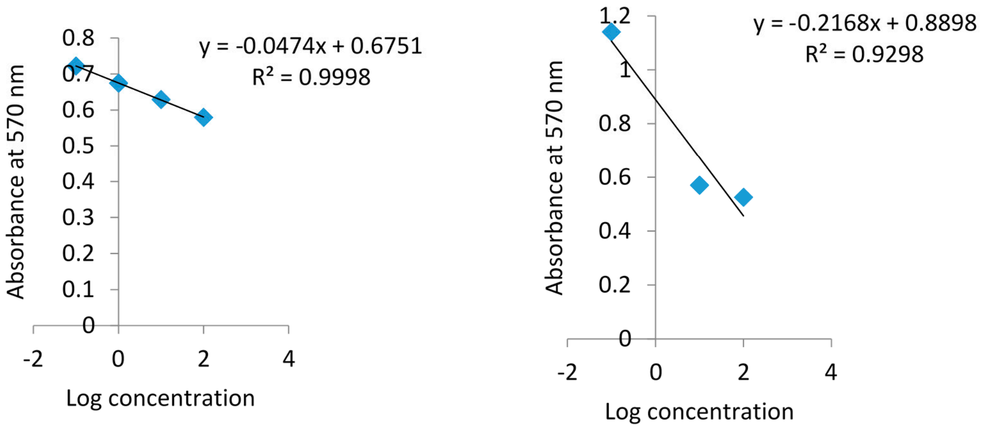

MCF-7 **HeLa**

Figure s22. Linear regression plots used to calculate LC_{50} values of compound **6c**

Table S23. Percentage cell viability (± standard deviation) of MCF-7 and HeLa cells exposed to different concentrations of compound **6e.**

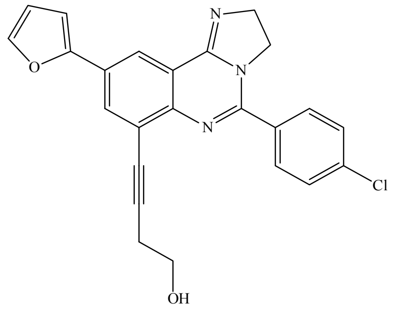

6e

Conc. (µg/mL)	Log conc.	%Viability MCF-7	SD	%Viability HeLa	SD
100	2.0	5.69	0.01	2.39	0.01
10	1.0	48.85	0.08	38.56	0.38
1	0.0	87.6	0.07	66.39	0.24
0.1	−1.0	90.30	0.14	69.75	0.36

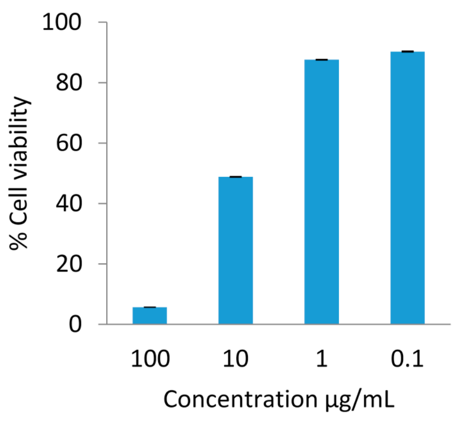

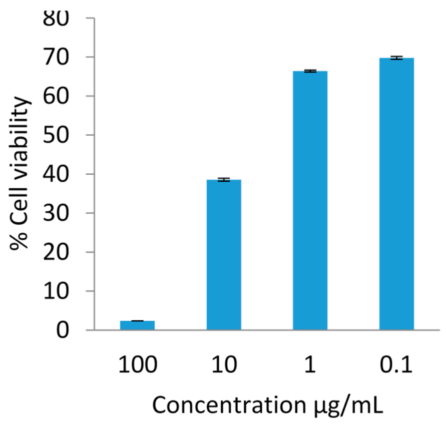

MCF-7 **HeLa**

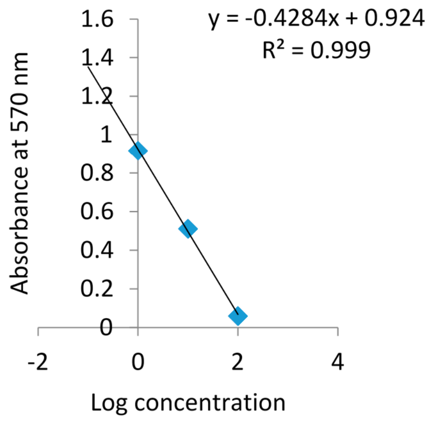

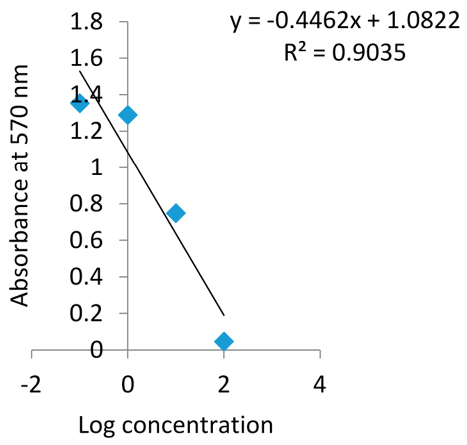

MCF-7 **HeLa**

Figure 23. Linear regression plots used to calculate LC_{50} values of compound **6e.**

S2: ¹H-NMR and ¹³C-NMR spectra of compounds 2–6.

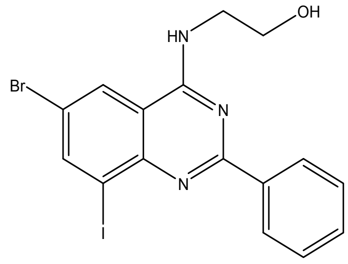

2a

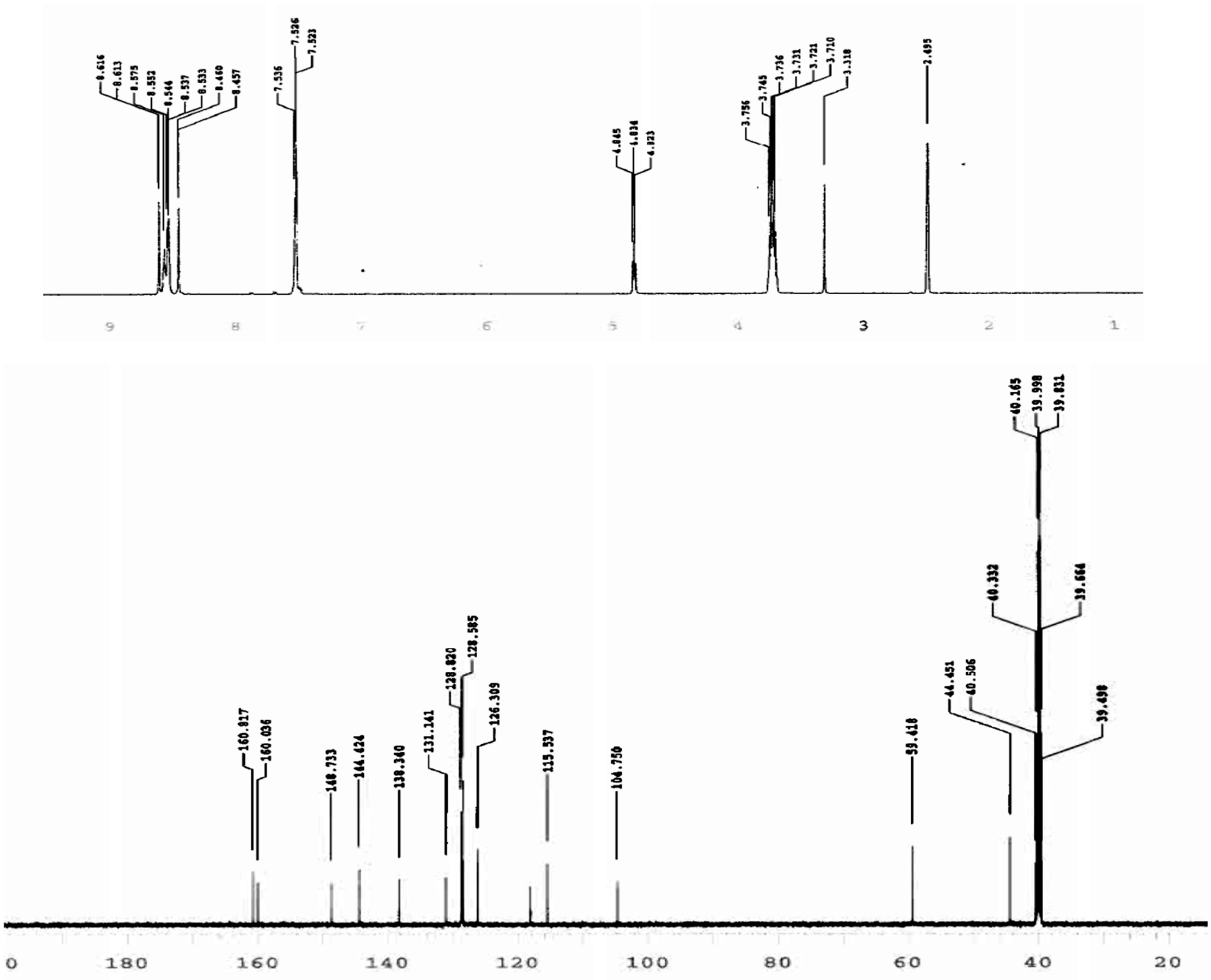

Figure S24. ¹H and ¹³C-NMR spectra of **2a** in DMSO-d_6 at 500 and 125 MHz, respectively.

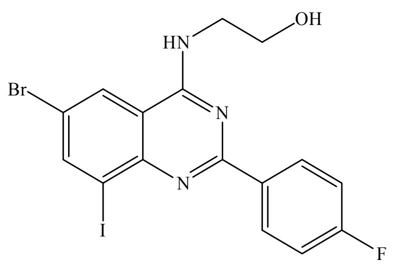

2b

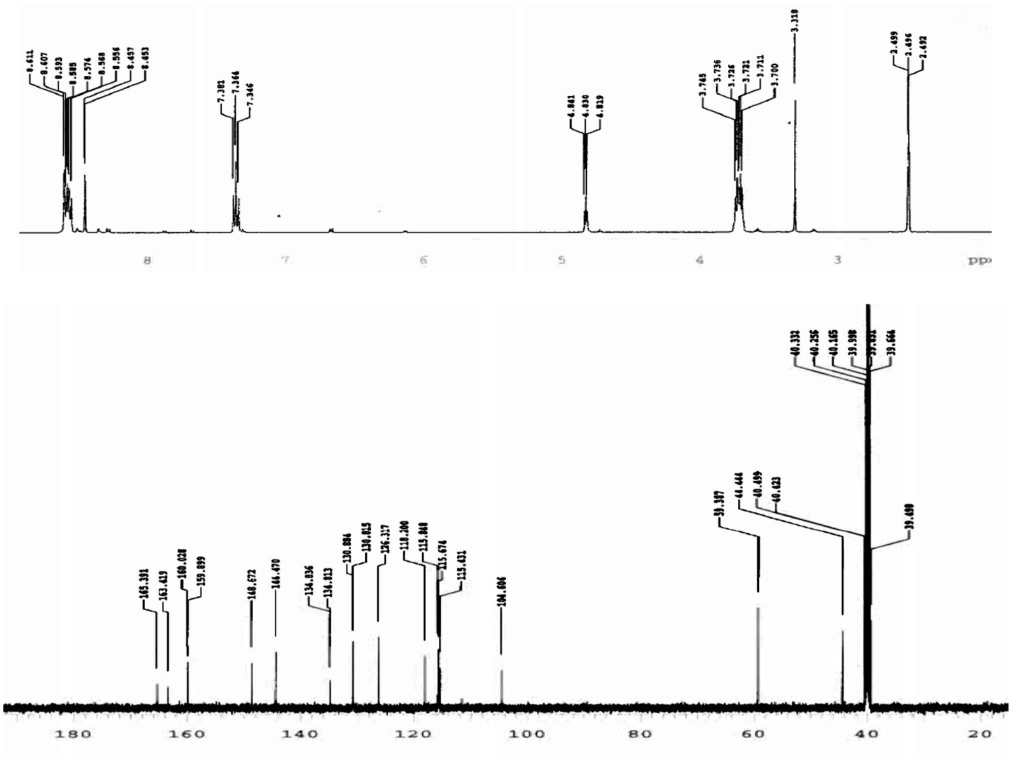

Figure S25. ^{1}H and ^{13}C-NMR spectrum of **2b** in DMSO-d_6 at 500 and 125 MHz, respectively.

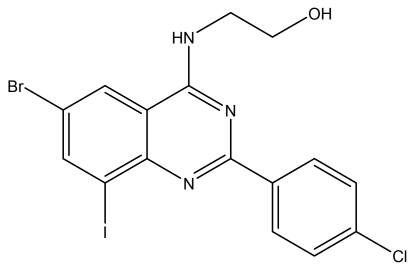

2c

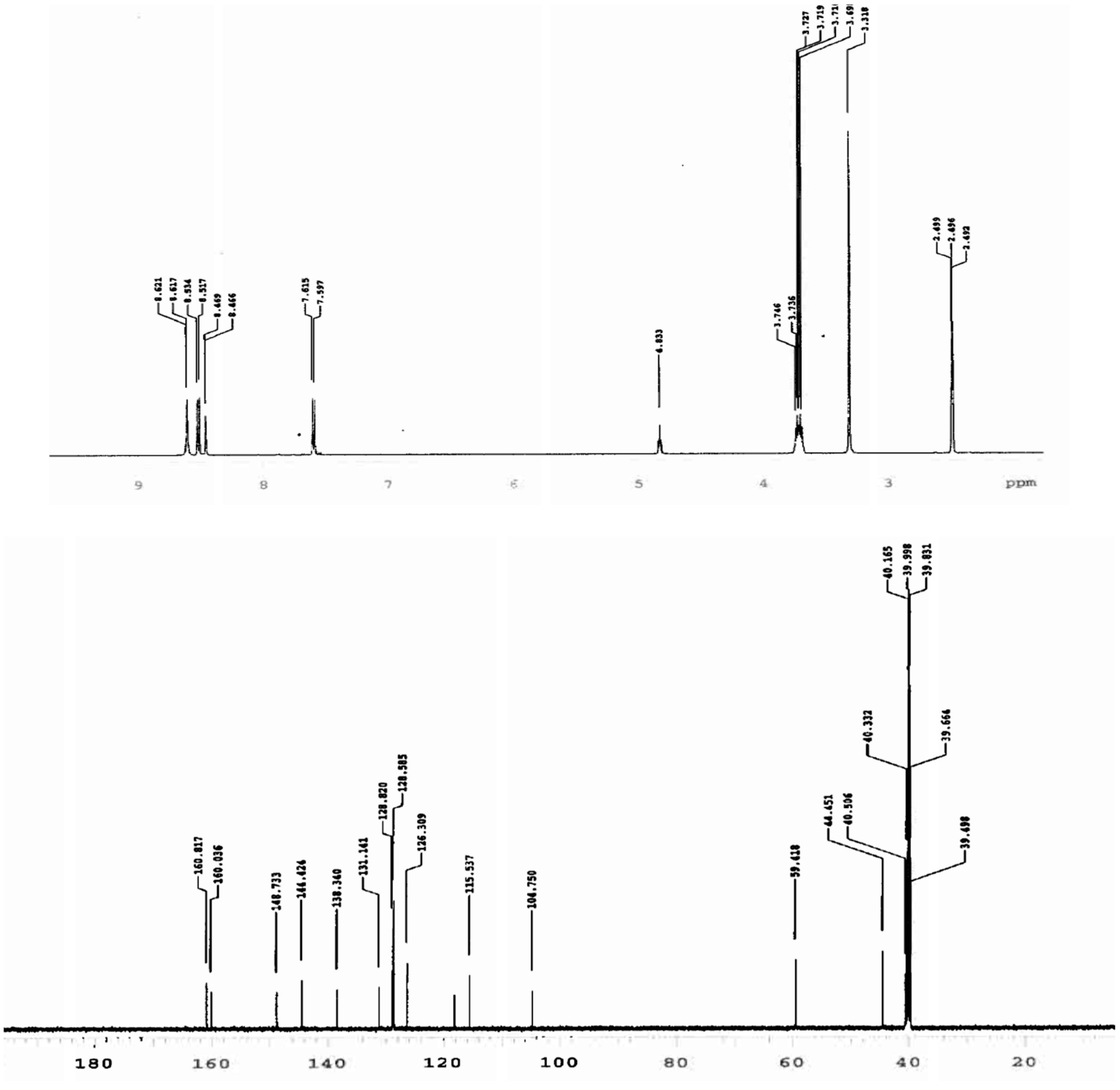

Figure S26.^{1}H and ^{13}C-NMR spectrum of **2c** in DMSO-d_6 at 500 and 125 MHz, respectively.

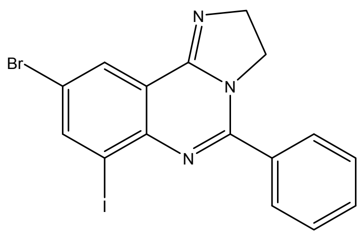

3a

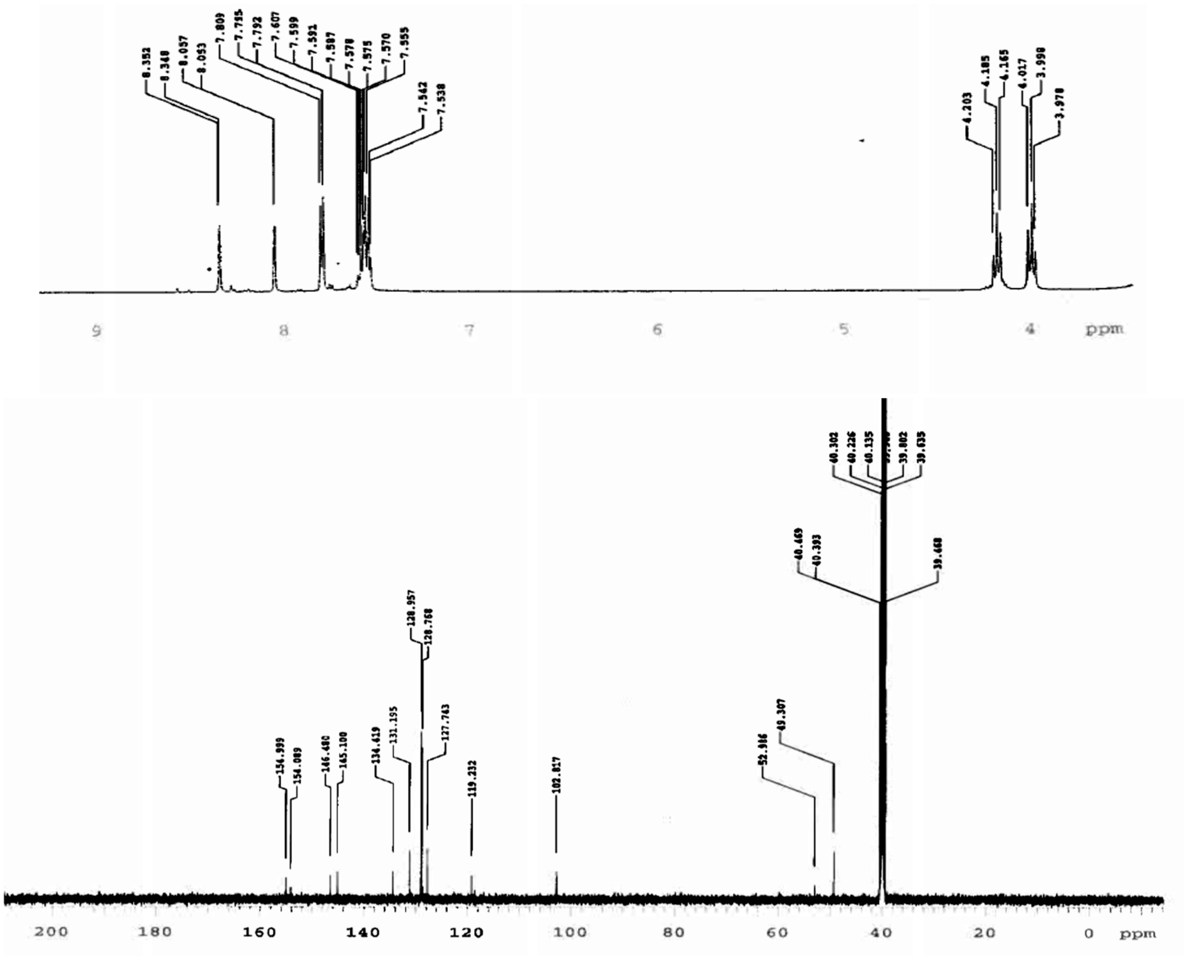

Figure S27. ^{1}H and ^{13}C-NMR spectrum of **3a** in DMSO-d_6 at 500 and 125 MHz, respectively.

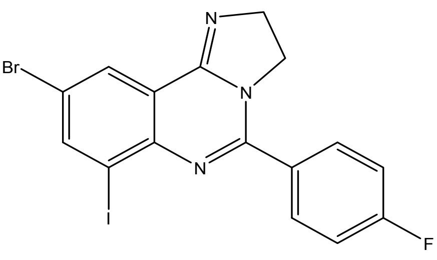

3b

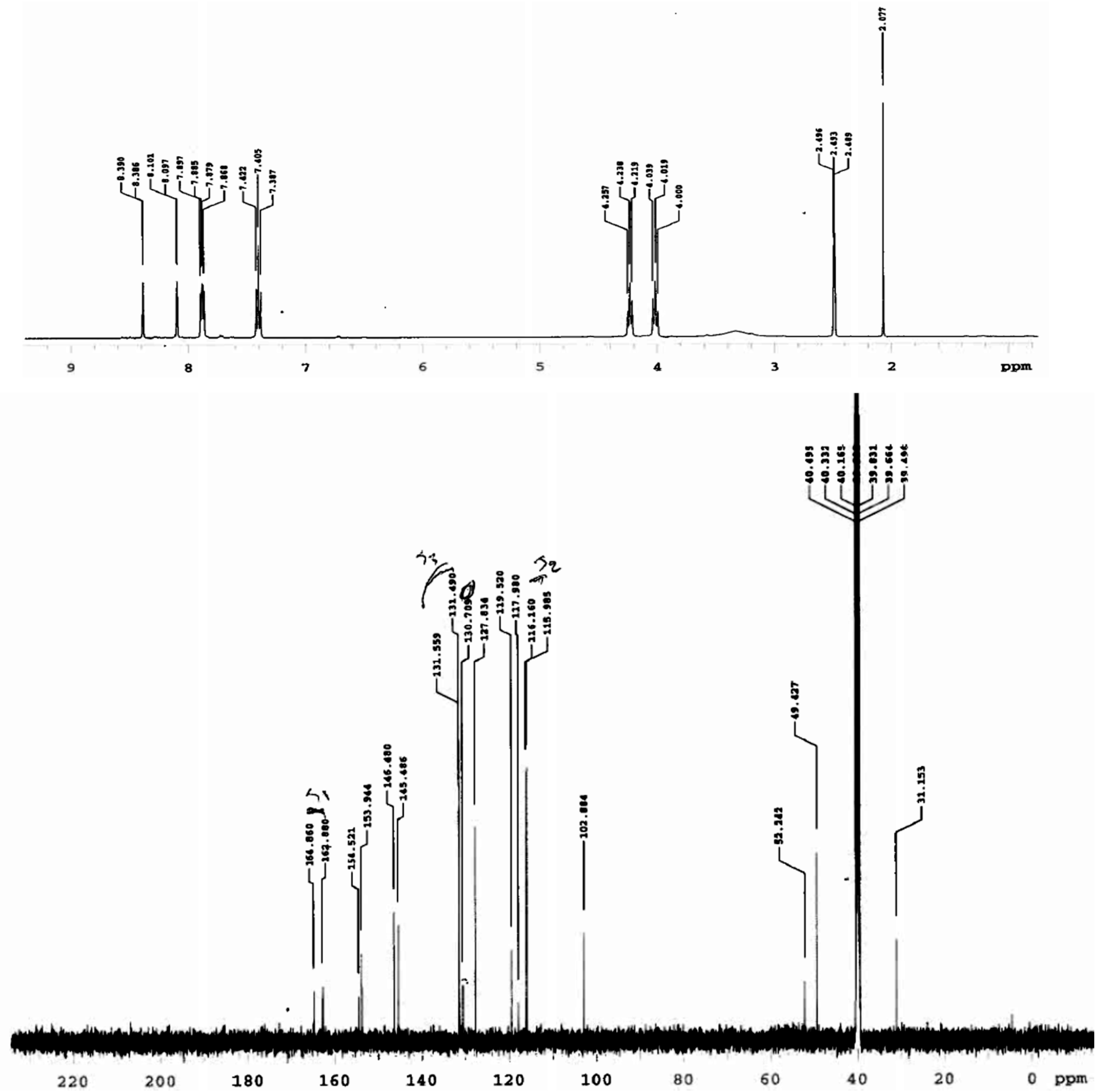

Figure S28. ^{1}H and ^{13}C-NMR spectrum of **3b** in DMSO-d_6 at 500 and 125 MHz, respectively.

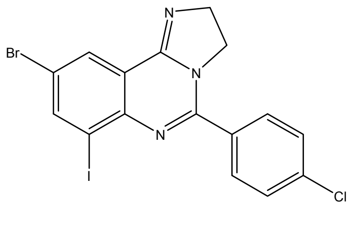

3c

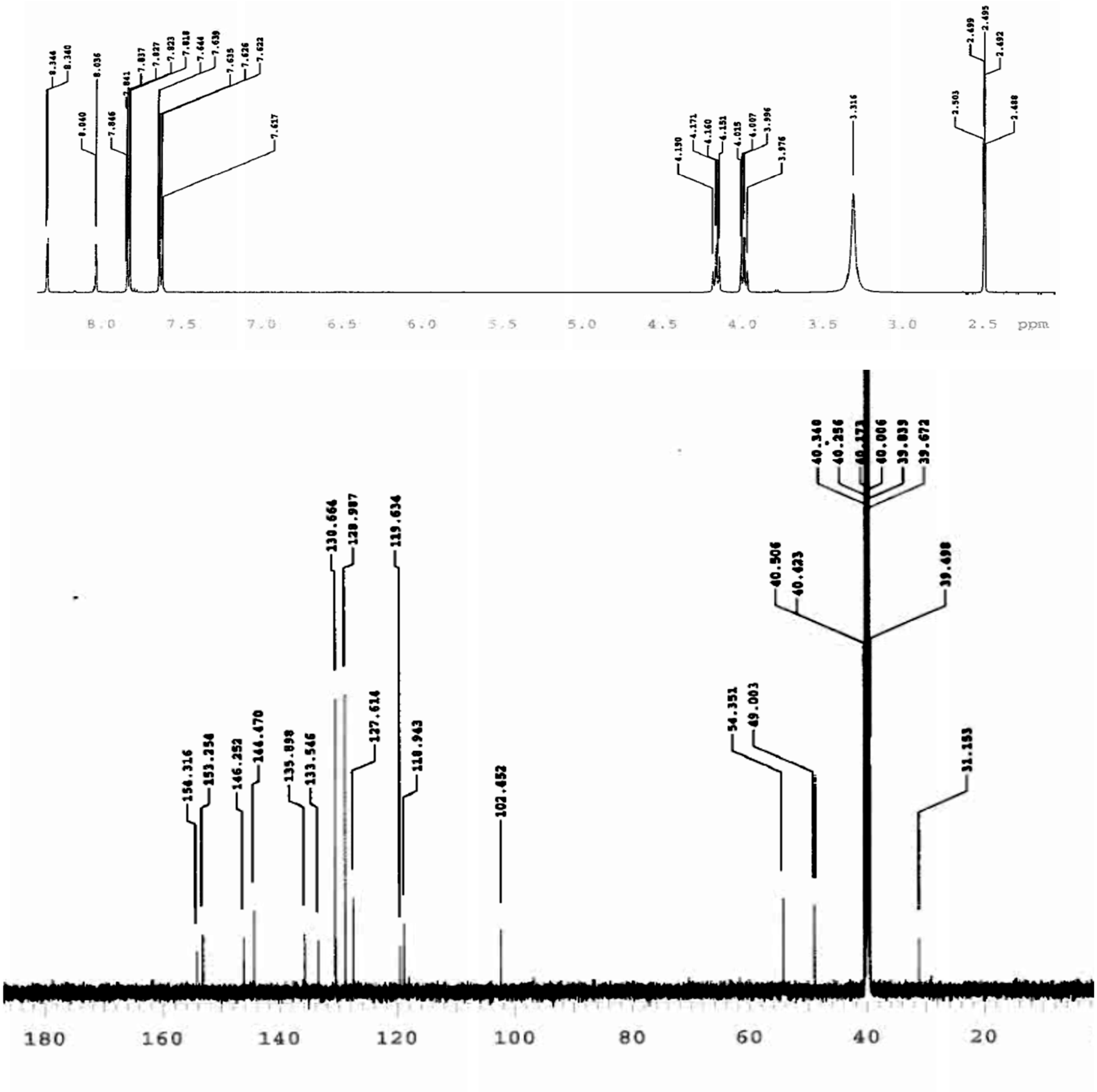

Figure S29. ^{1}H and ^{13}C-NMR spectrum of **3c** in DMSO-d_6 at 500 and 125 MHz, respectively.

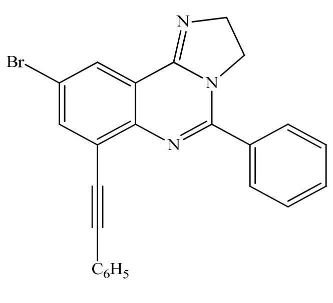

4a

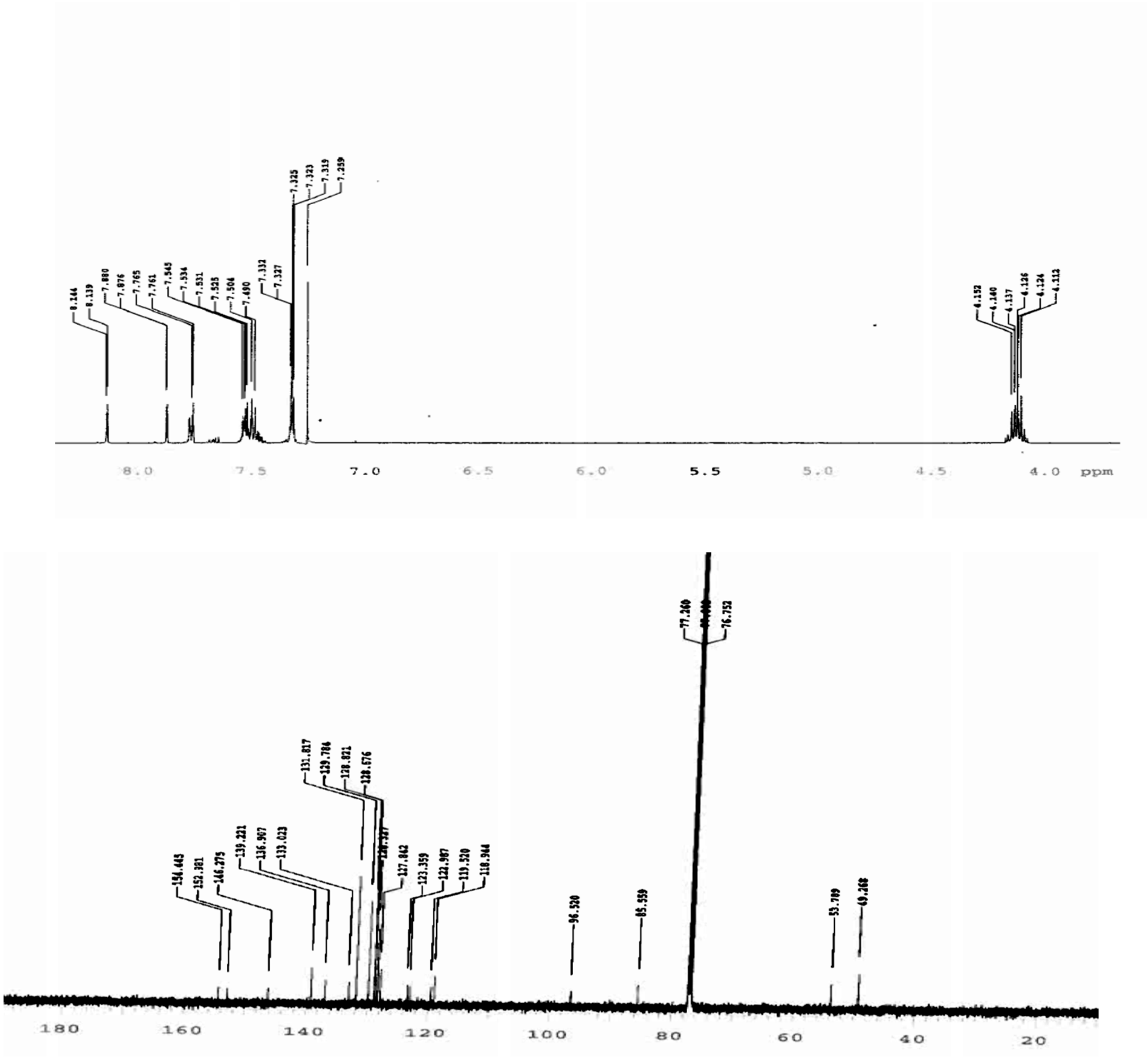

Figure S30. ^{1}H and ^{13}C-NMR spectrum of **4a** in $CDCl_3$ at 500 and 125 MHz, respectively.

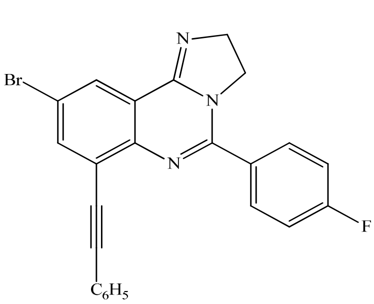

4b

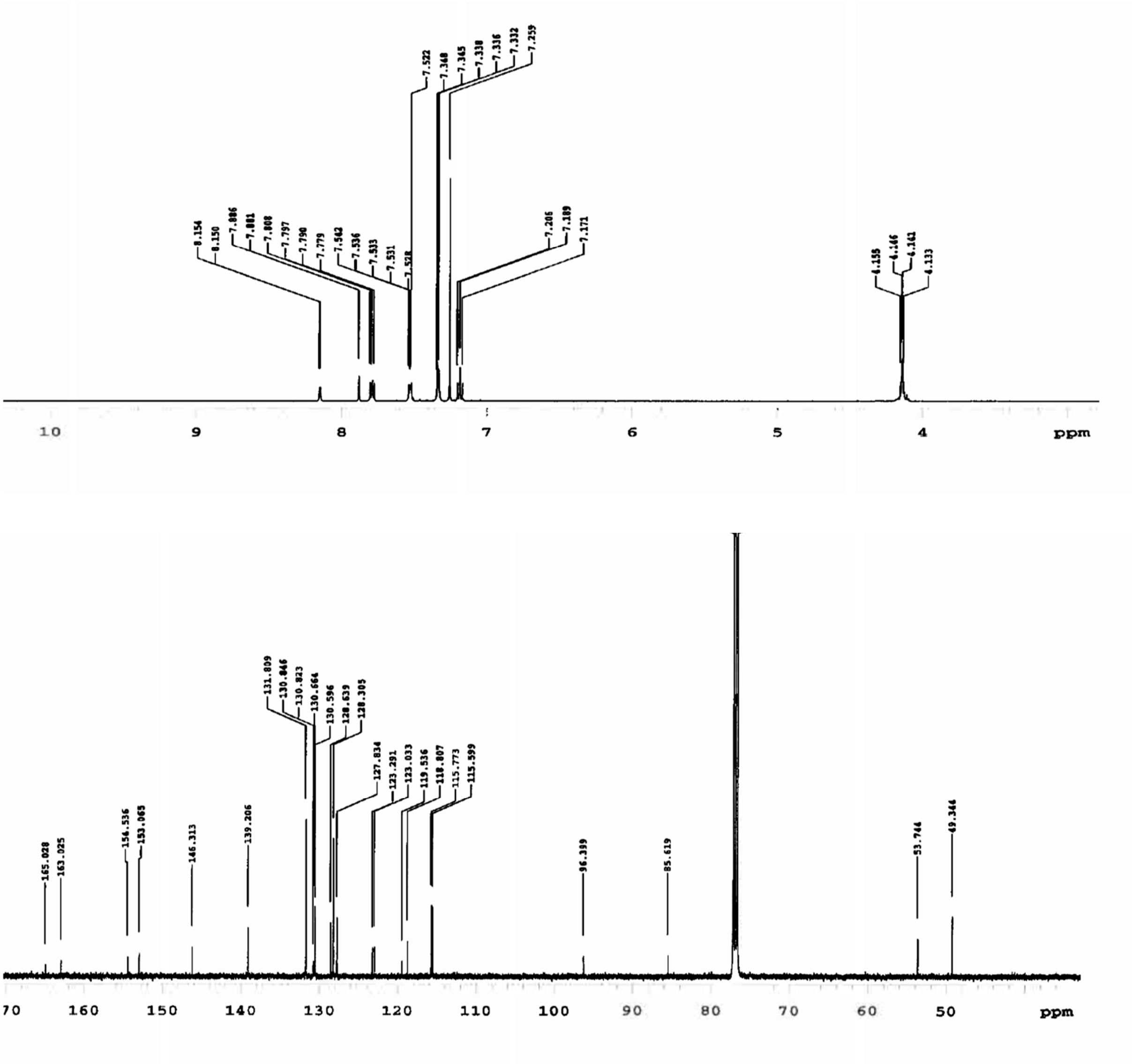

Figure S31. ^{1}H and ^{13}C-NMR spectrum of **4b** in $CDCl_3$ at 500 and 125 MHz, respectively.

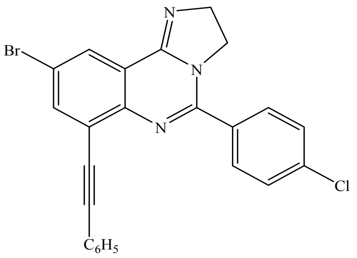

4c

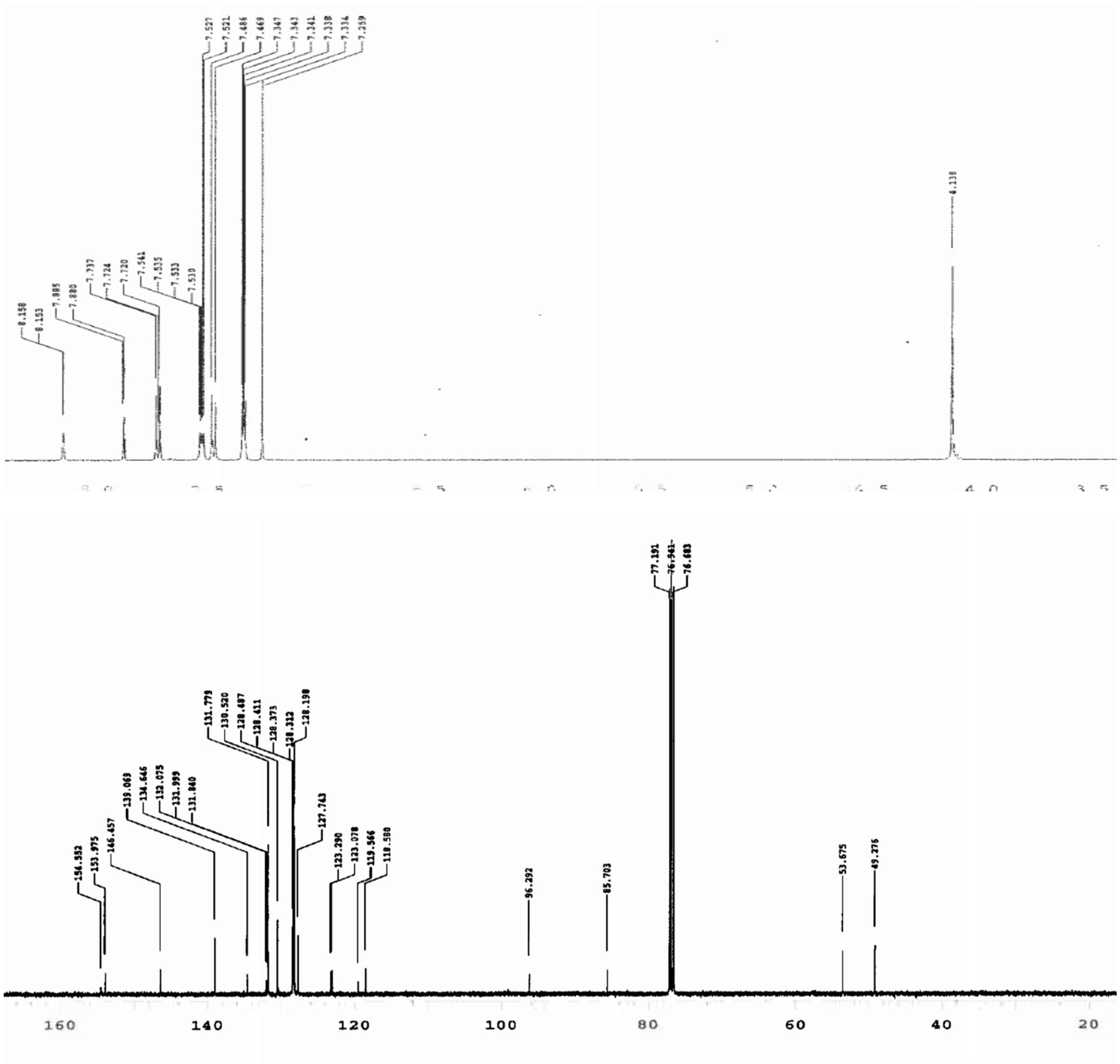

Figure S32. ^{1}H and ^{13}C-NMR spectrum of **4c** in $CDCl_3$ at 500 and 125 MHz, respectively.

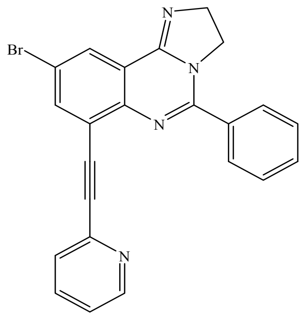

4d

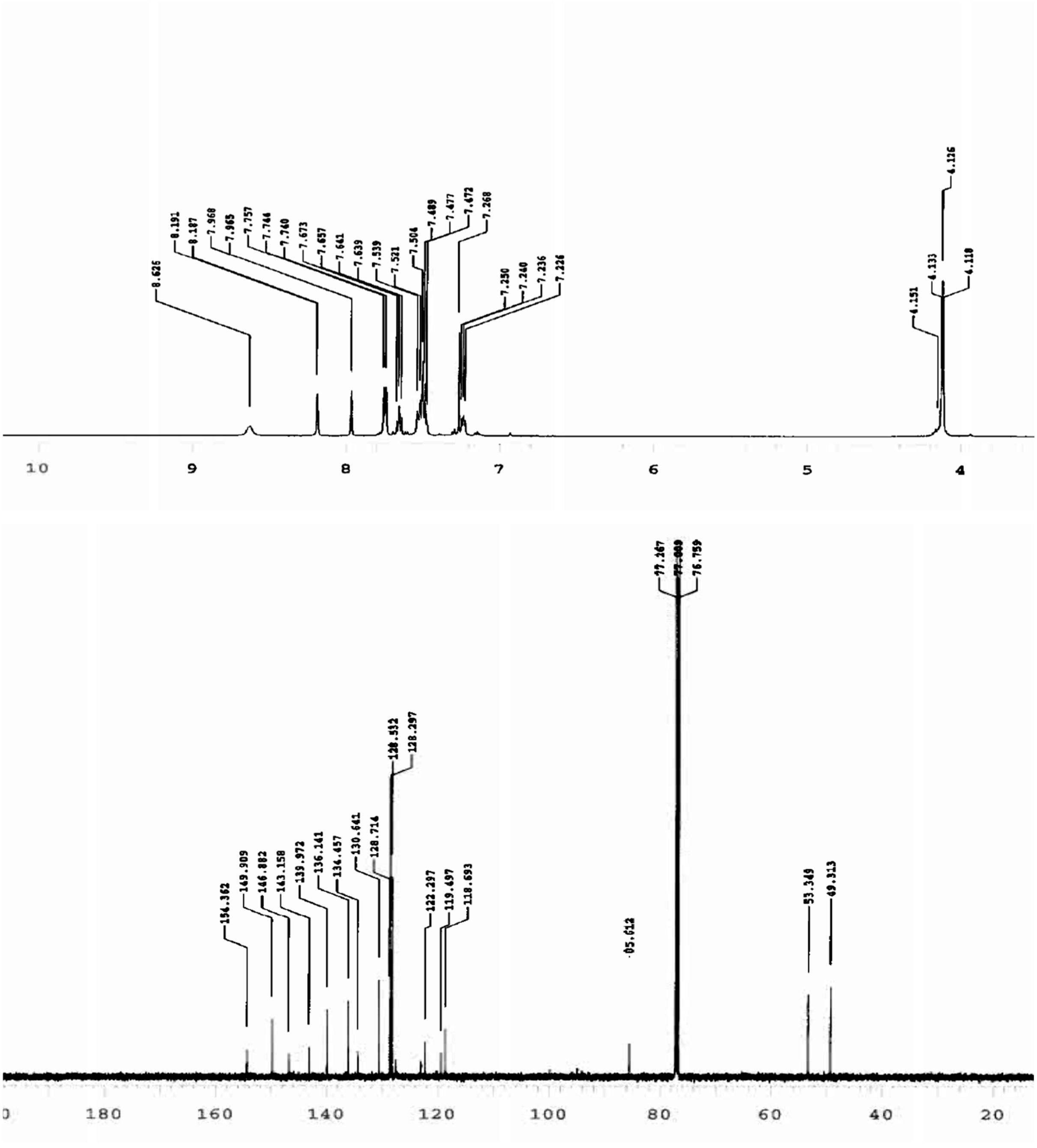

Figure S33. ^{1}H and ^{13}C-NMR spectrum of **4d** in $CDCl_3$ at 500 and 125 MHz, respectively.

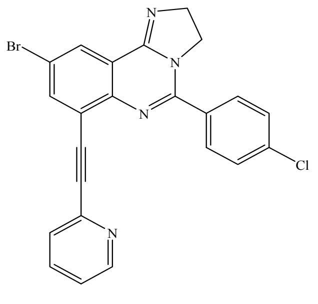

4f

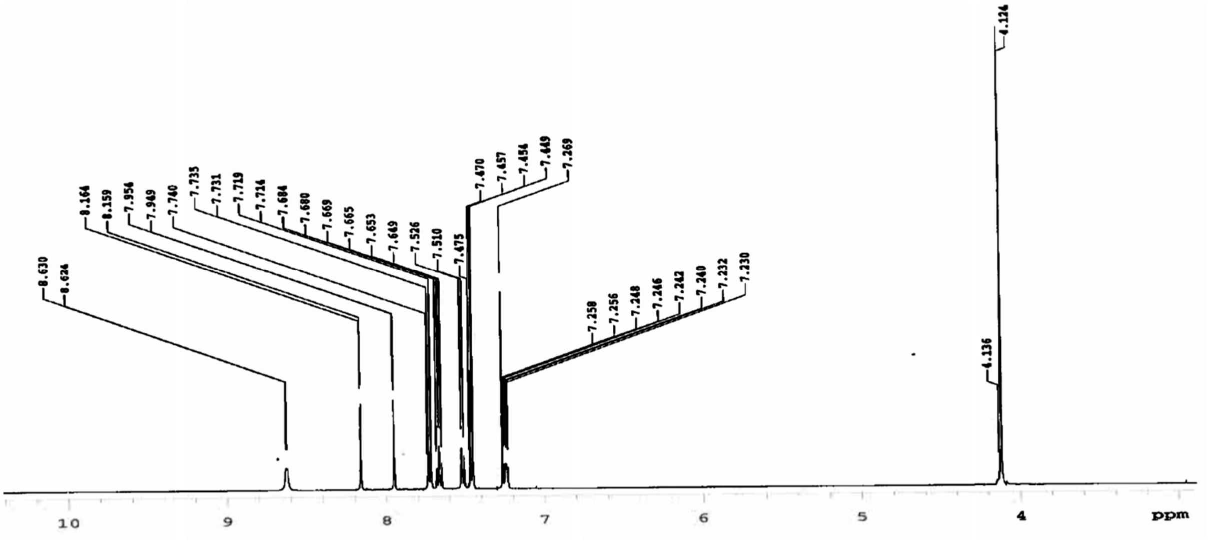

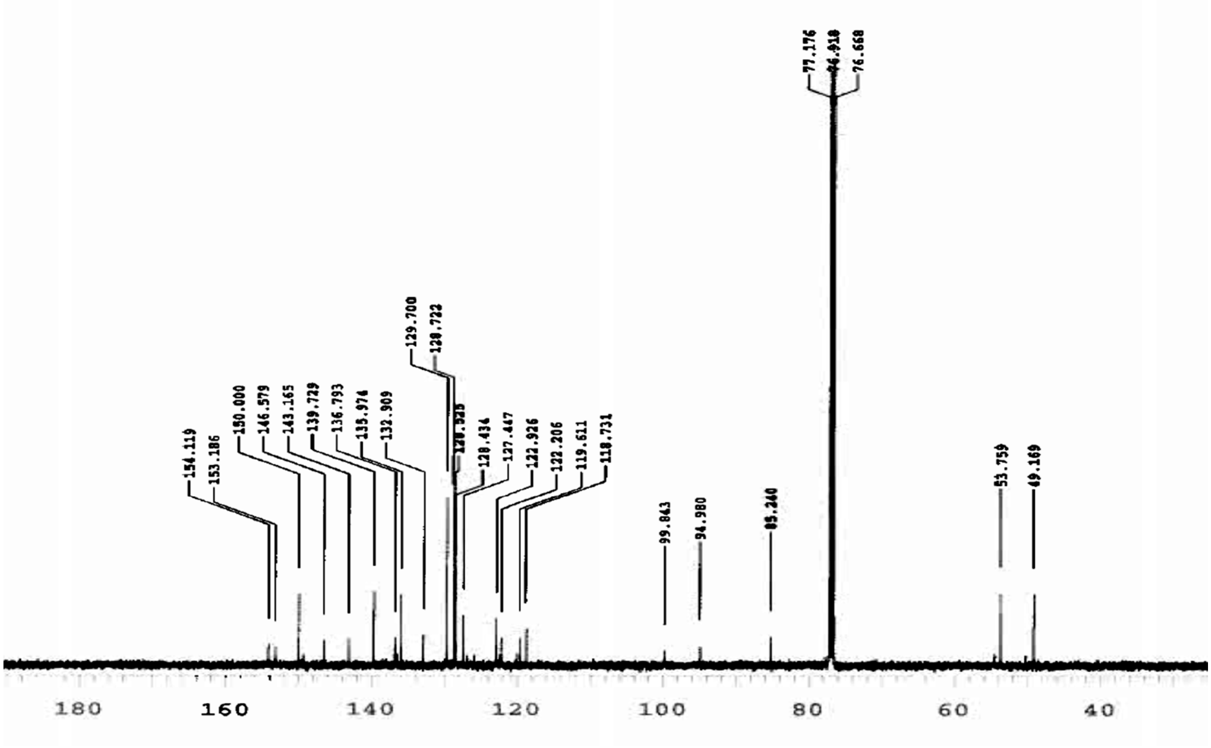

Figure S34. ^{1}H and ^{13}C-NMR spectrum of **4f** in $CDCl_3$ at 500 and 125 MHz, respectively.

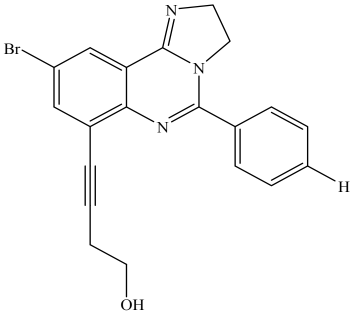

4g

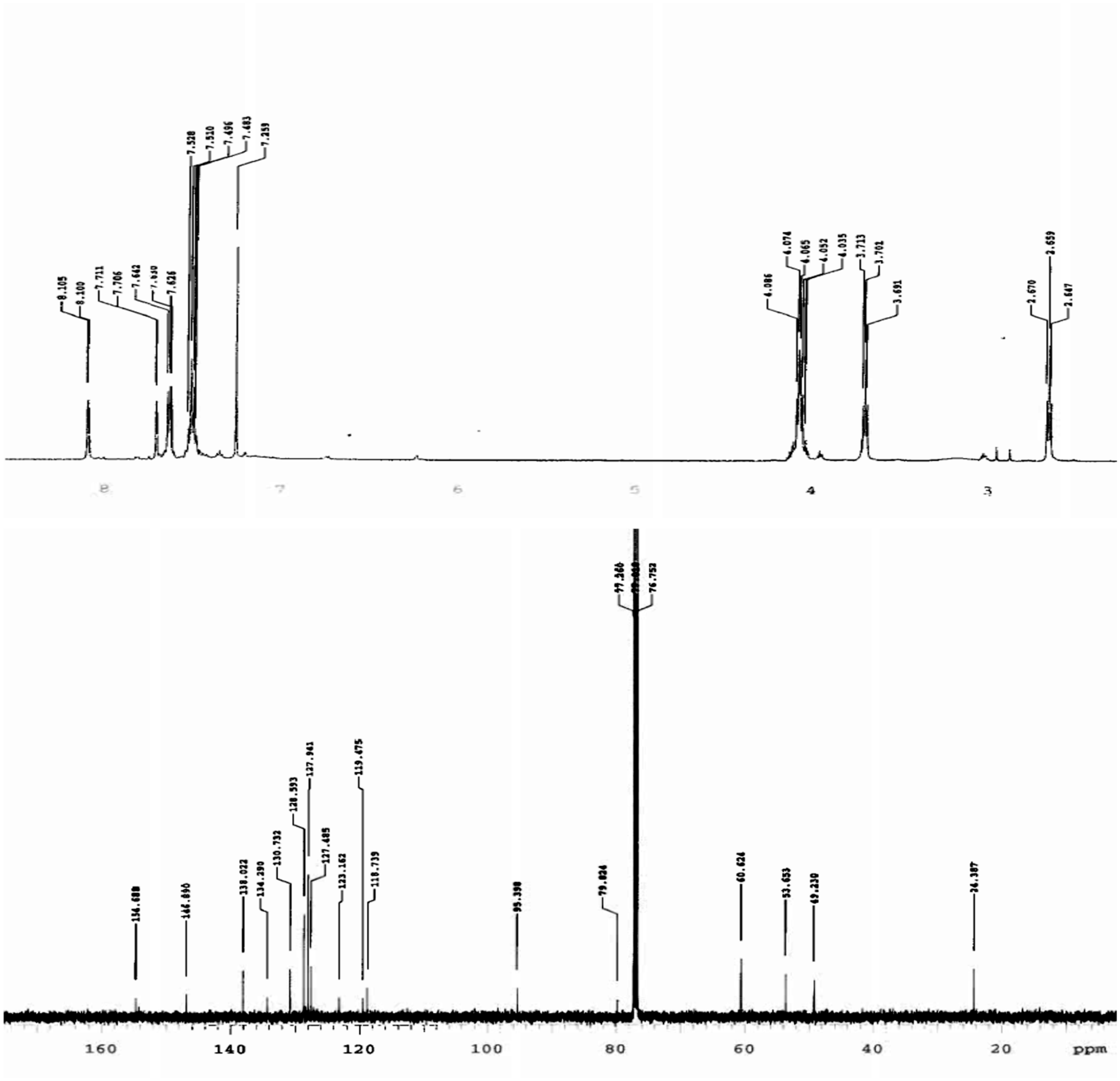

Figure S35. ¹H and ¹³C-NMR spectrum of **4g** in $CDCl_3$ at 500 and 125 MHz, respectively.

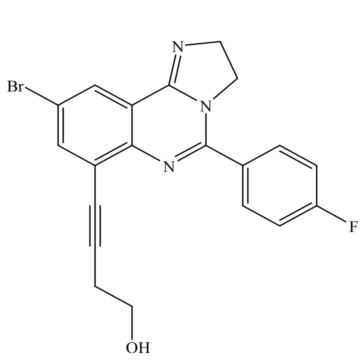

4h

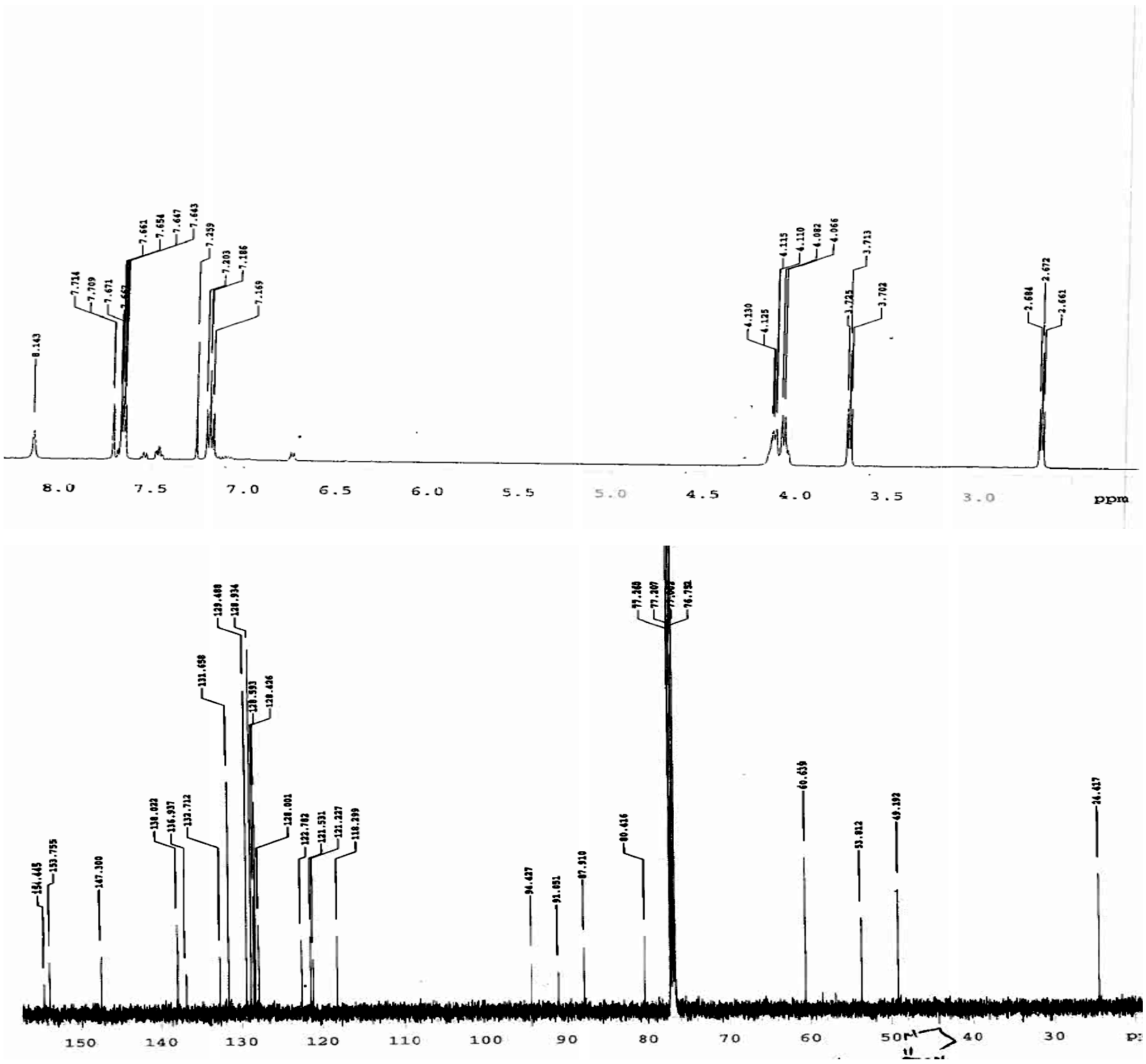

Figure S36. ^{1}H and ^{13}C-NMR spectrum of **4h** in $CDCl_3$ at 500 and 125 MHz, respectively.

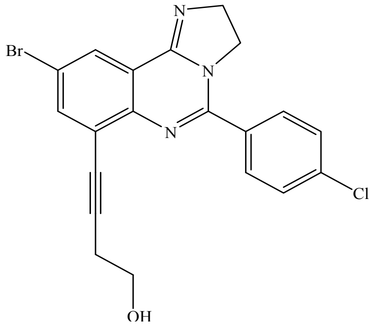

4i

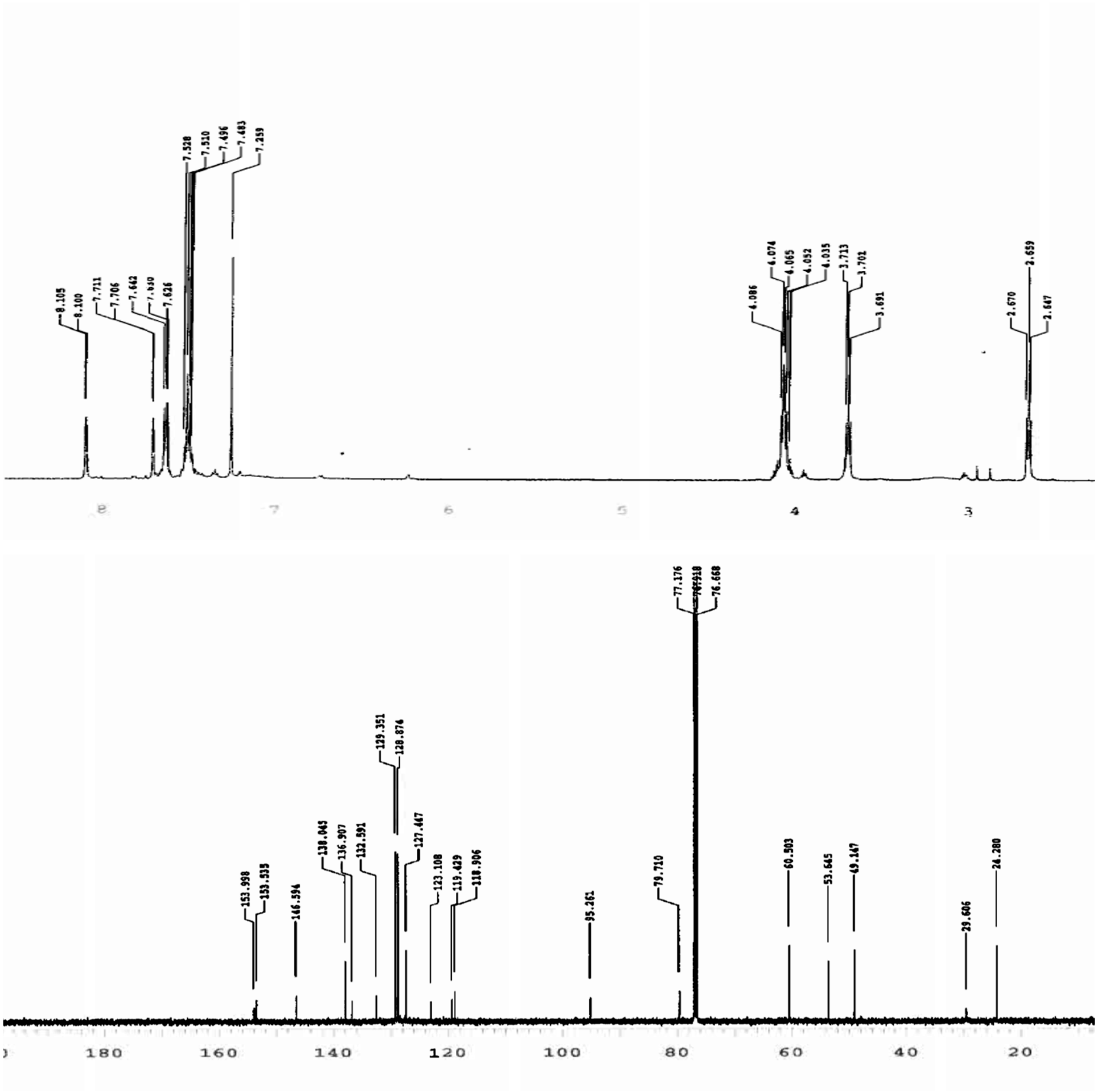

Figure S37. ^{1}H and ^{13}C-NMR spectrum of **4i** in $CDCl_3$ at 500 and 125 MHz, respectively.

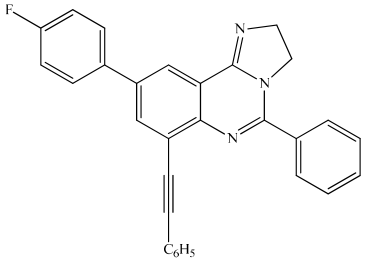

5a

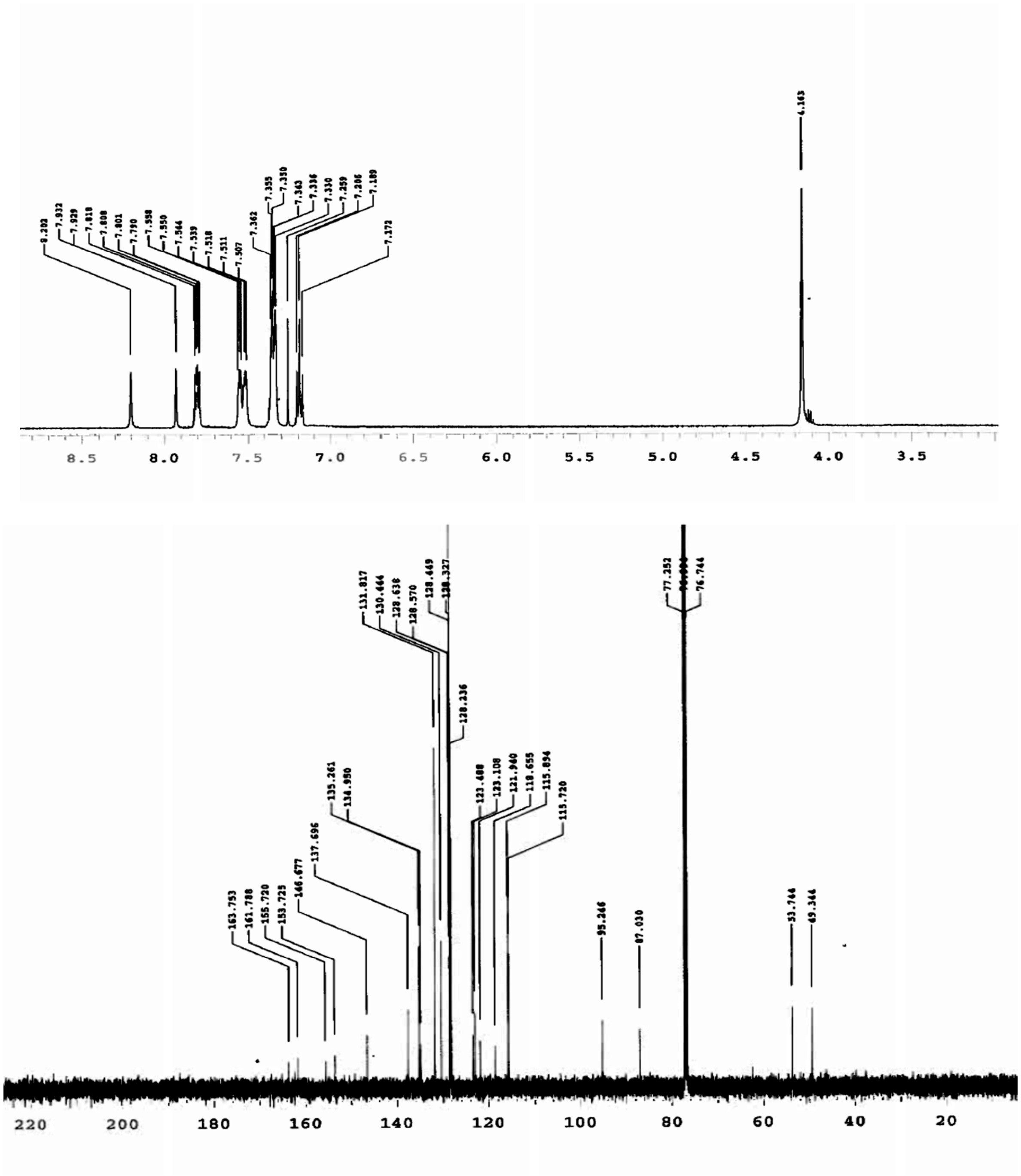

Figure S38. ^{1}H and ^{13}C-NMR spectrum of **5a** in $CDCl_3$ at 500 and 125 MHz, respectively.

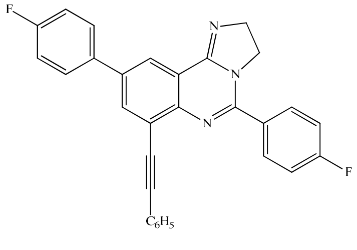

5b

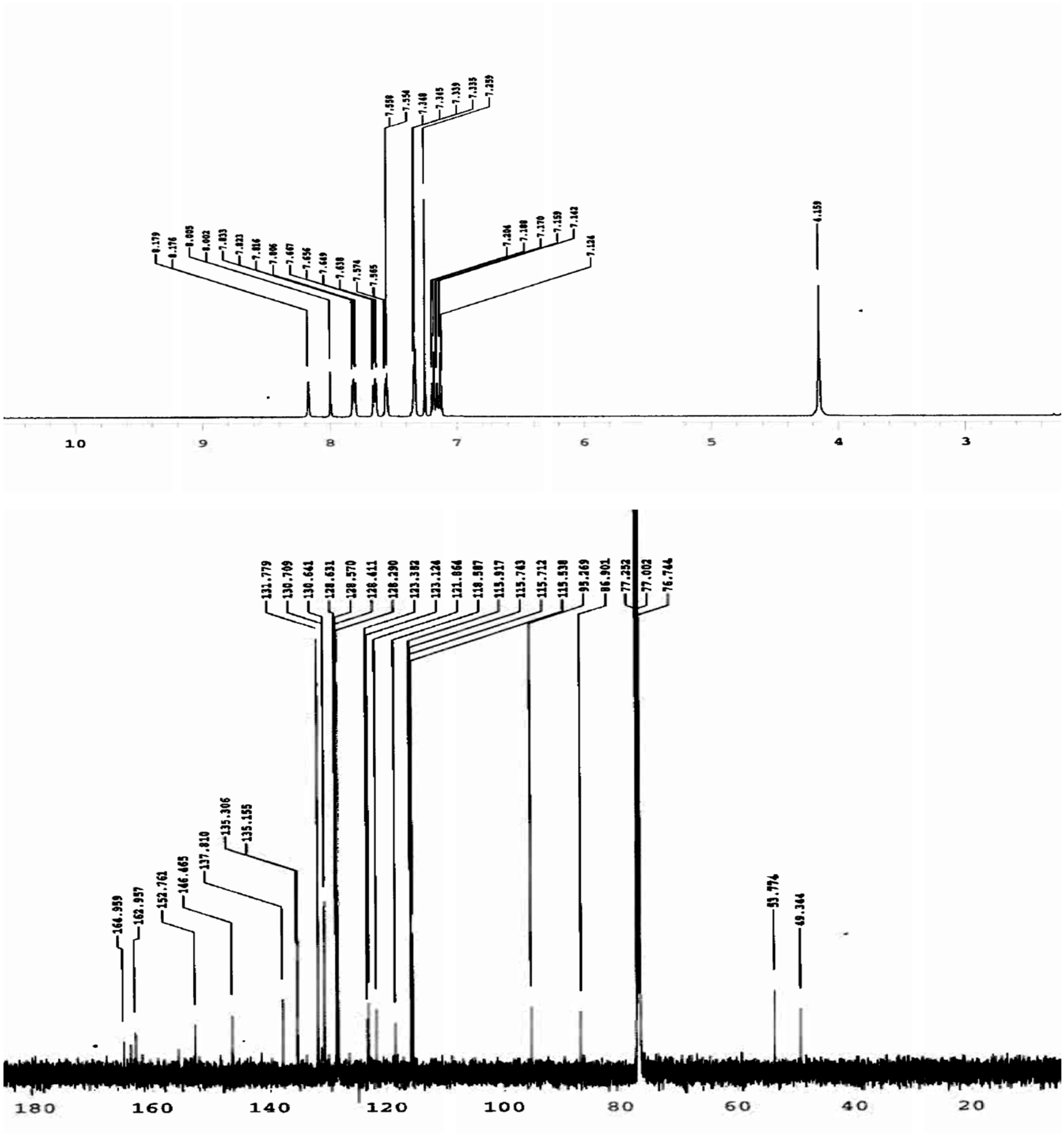

Figure S39. ^1H and ^{13}C-NMR spectrum of **5b** in $CDCl_3$ at 500 and 125 MHz, respectively.

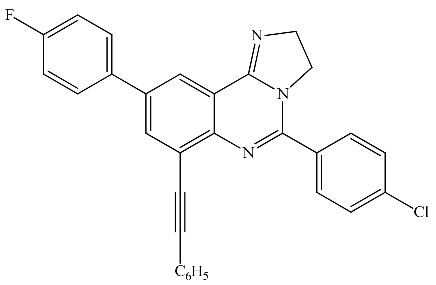

5c

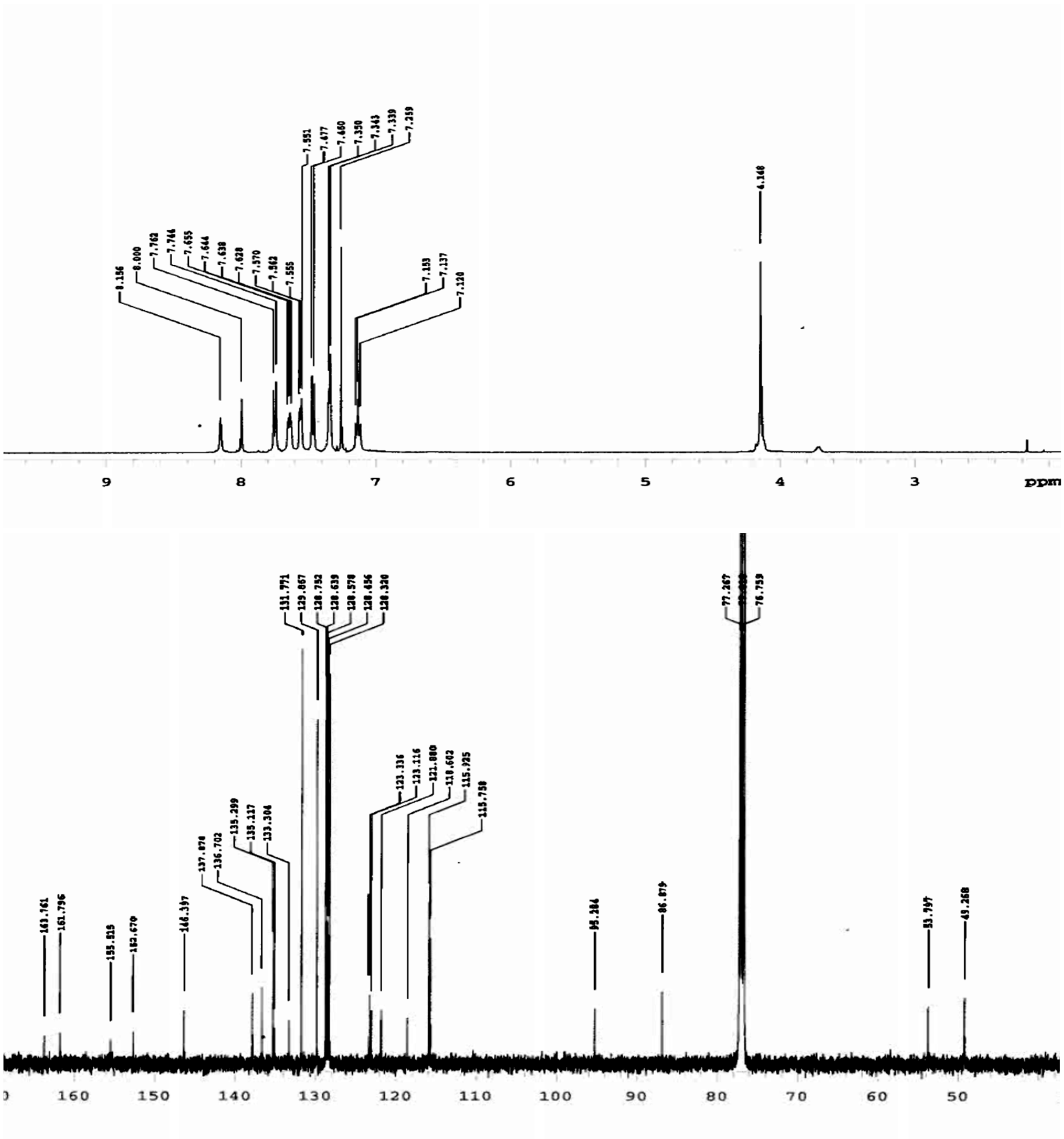

Figure S40. ^{1}H and ^{13}C-NMR spectrum of **5c** in $CDCl_3$ at 500 and 125 MHz, respectively.

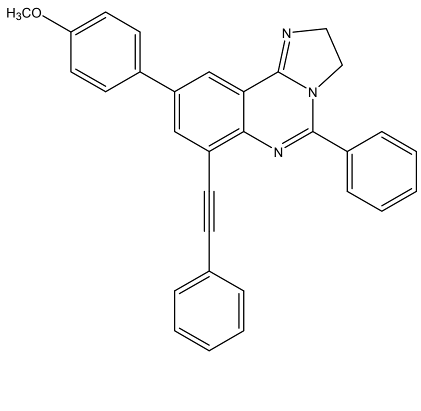

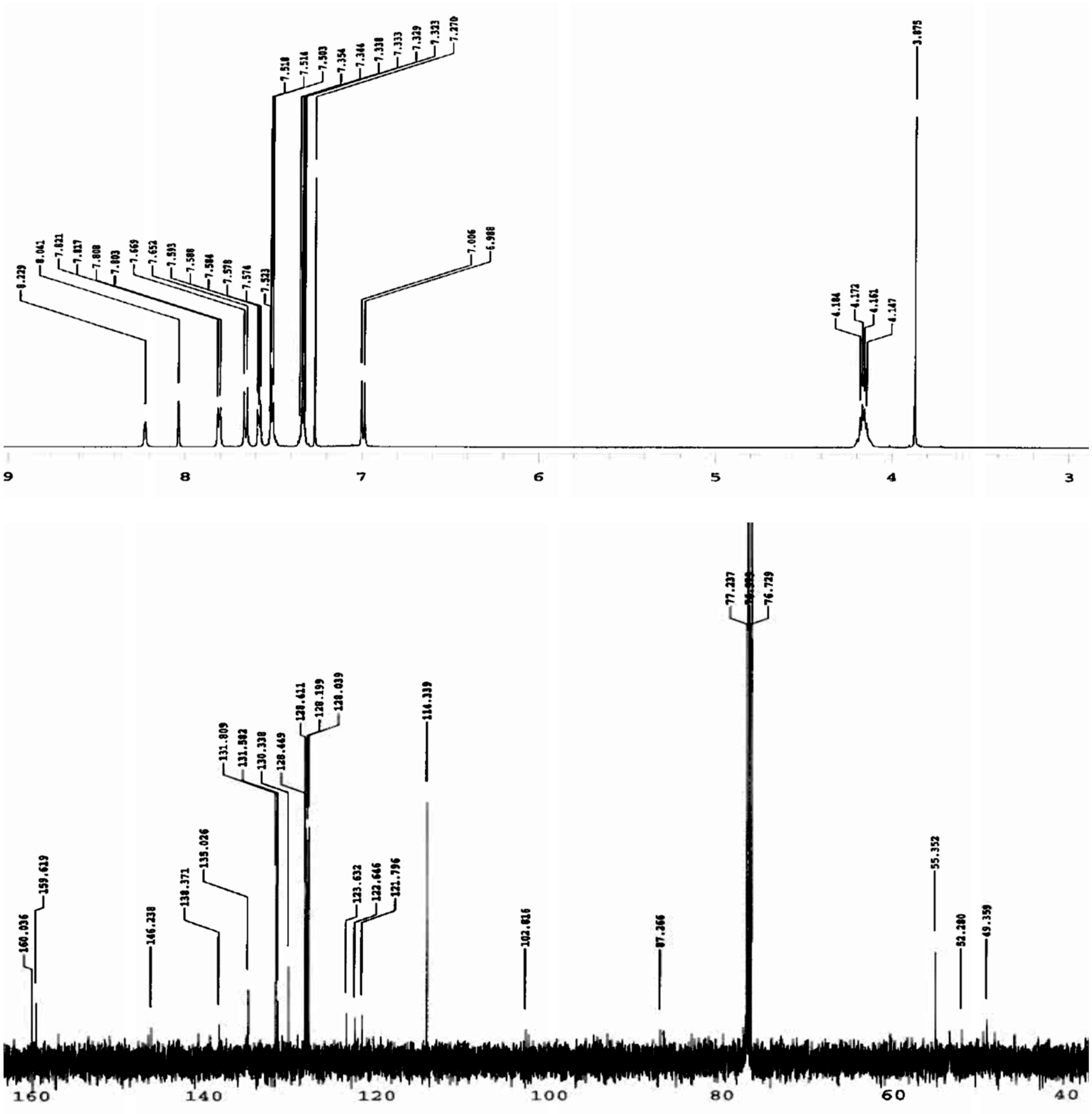

Figure 41.^{1}H and ^{13}C-NMR spectrum of **5d** in $CDCl_3$ at 500 and 125 MHz, respectively.

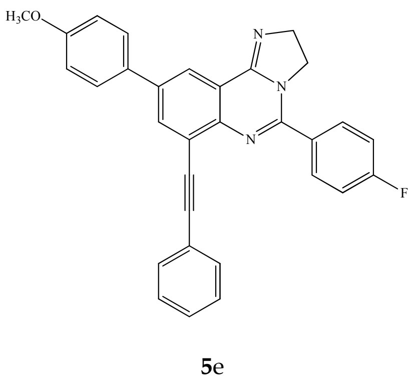

5e

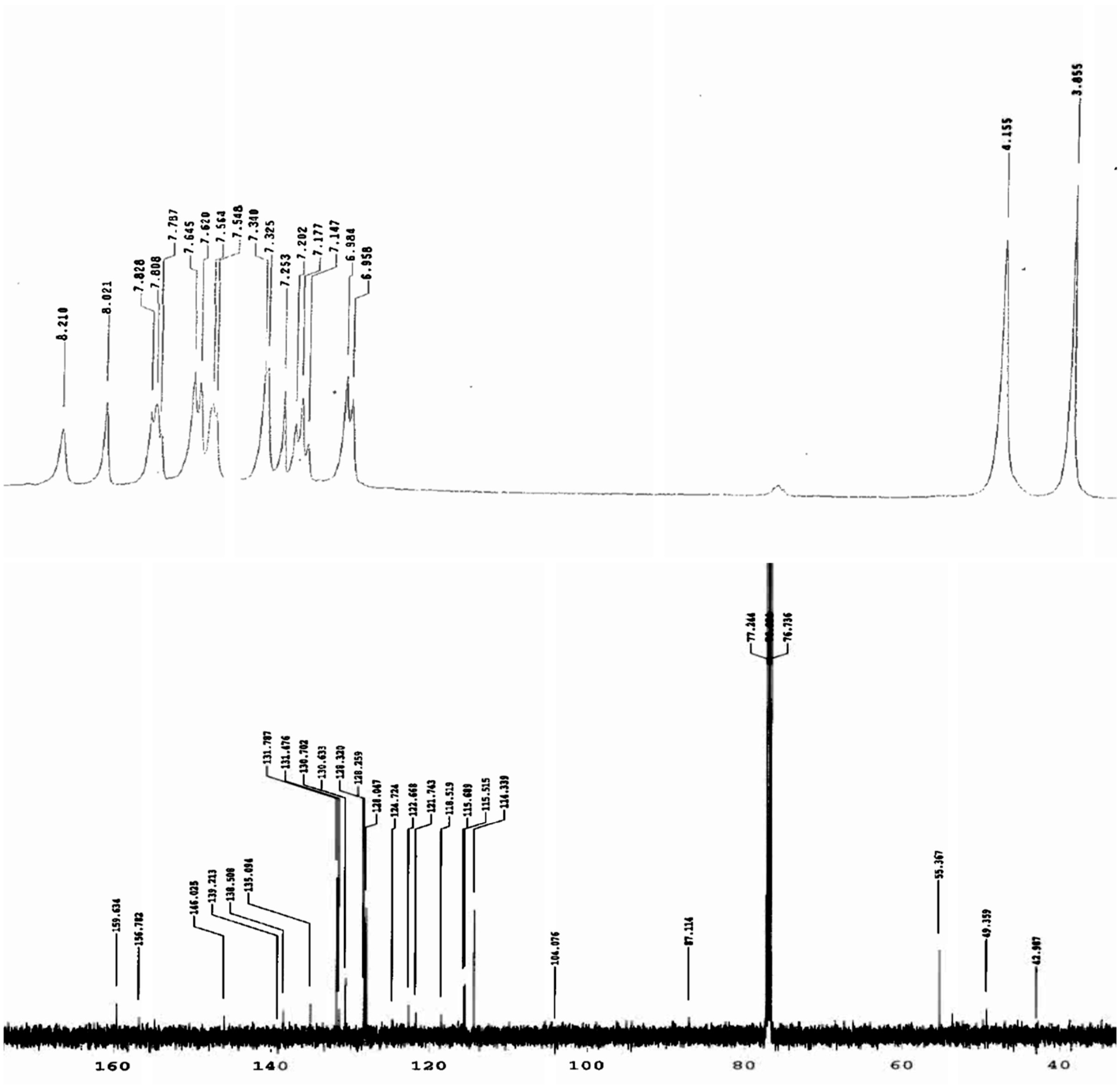

Figure S42.^{1}H and ^{13}C-NMR spectrum of **5e** in $CDCl_3$ at 500 and 125 MHz, respectively.

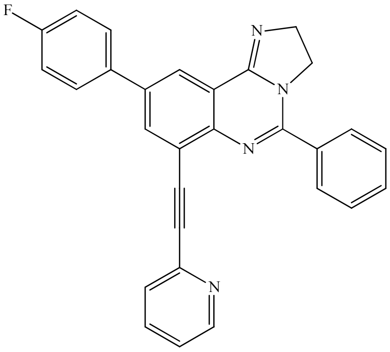

5f

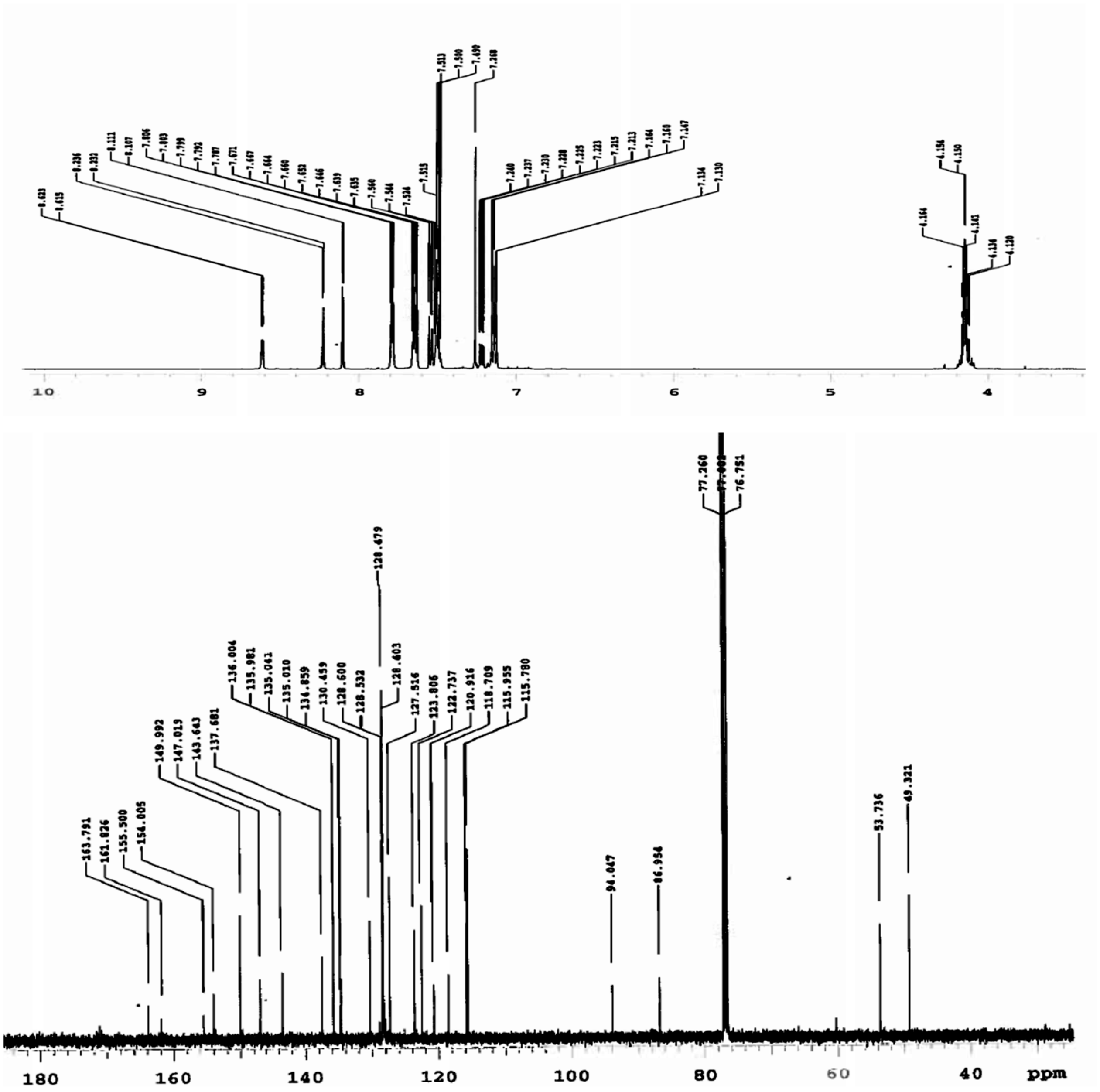

Figure S43.^{1}H and ^{13}C-NMR spectrum of **5f** in $CDCl_3$ at 500 and 125 MHz, respectively.

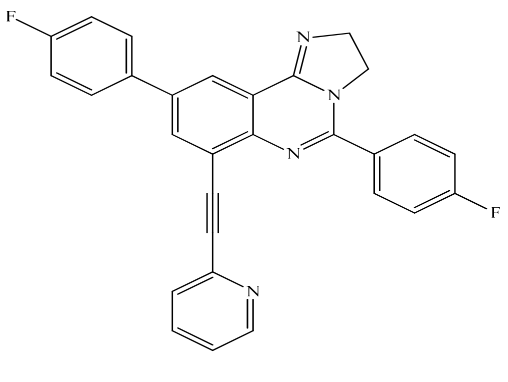

5g

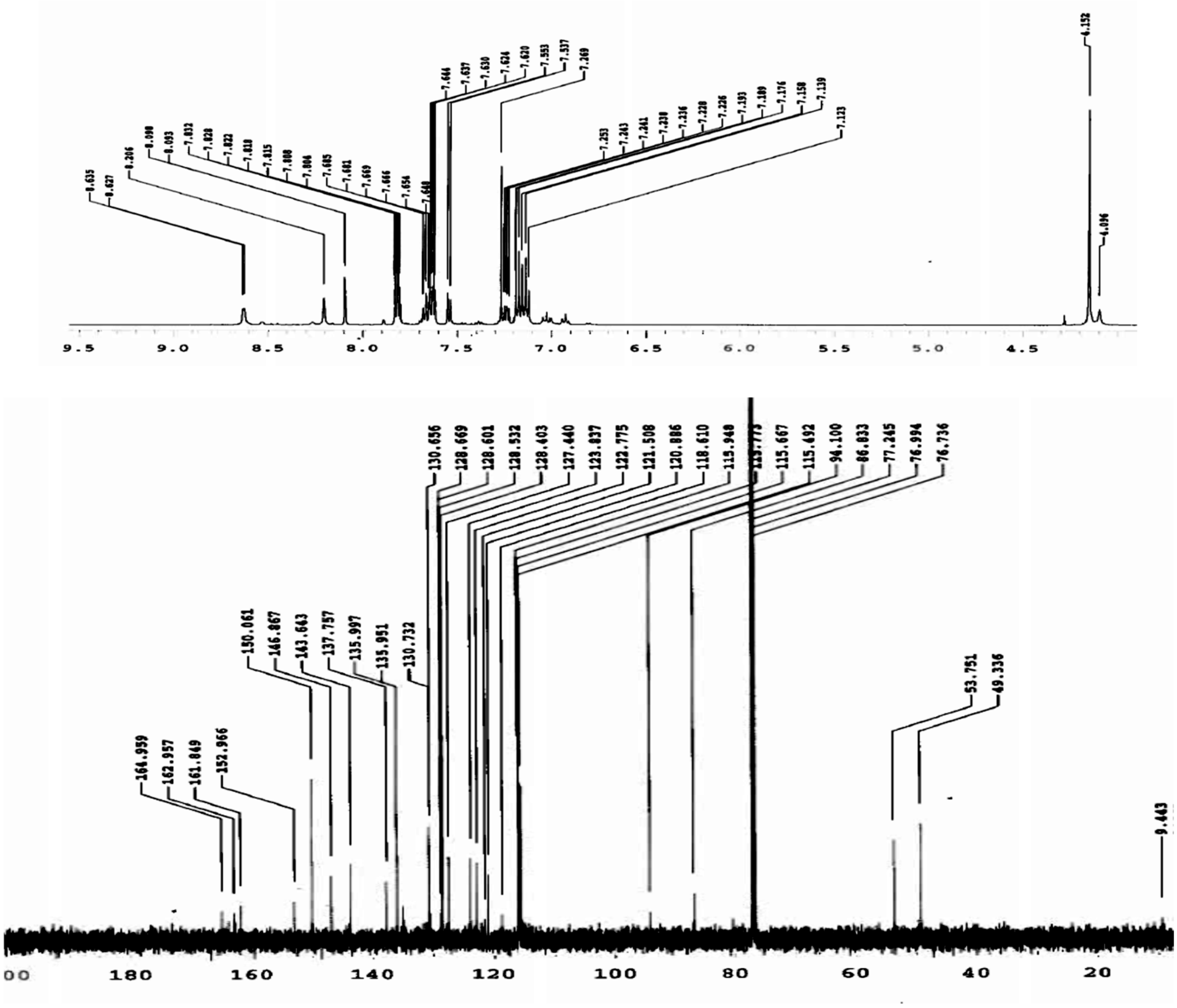

Figure S44.^{1}H and ^{13}C-NMR spectrum of **5g** in $CDCl_3$ at 500 and 125 MHz, respectively.

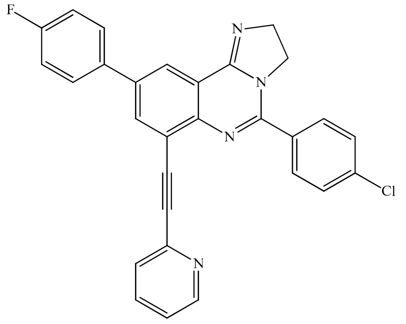

5h

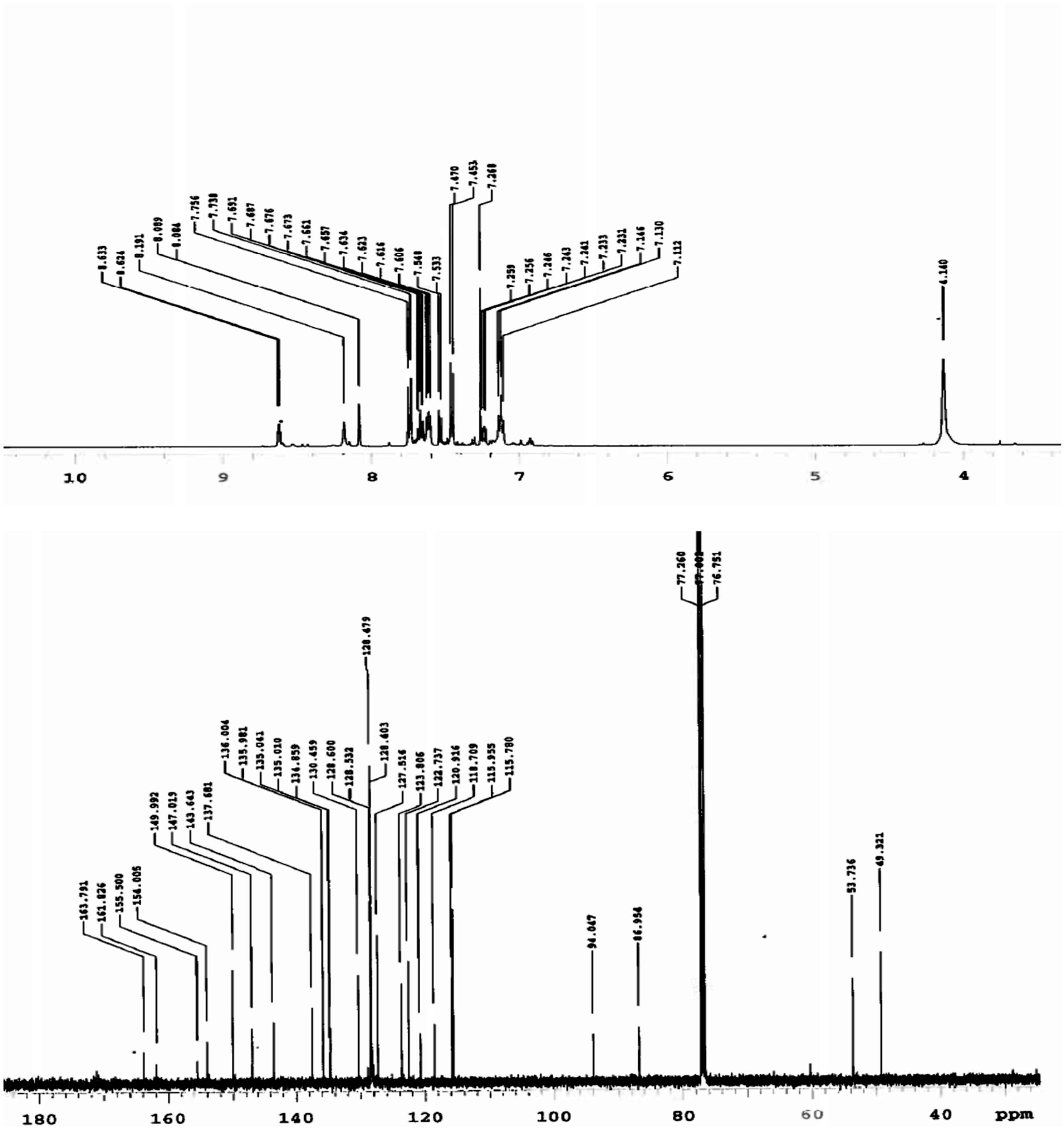

Figure S45.^{1}H and ^{13}C-NMR spectrum of **5h** in $CDCl_3$ at 500 and 125 MHz, respectively.

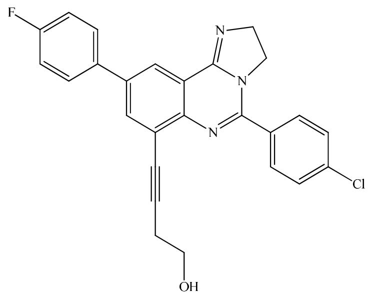

5i

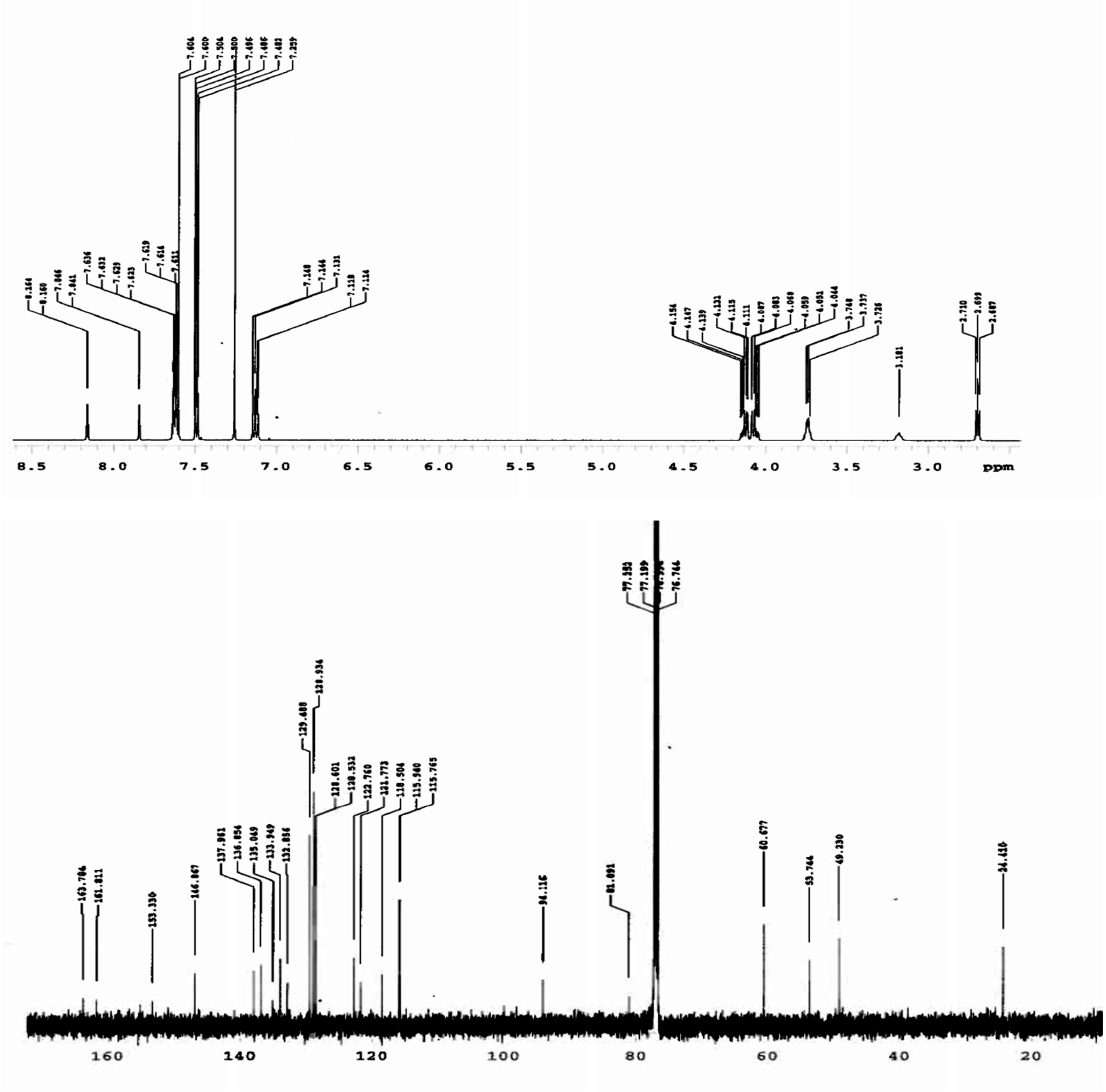

Figure S46.^{1}H and ^{13}C-NMR spectrum of **5i** in $CDCl_3$ at 500 and 125 MHz, respectively.

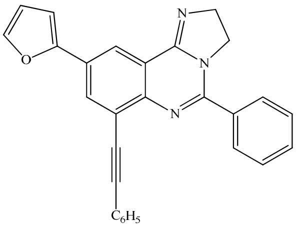

6a

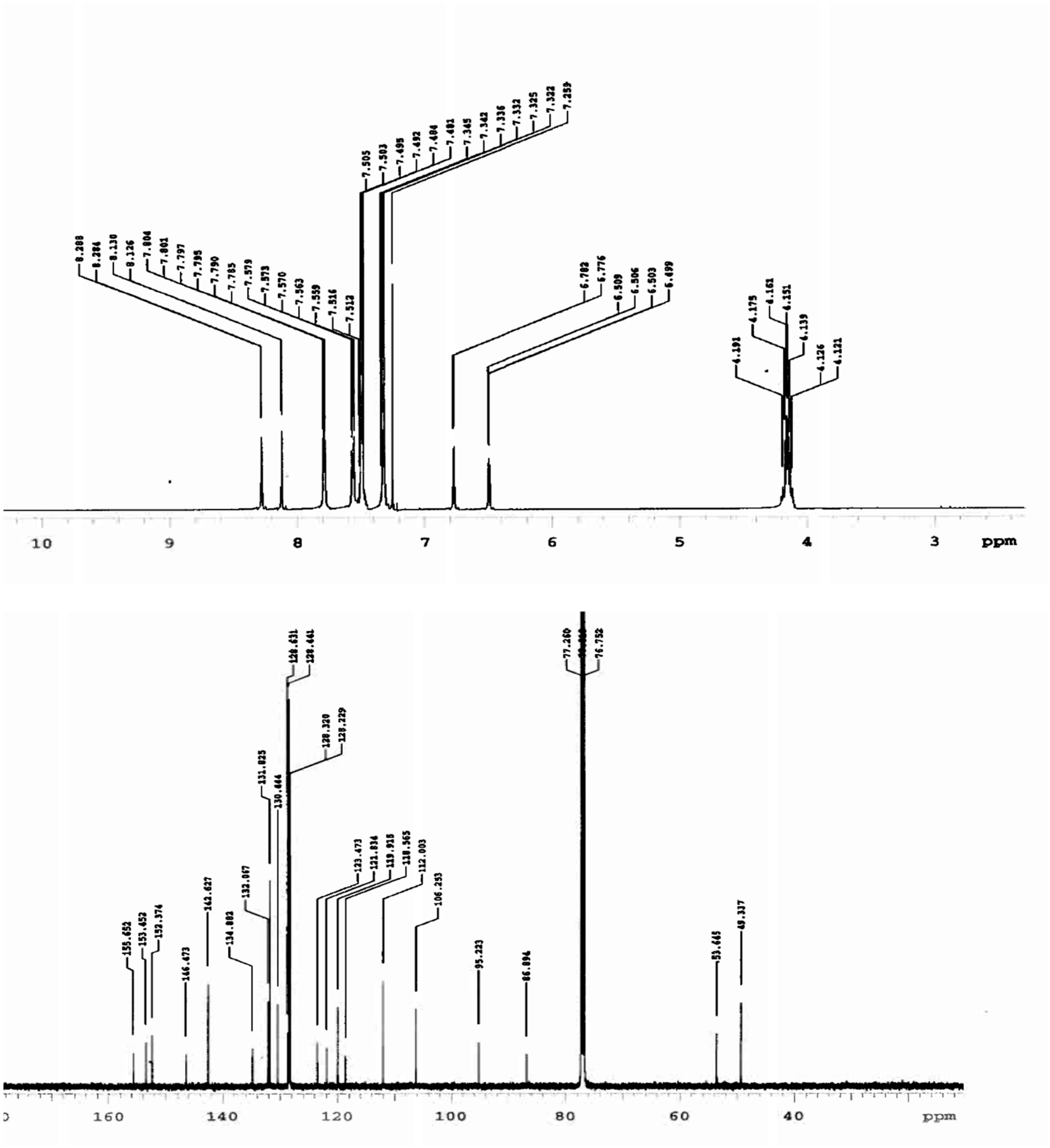

Figure S47.^{1}H and ^{13}C-NMR spectrum of **6a** in $CDCl_3$ at 500 and 125 MHz, respectively.

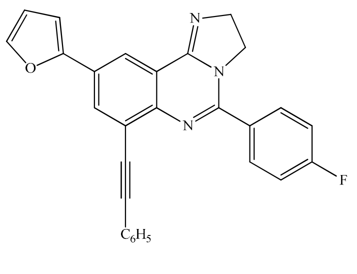

6b

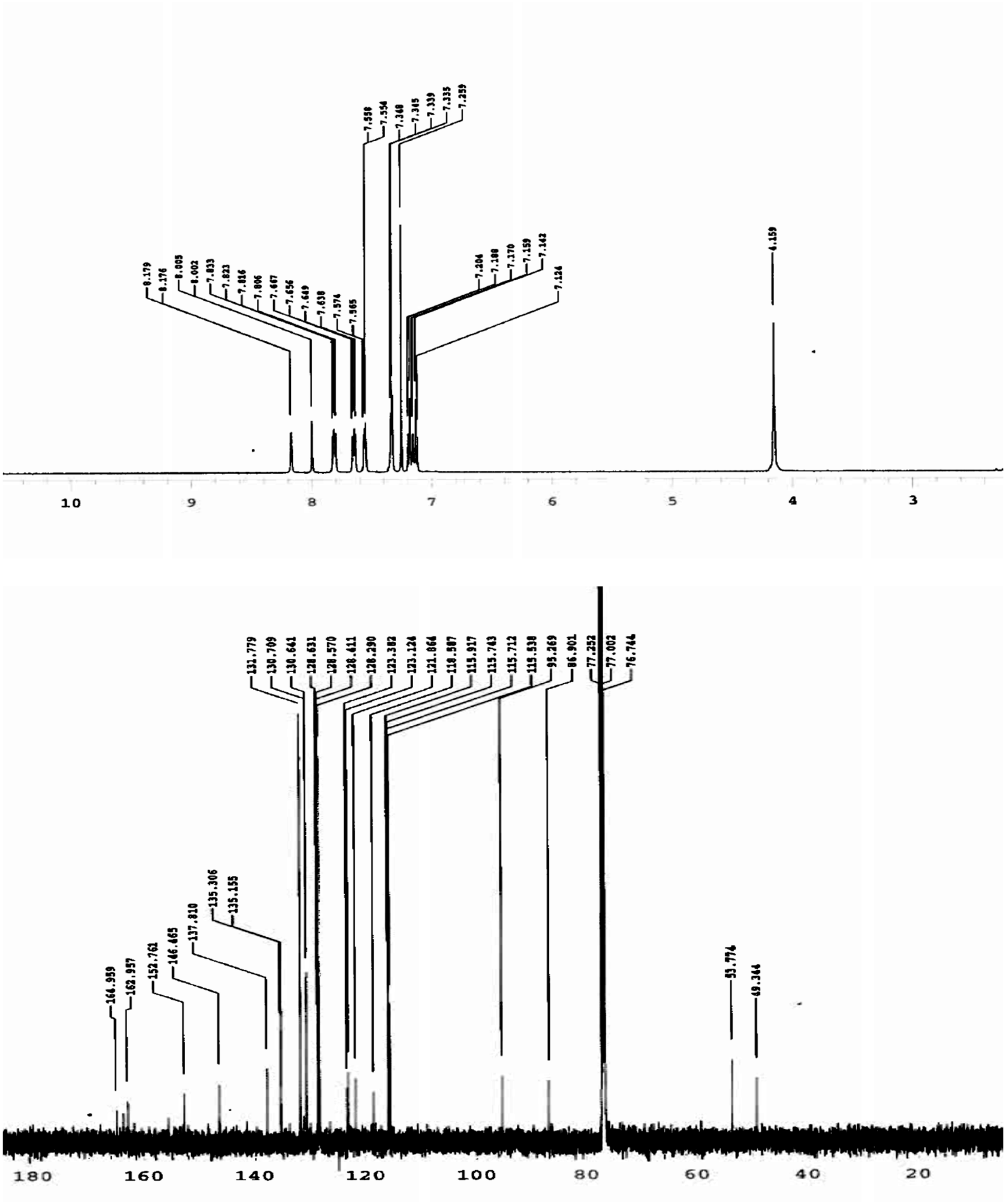

Figure S48.^{1}H and ^{13}C-NMR spectrum of **6b** in CDCl3 at 500 and 125 MHz, respectively.

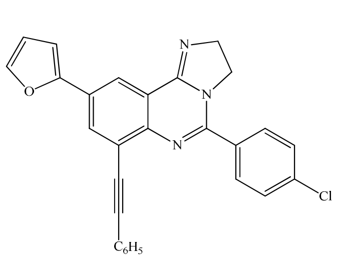

6c

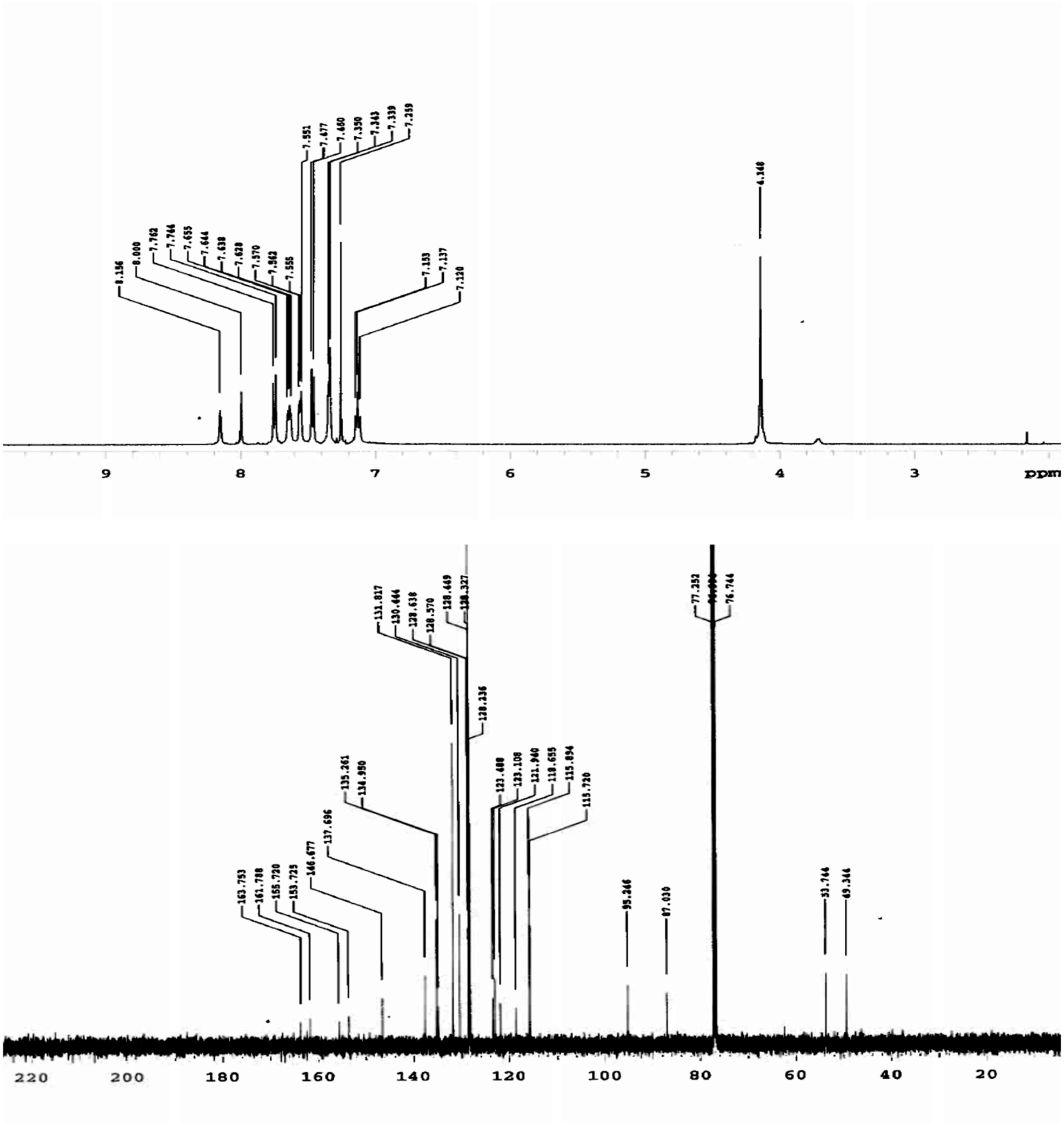

Figure S49.^{1}H and ^{13}C-NMR spectrum of **6c** in $CDCl_3$ at 500 and 125 MHz, respectively.

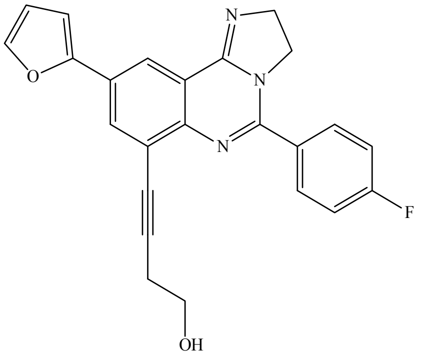

6d

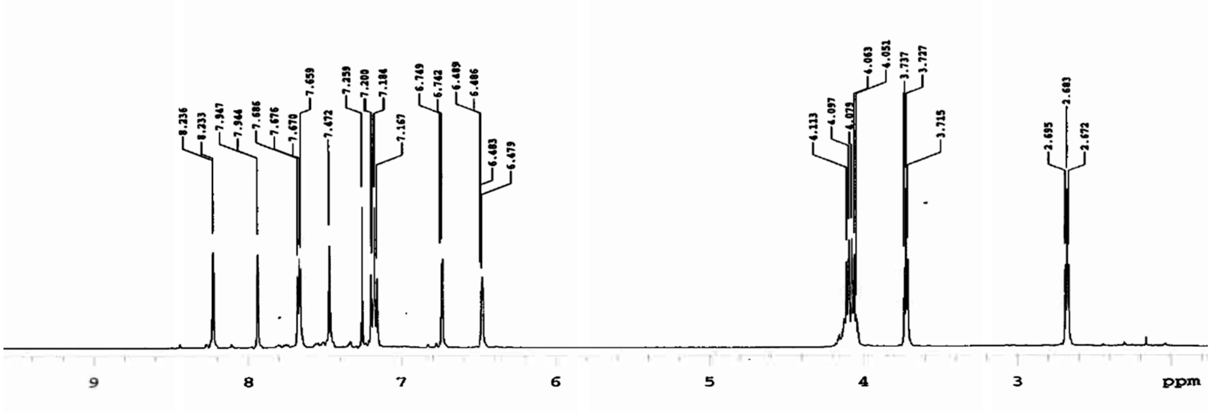

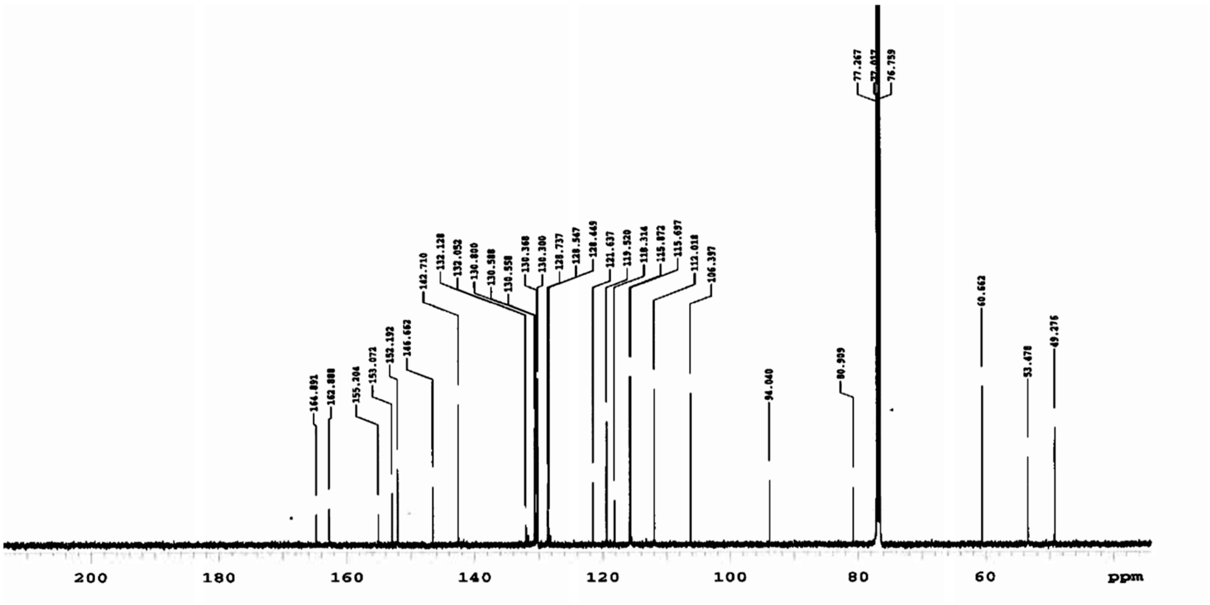

Figure S50.^{1}H and ^{13}C-NMR spectrum of **6d** in $CDCl_3$ at 500 and 125 MHz, respectively.

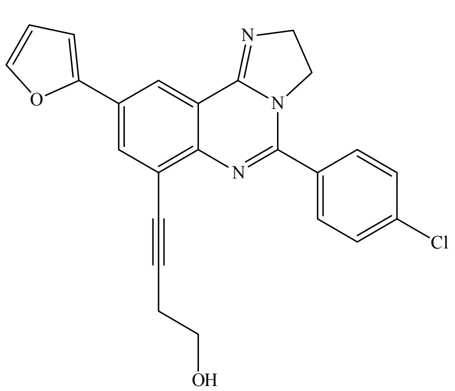

6e

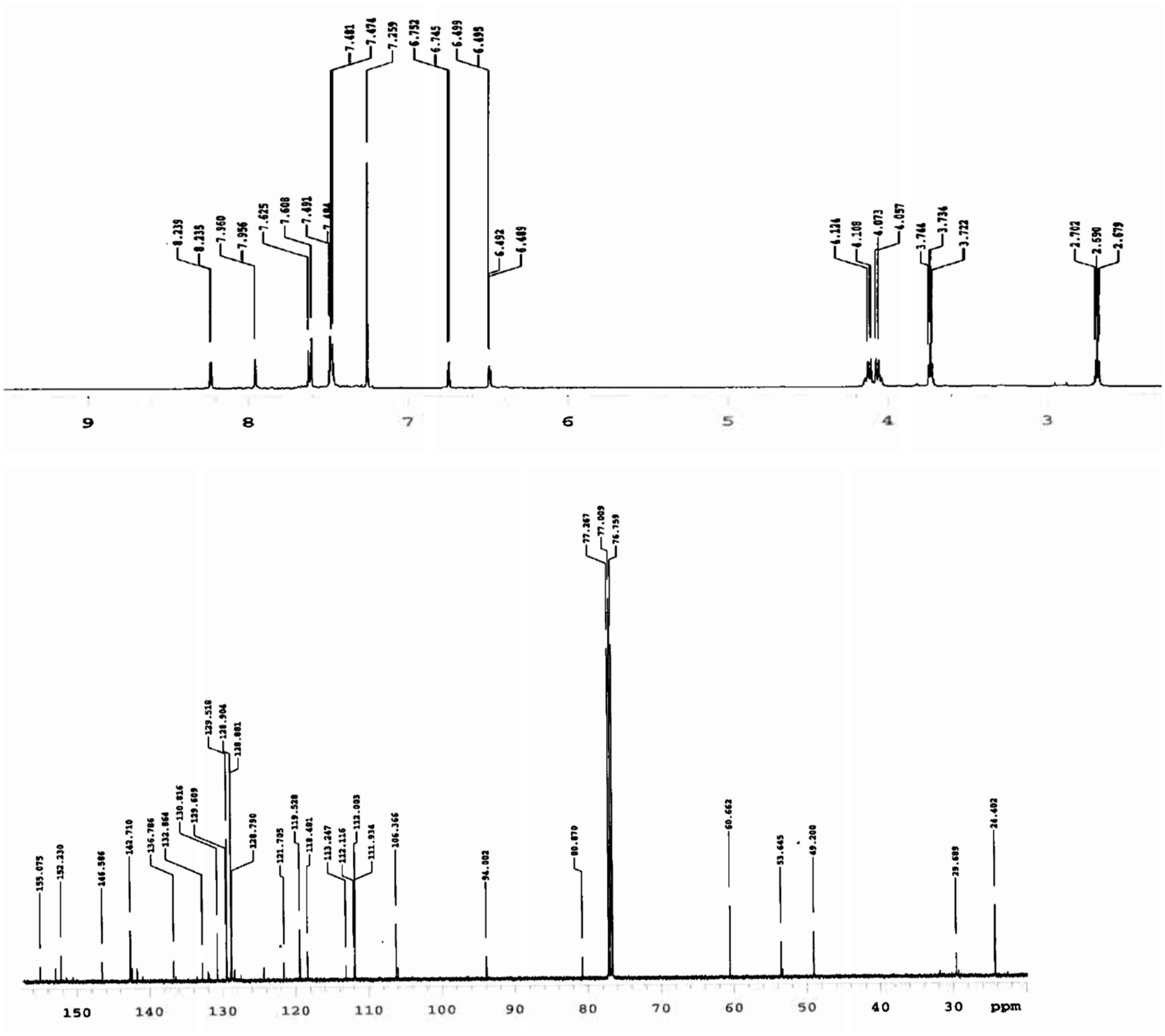

Figure S51. ^{1}H and ^{13}C-NMR spectrum of **6e** in $CDCl_3$ at 500 and 125 MHz, respectively.
